# Supplementary material for: A novel eukaryotic RdRP-dependent small RNA pathway represses antiviral immunity by controlling an ERK pathway component in the black-legged tick
Source: PLoS One. 2023 Mar 30;18(3):e0281195. doi: 10.1371/journal.pone.0281195 (PMC10062562; doi:10.1371/journal.pone.0281195)
Supplement: S1 Data — On the Normalized and Relative levels pages, normalized read counts (RPM) and relative levels compared to the control (dsGFP) library were used. TEs with more than 50RPM and Coding Genes with more than 3.5RPM on average in KD libraries are included in this website. For viral sequences, reference sequences were generated by assembling sRNA sequences (See the Analysis of sRNAseq data section in Materials and methods.) and size distributions of sRNA reads mapped to each contig are shown. Reads were first grouped by their 5’ nucleotides, and reads of each length were counted. Full tables including TEs and Coding genes with fewer reads are in the “FullTable” folder. (ZIP) [file pone.0281195.s017.zip › SupplementaryData/Links/PDFs/21-23_TE_readcount_libs.pdf]

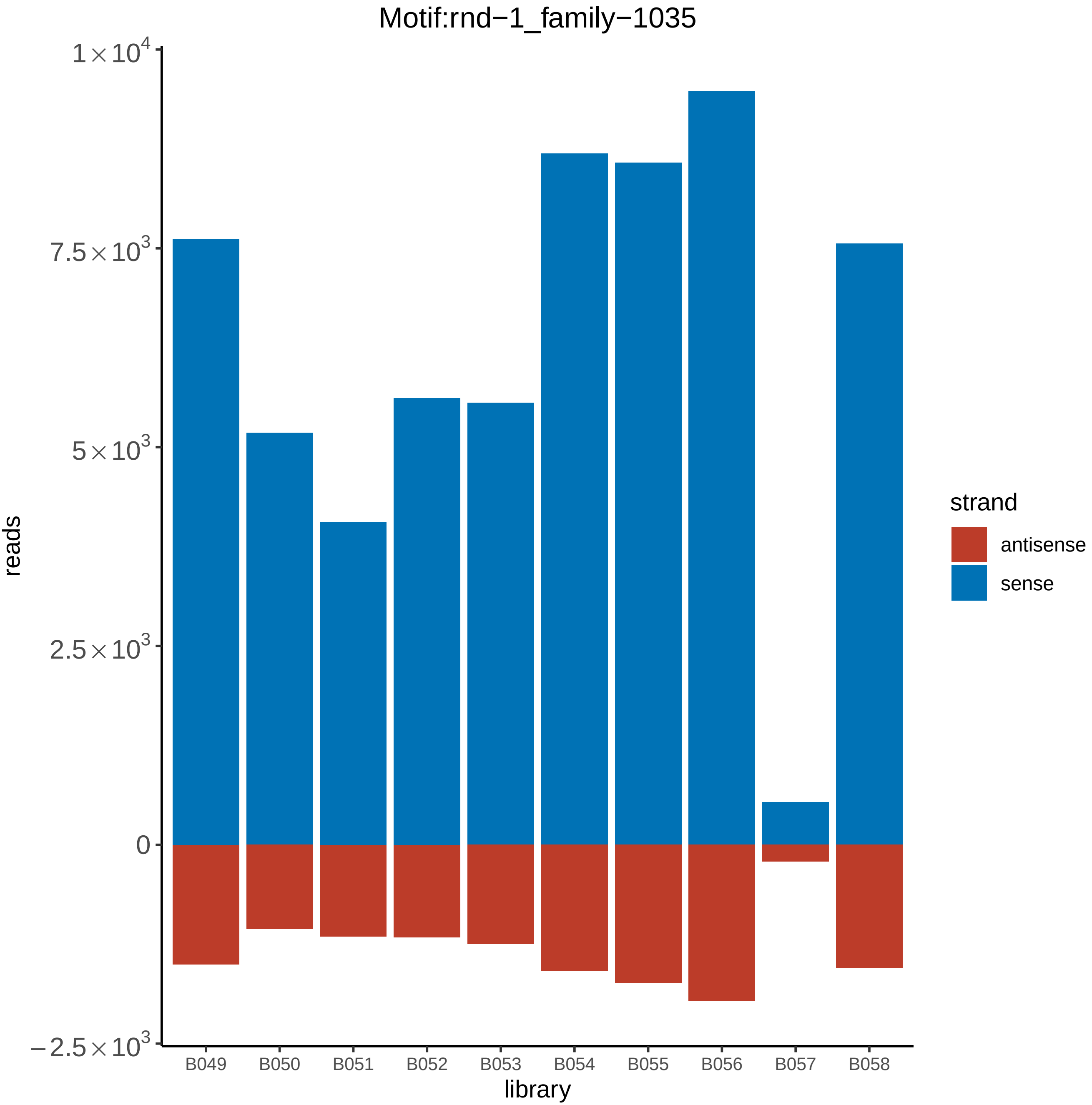

Motif:rnd−1_family−1035
1×10^4
7.5×10^3
5×10^3
2.5×10^3
0
-2.5×10^3
reads
B049
B050
B051
B052
B053
B054
B055
B056
B057
B058
library
strand
antisense
sense

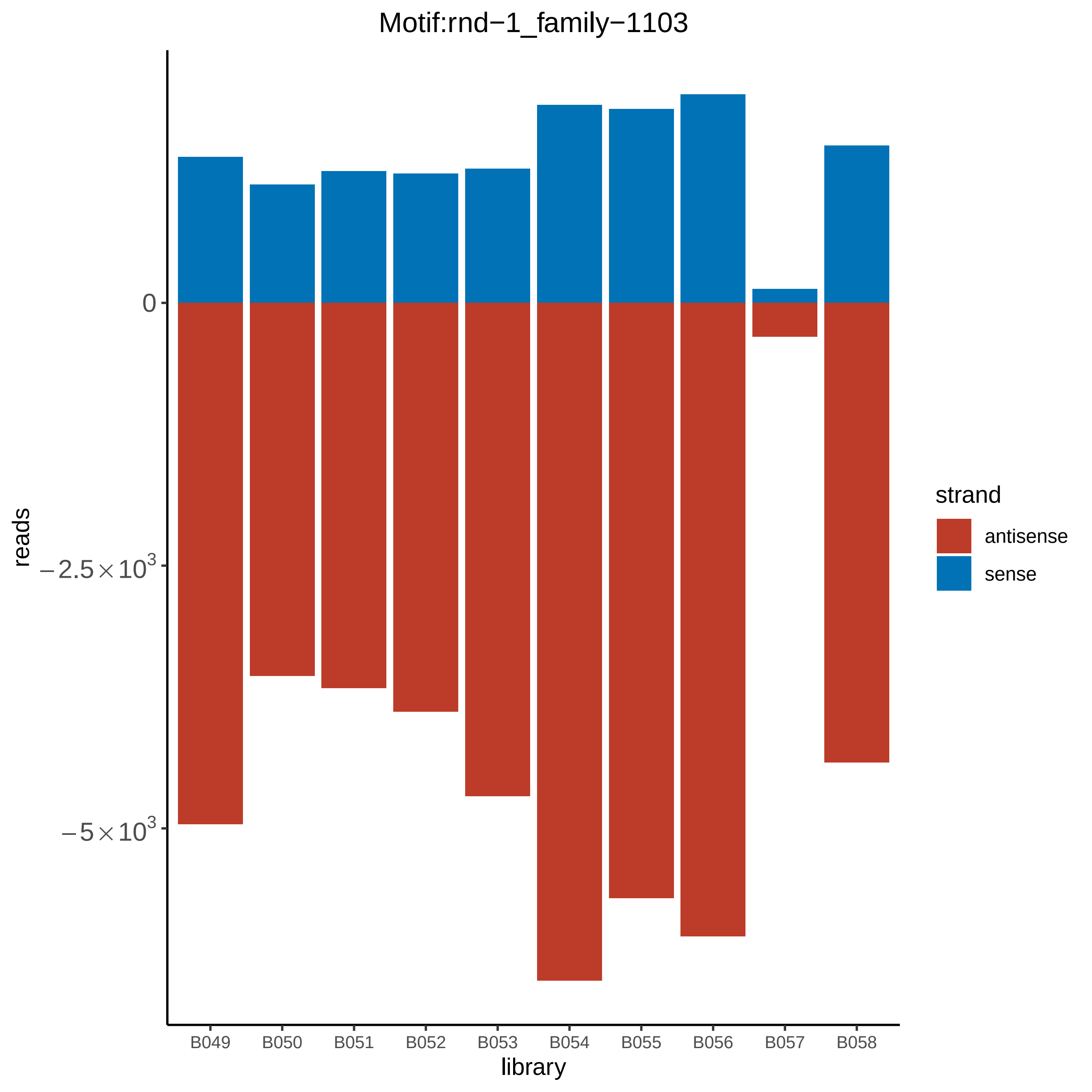
Motif:rnd−1_family−1103
reads
0
−2.5×10^3
−5×10^3
library
B049
B050
B051
B052
B053
B054
B055
B056
B057
B058
strand
antisense
sense

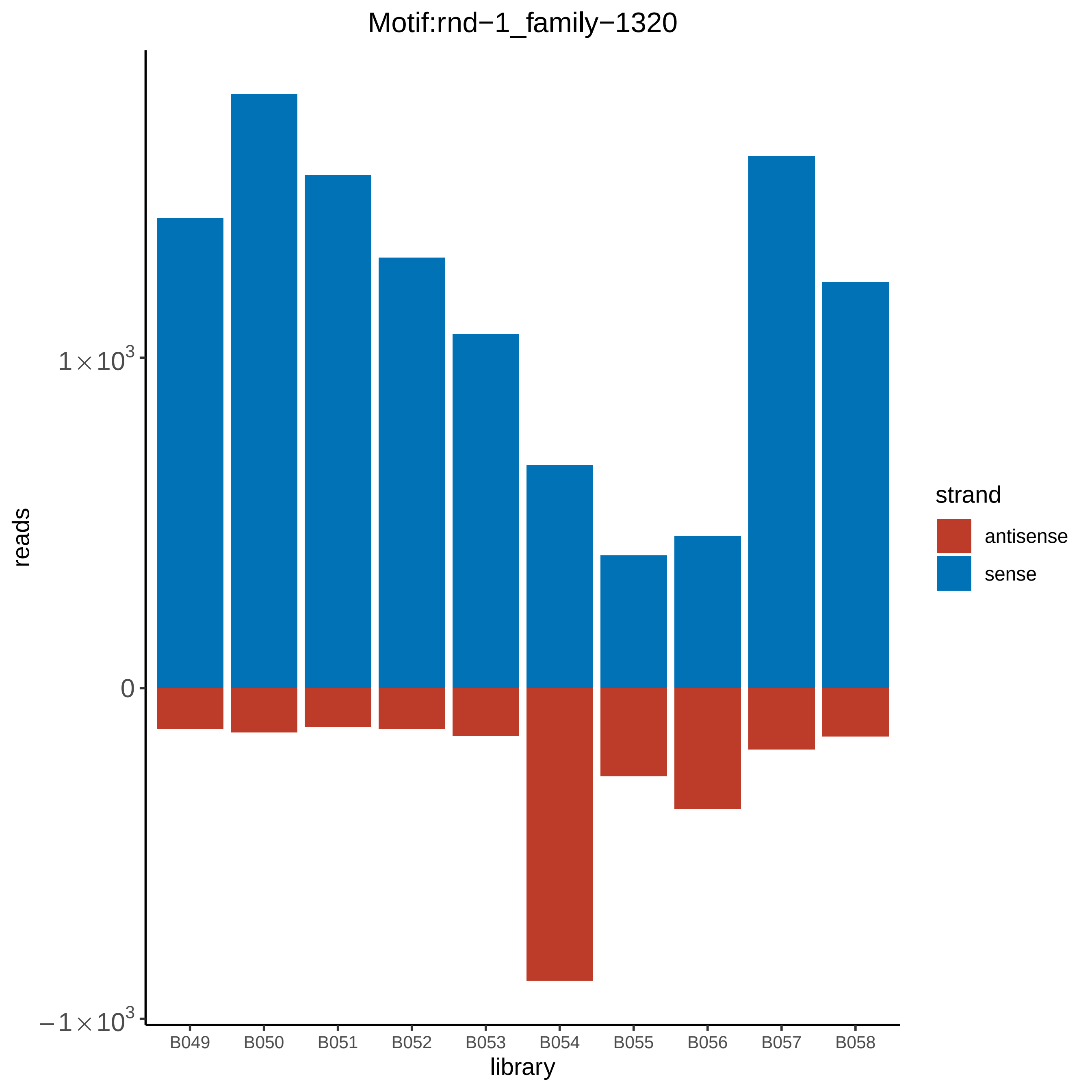

Motif:rnd−1_family−1320
reads
library
1×10^3
0
-1×10^3
B049
B050
B051
B052
B053
B054
B055
B056
B057
B058
strand
antisense
sense

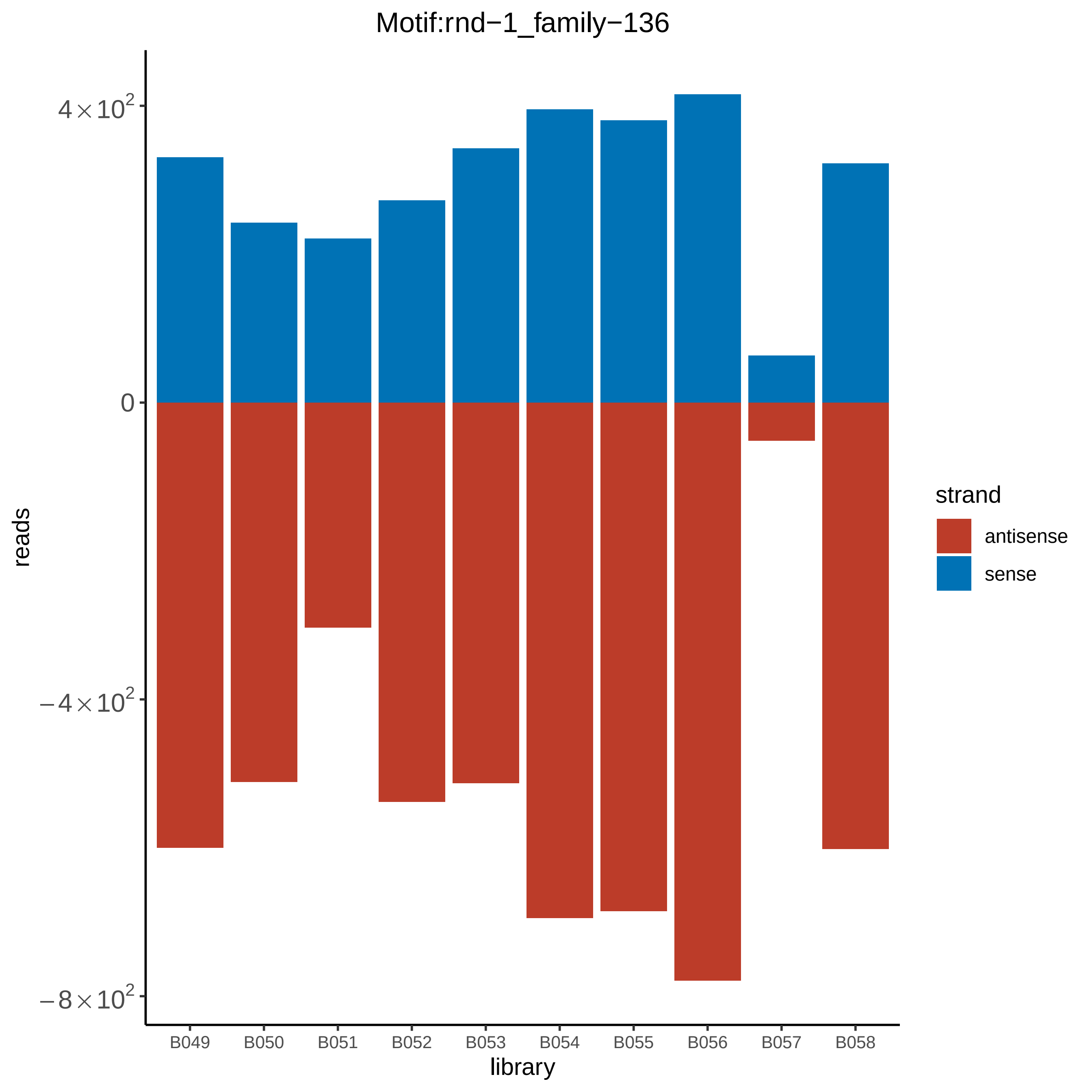

Motif:rnd−1_family−136
reads
4×10^2
0
-4×10^2
-8×10^2
library
B049
B050
B051
B052
B053
B054
B055
B056
B057
B058
strand
antisense
sense

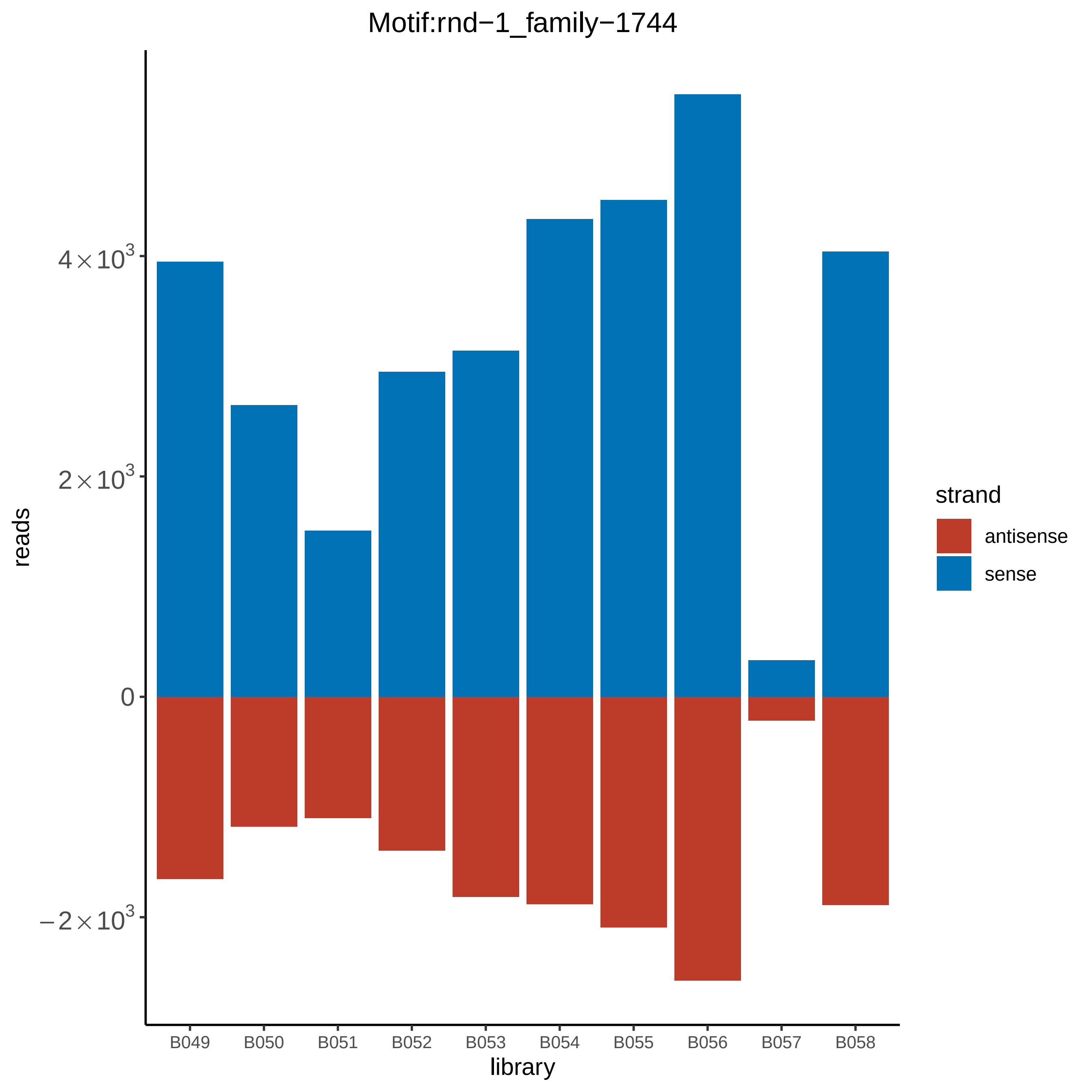

Motif:rnd−1_family−1744
reads
4×10^3
2×10^3
0
-2×10^3
B049
B050
B051
B052
B053
B054
B055
B056
B057
B058
library
strand
antisense
sense

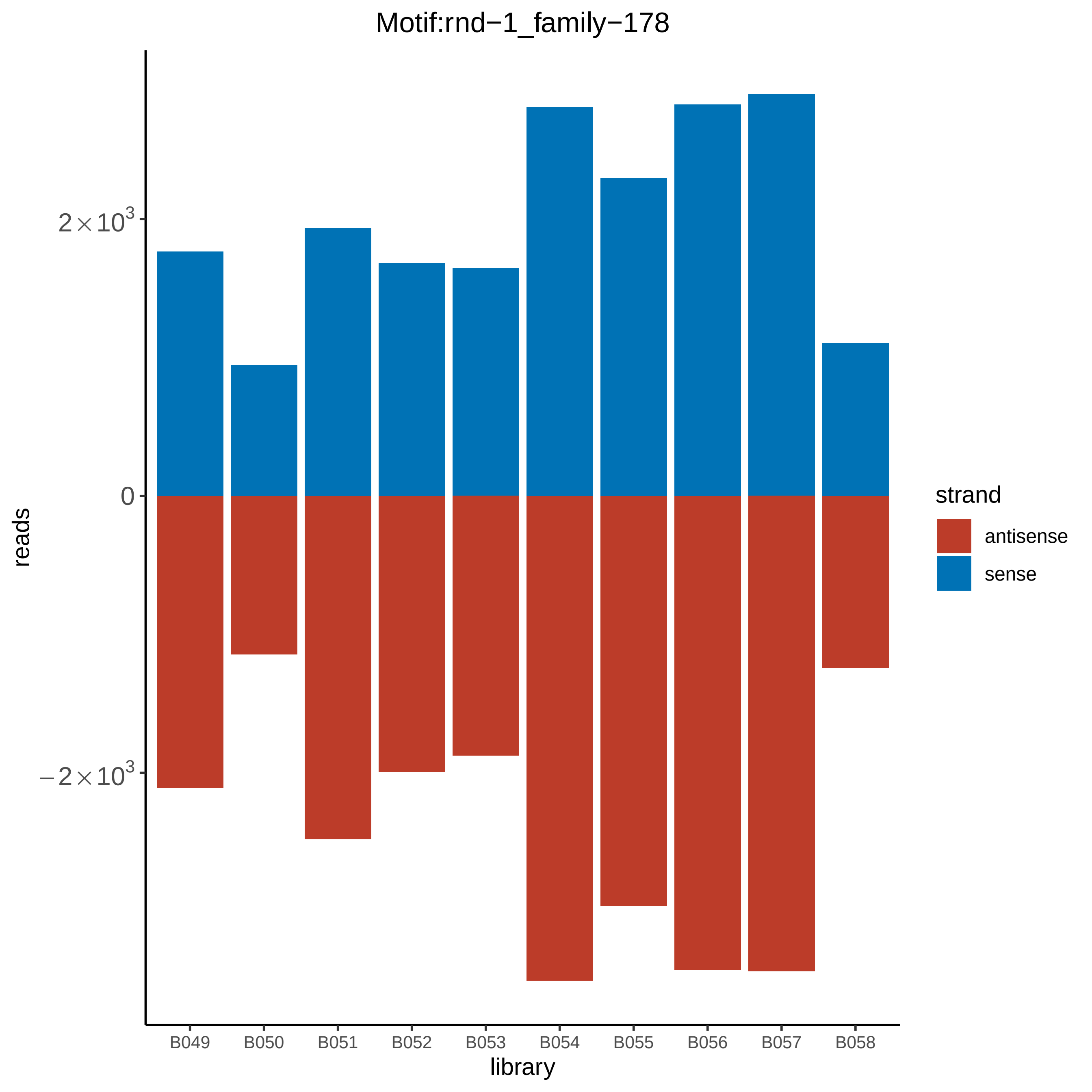

Motif:rnd−1_family−178
2×10^3
0
−2×10^3
reads
B049
B050
B051
B052
B053
B054
B055
B056
B057
B058
library
strand
antisense
sense

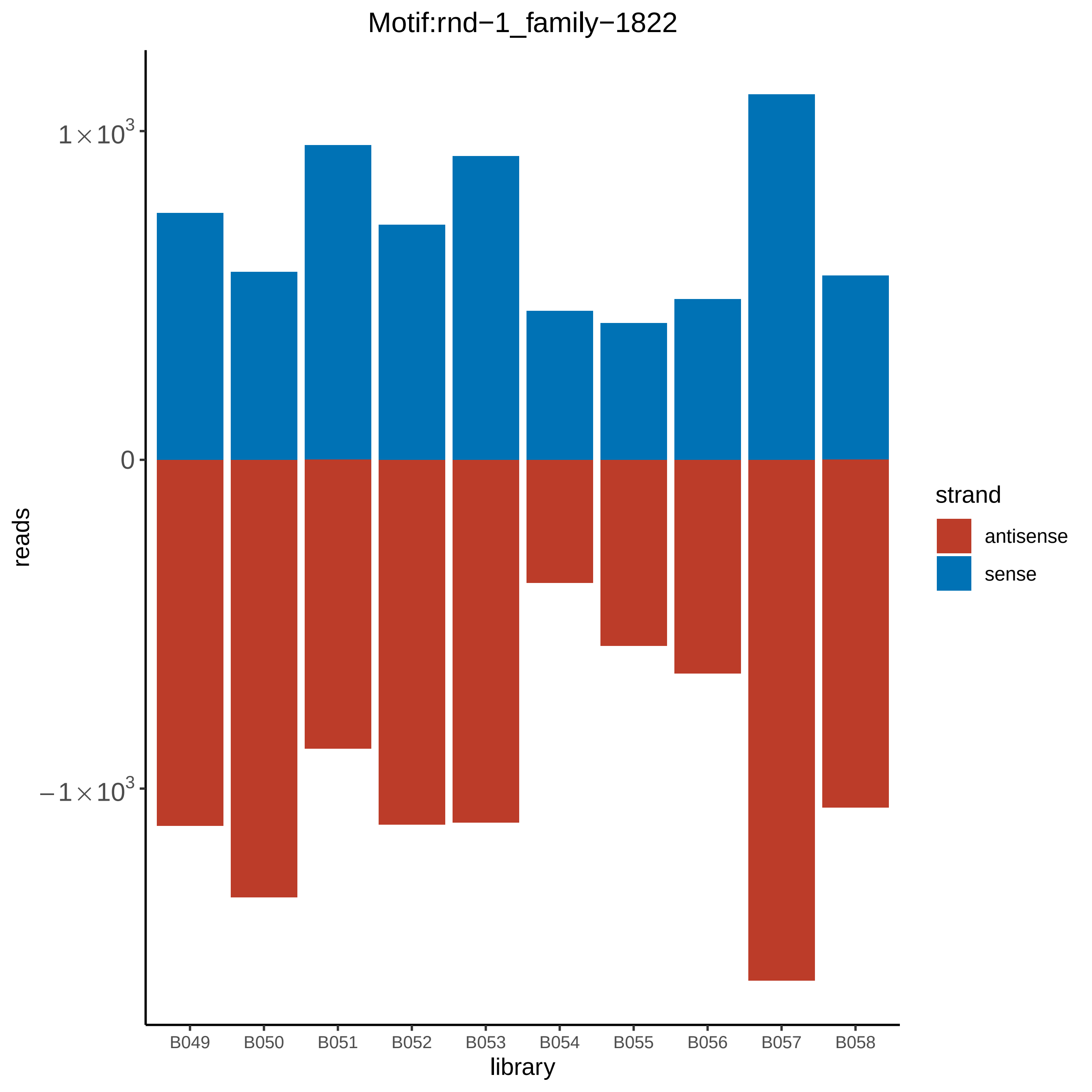

Motif:rnd−1_family−1822
reads
1×10^3
0
-1×10^3
library
B049
B050
B051
B052
B053
B054
B055
B056
B057
B058
strand
antisense
sense

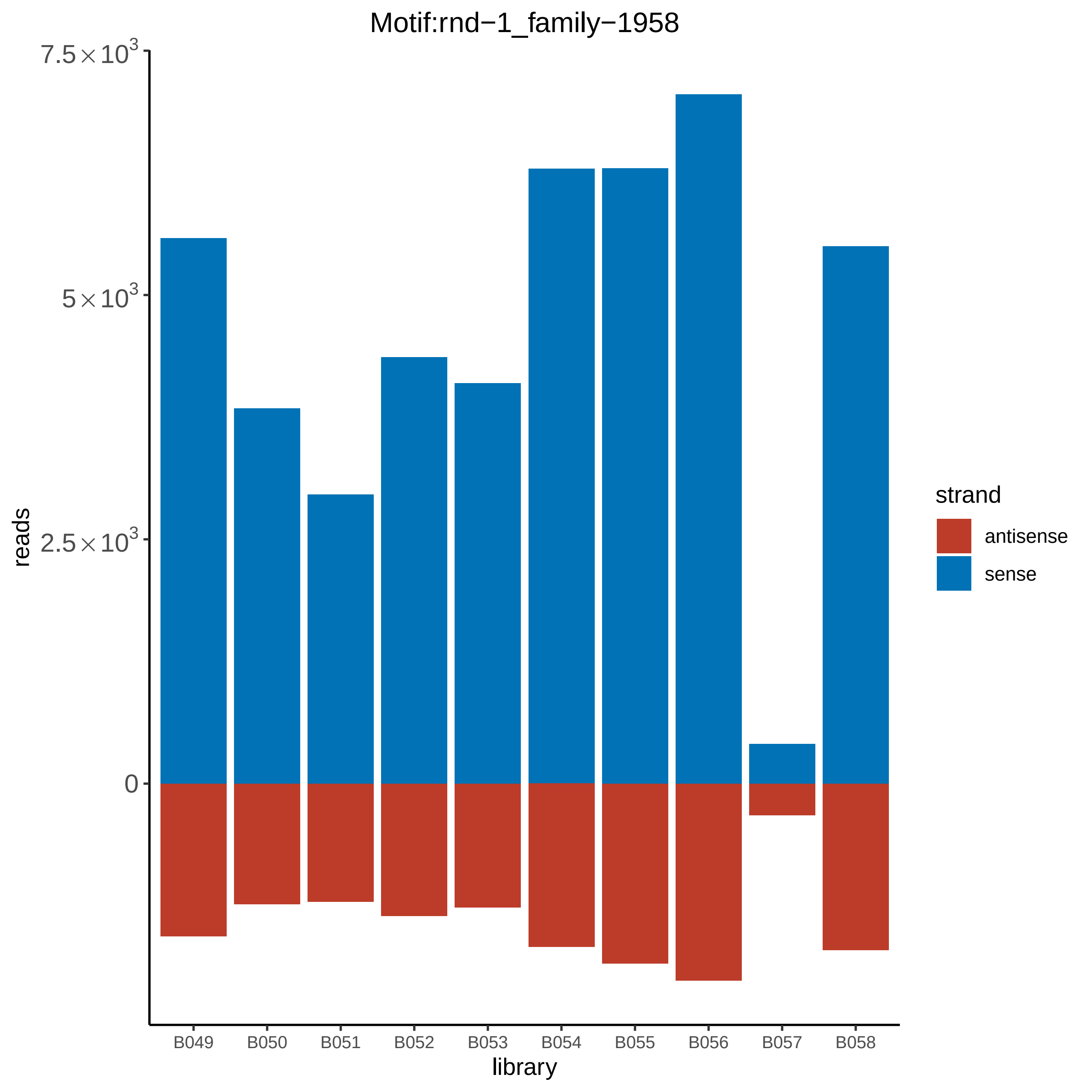

Motif:rnd−1_family−1958
7.5×10^3
5×10^3
2.5×10^3
0
reads
B049
B050
B051
B052
B053
B054
B055
B056
B057
B058
library
strand
antisense
sense

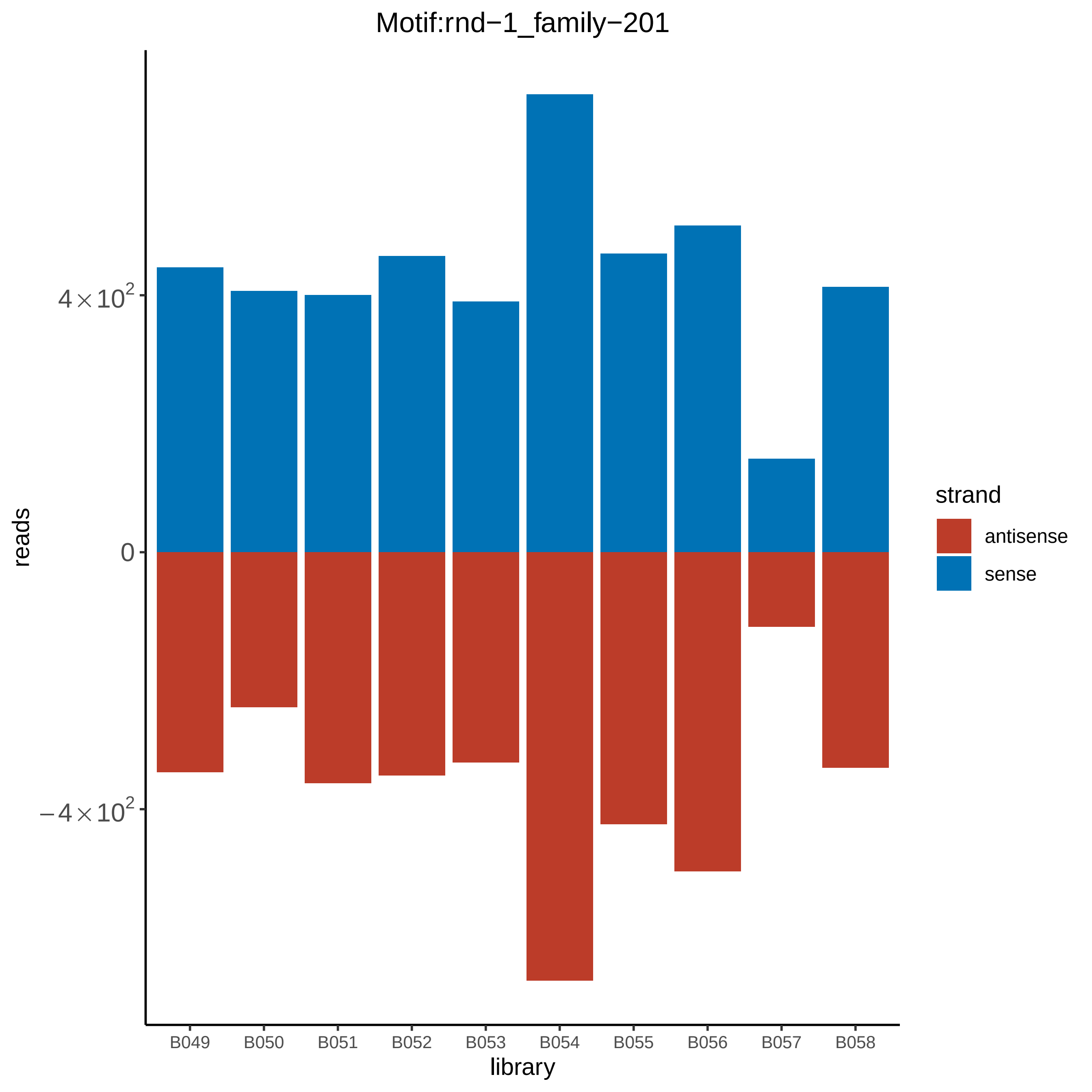
Motif:rnd−1_family−201
reads
4×10^2
0
−4×10^2
library
B049
B050
B051
B052
B053
B054
B055
B056
B057
B058
strand
antisense
sense

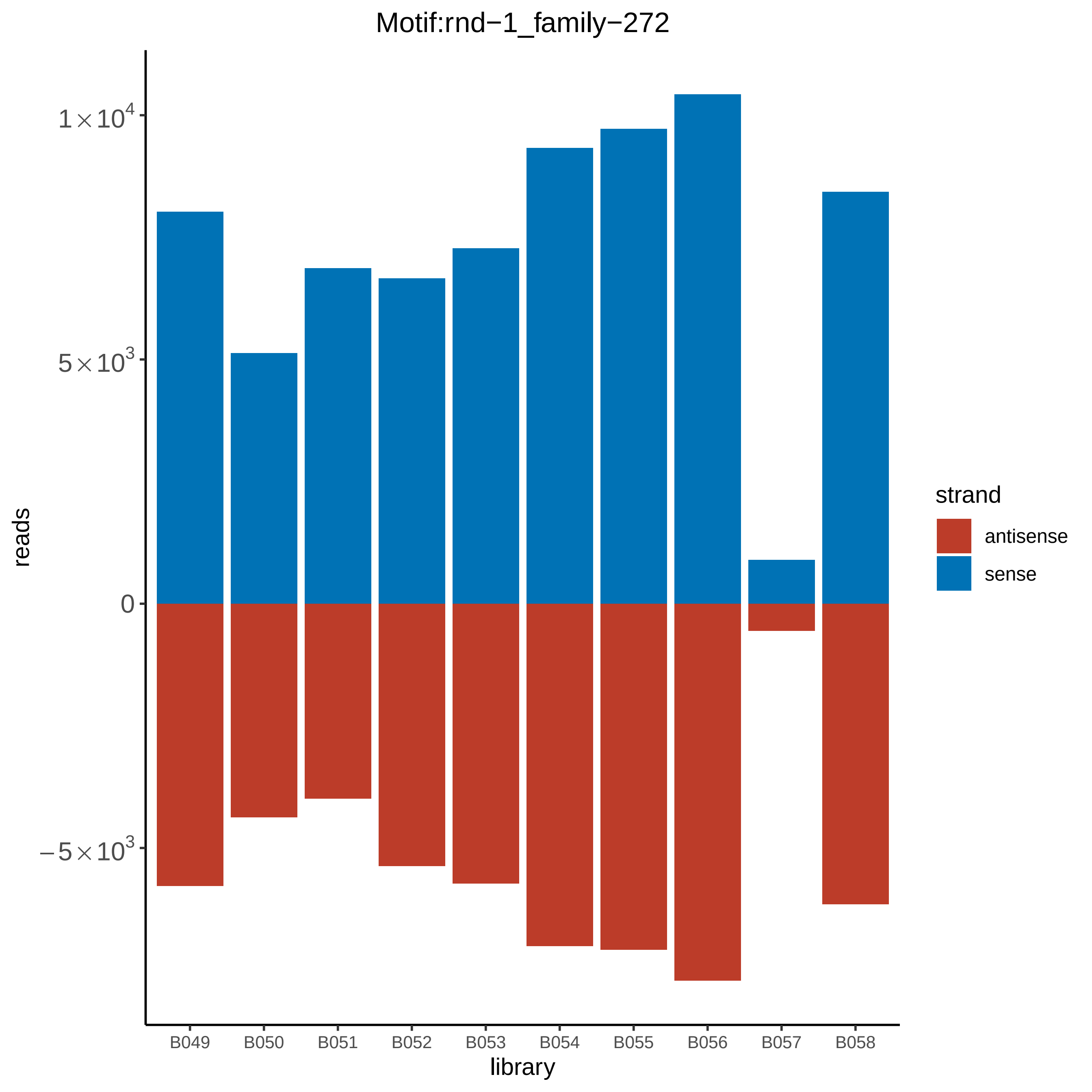
Motif:rnd−1_family−272
reads
1×10^4
5×10^3
0
-5×10^3
library
B049
B050
B051
B052
B053
B054
B055
B056
B057
B058
strand
antisense
sense

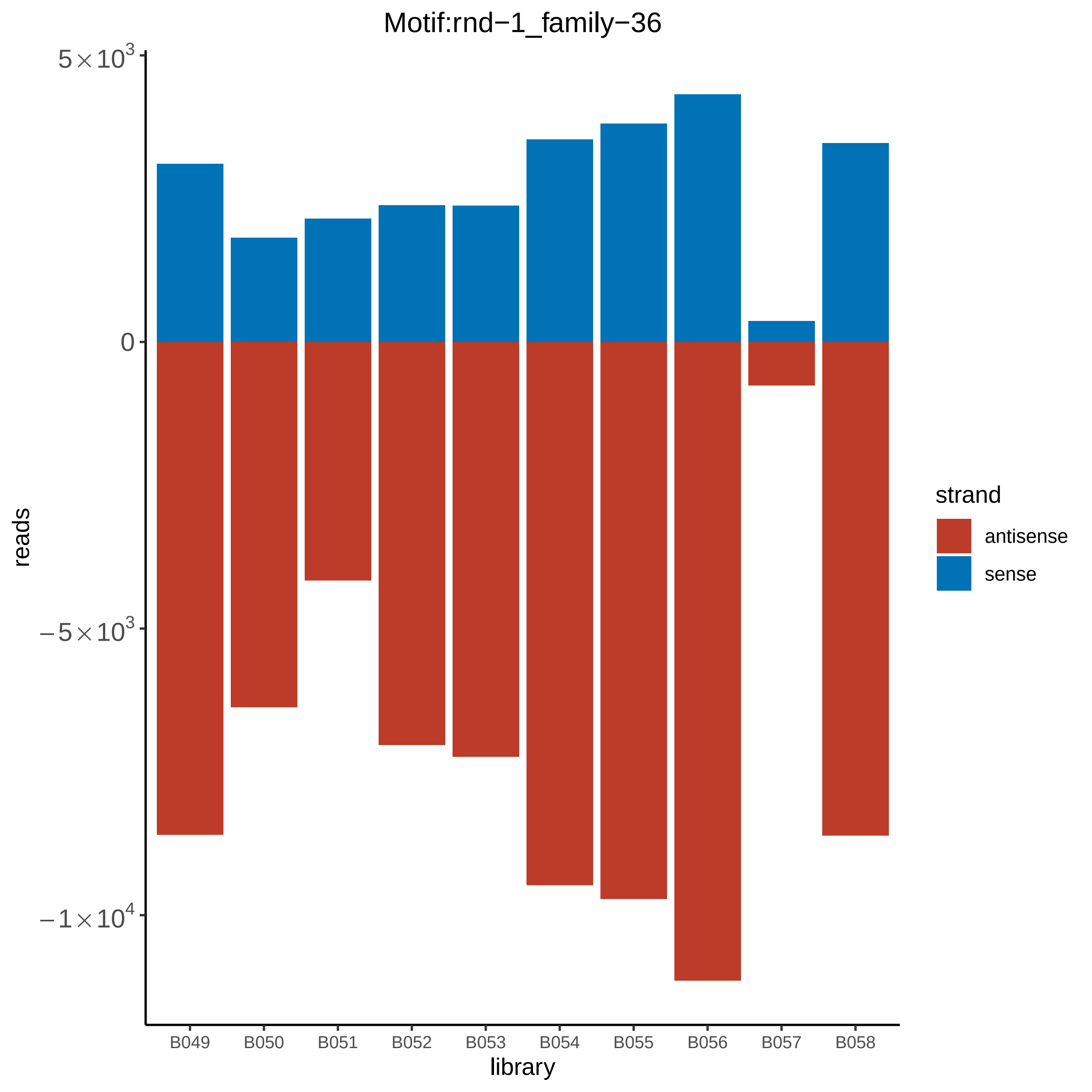
Motif:rnd−1_family−36
5×10^3
0
-5×10^3
-1×10^4
reads
B049
B050
B051
B052
B053
B054
B055
B056
B057
B058
library
strand
antisense
sense

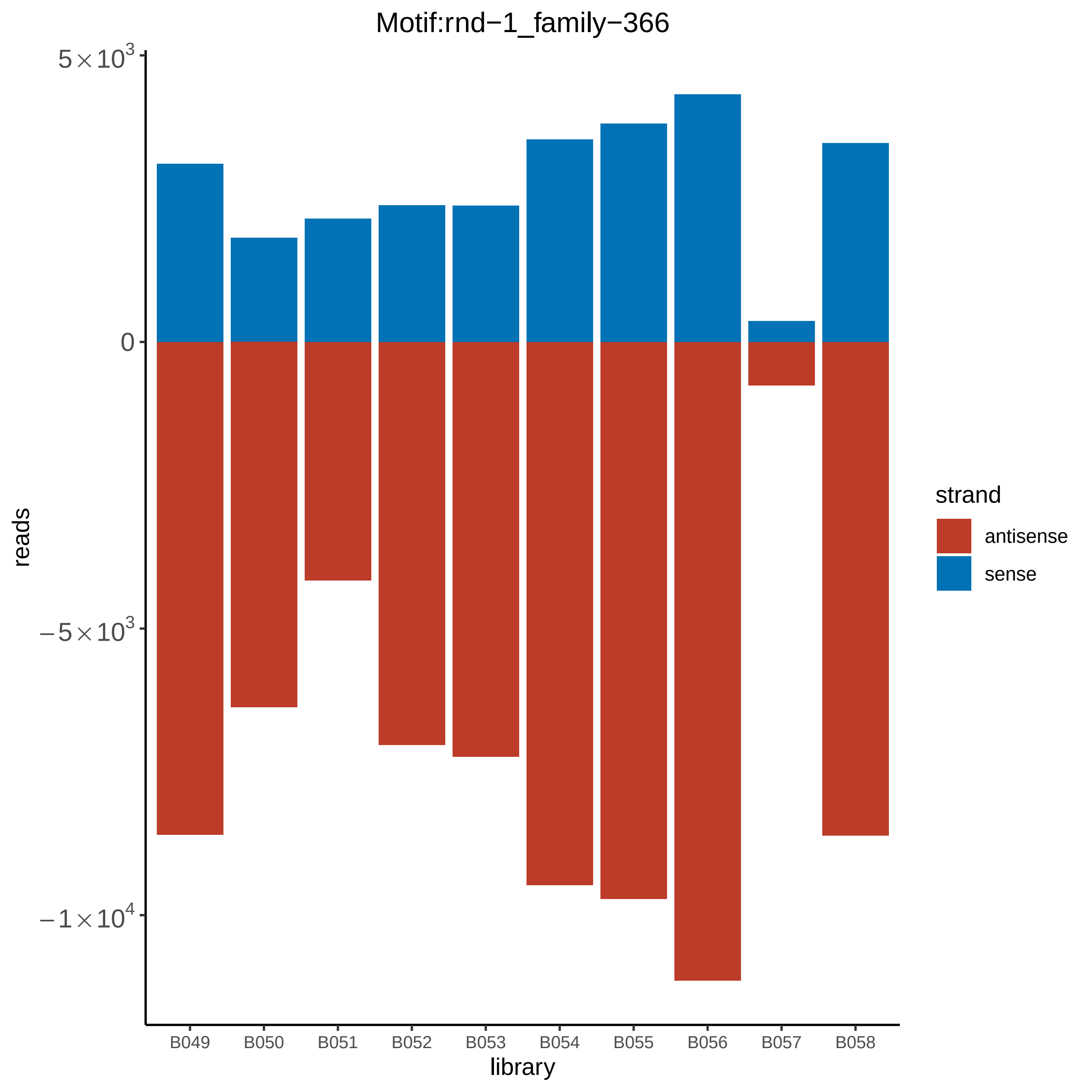
Motif:rnd−1_family−366
5×10^3
0
−5×10^3
−1×10^4
reads
B049
B050
B051
B052
B053
B054
B055
B056
B057
B058
library
strand
antisense
sense

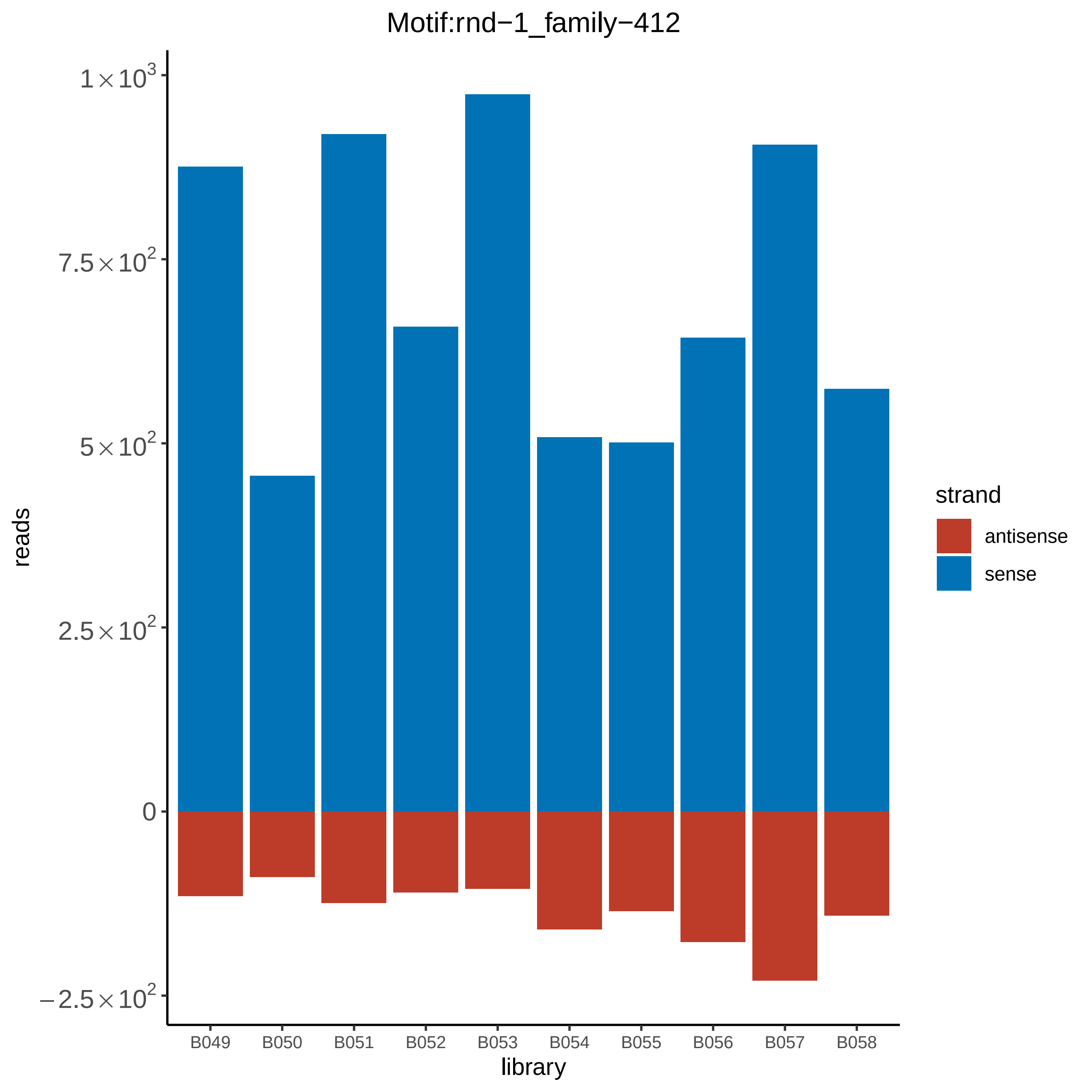

Motif:rnd−1_family−412
reads
1×10^3
7.5×10^2
5×10^2
2.5×10^2
0
-2.5×10^2
B049
B050
B051
B052
B053
B054
B055
B056
B057
B058
library
strand
antisense
sense

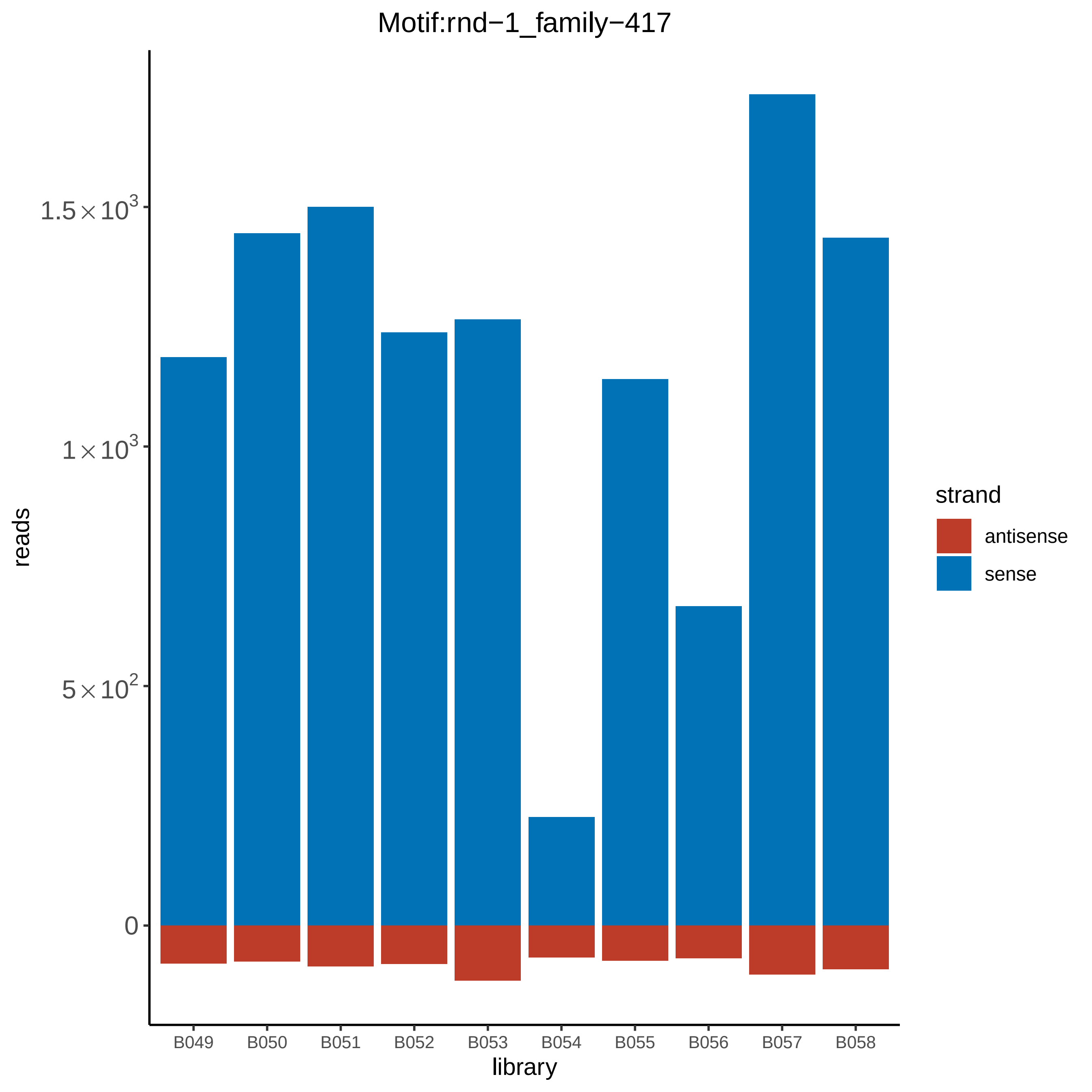
Motif:rnd−1_family−417
reads
1.5×10^3
1×10^3
5×10^2
0
library
B049
B050
B051
B052
B053
B054
B055
B056
B057
B058
strand
antisense
sense

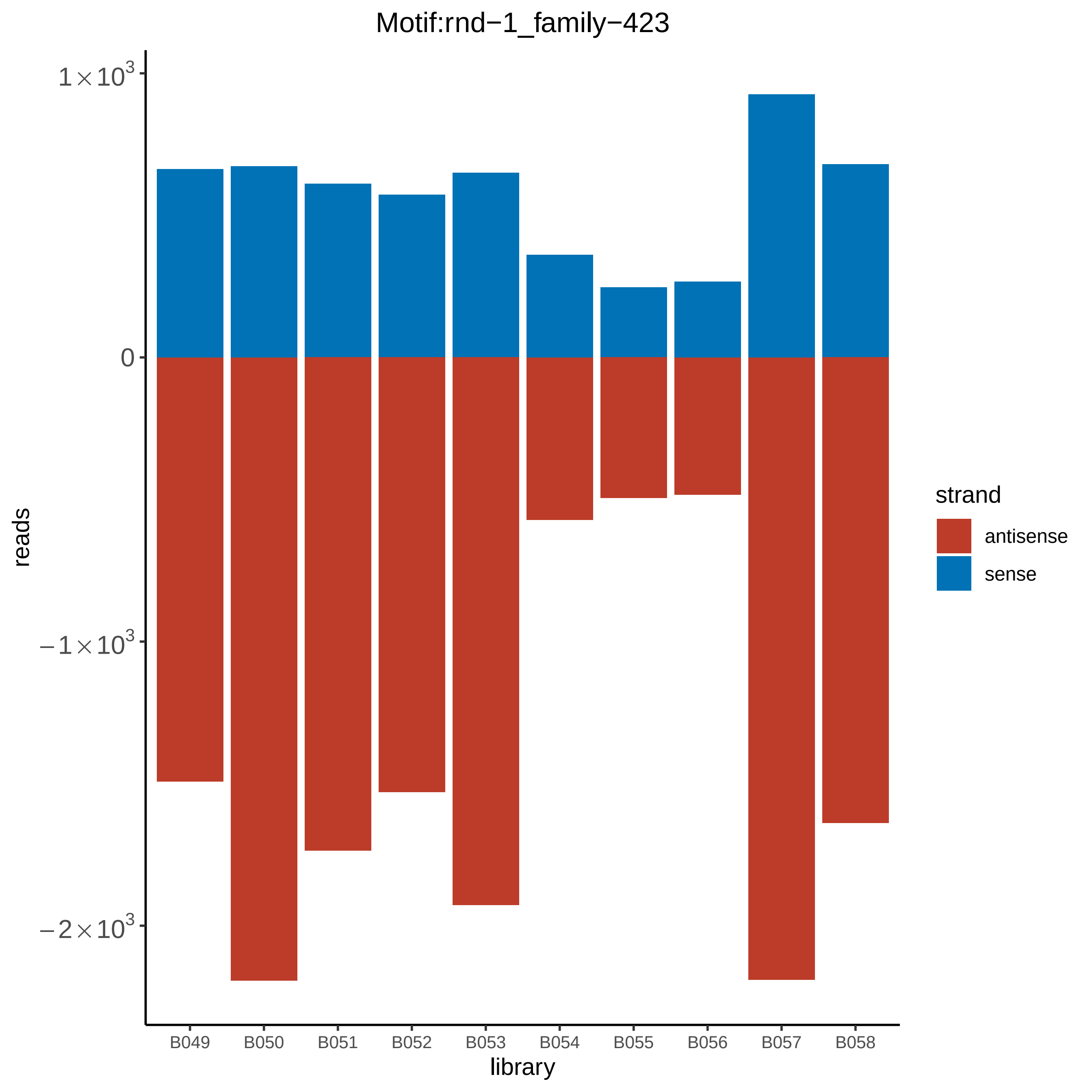

Motif:rnd−1_family−423
reads
1×10^3
0
-1×10^3
-2×10^3
B049
B050
B051
B052
B053
B054
B055
B056
B057
B058
library
strand
antisense
sense

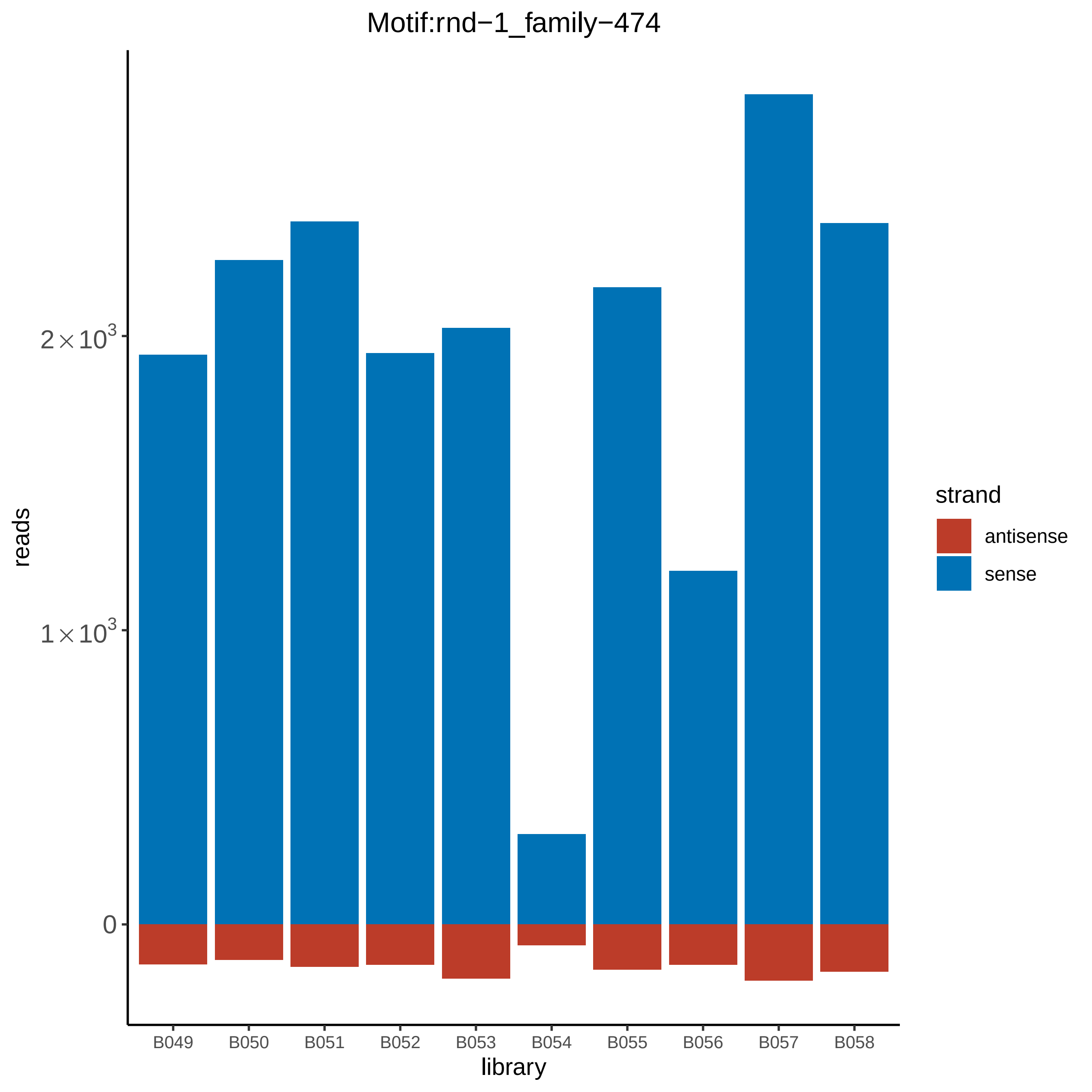
Motif:rnd−1_family−474
reads
2×10^3
1×10^3
0
library
B049
B050
B051
B052
B053
B054
B055
B056
B057
B058
strand
antisense
sense

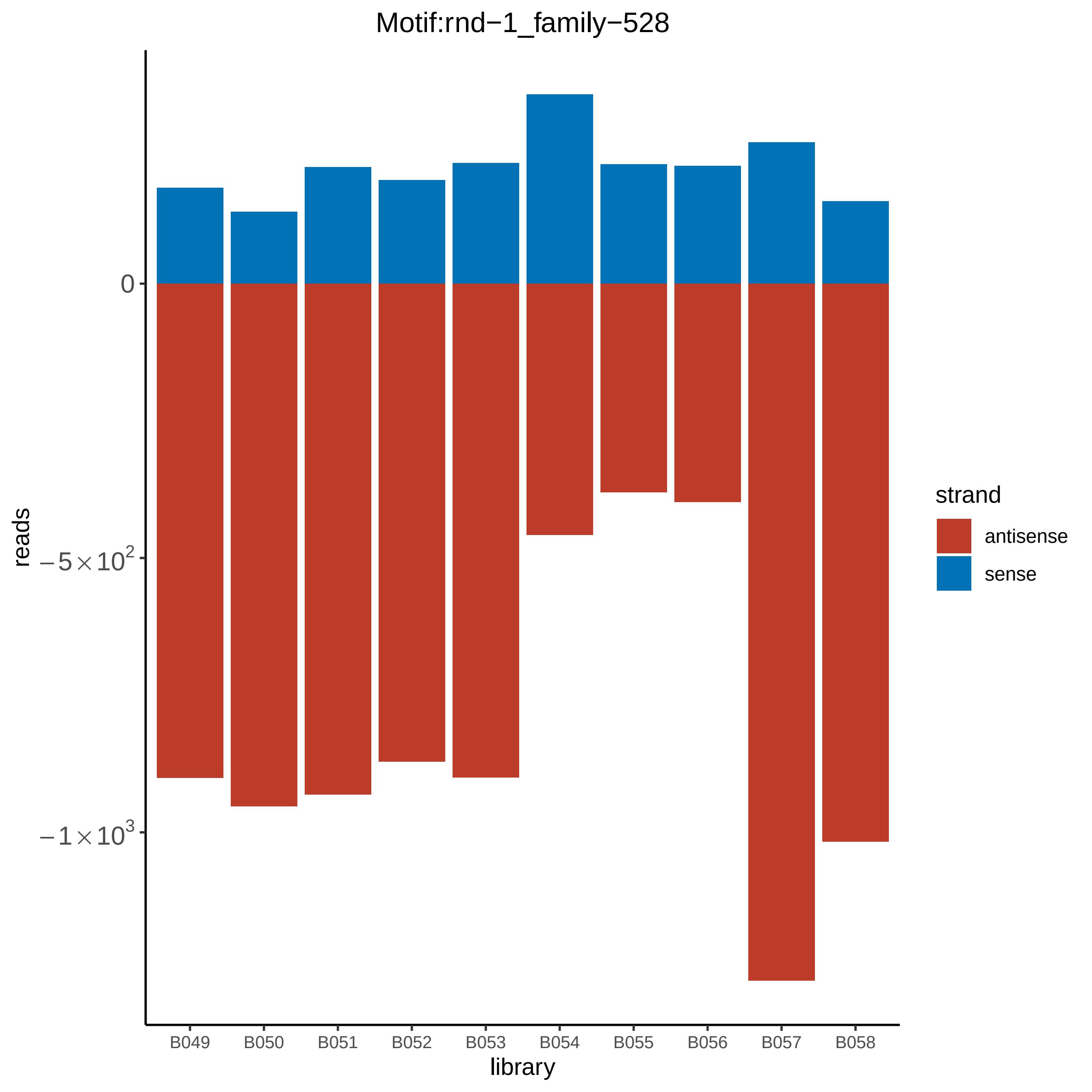

Motif:rnd−1_family−528
reads
0
−5×10^2
−1×10^3
library
B049
B050
B051
B052
B053
B054
B055
B056
B057
B058
strand
antisense
sense

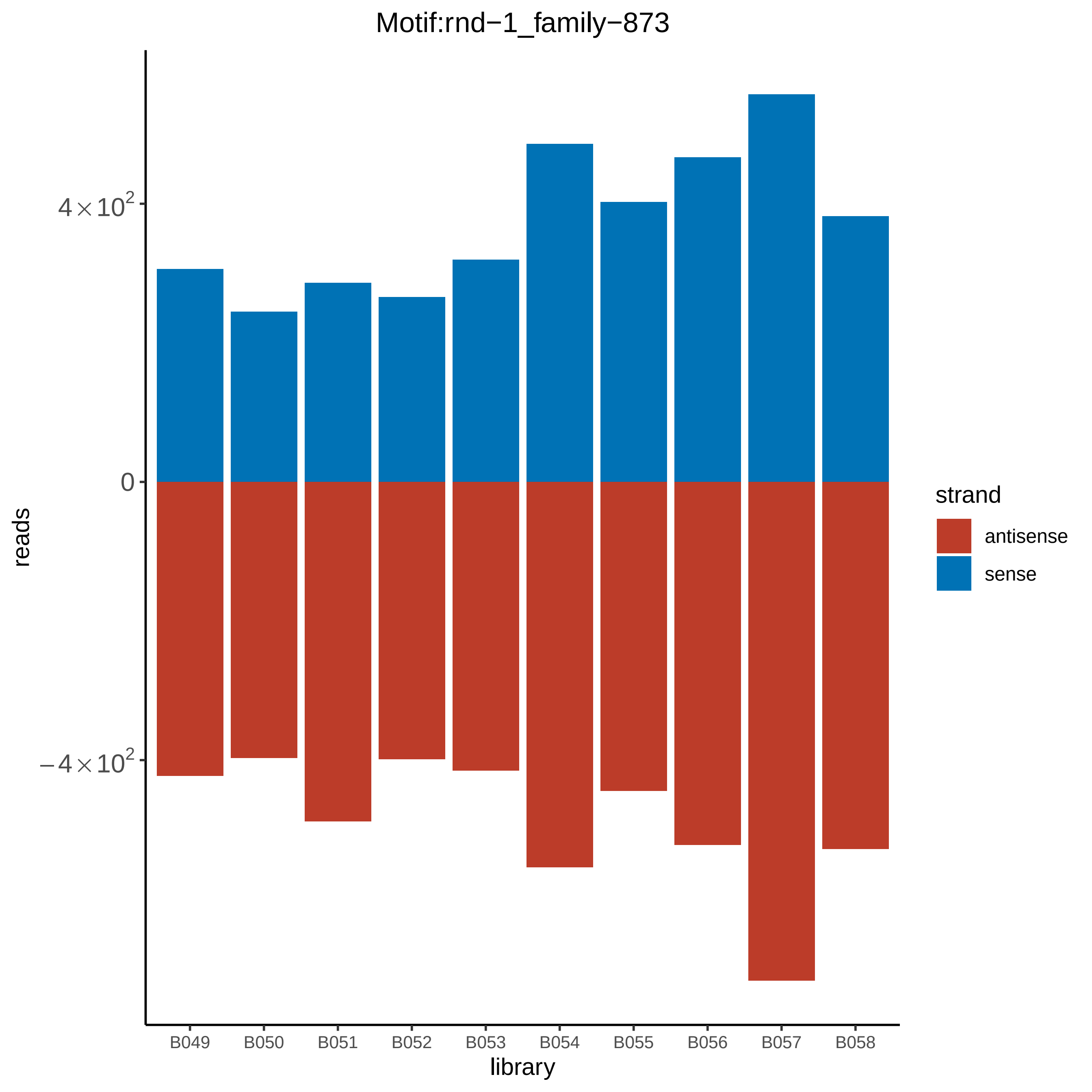

Motif:rnd−1_family−873
reads
4×10^2
0
-4×10^2
B049
B050
B051
B052
B053
B054
B055
B056
B057
B058
library
strand
antisense
sense

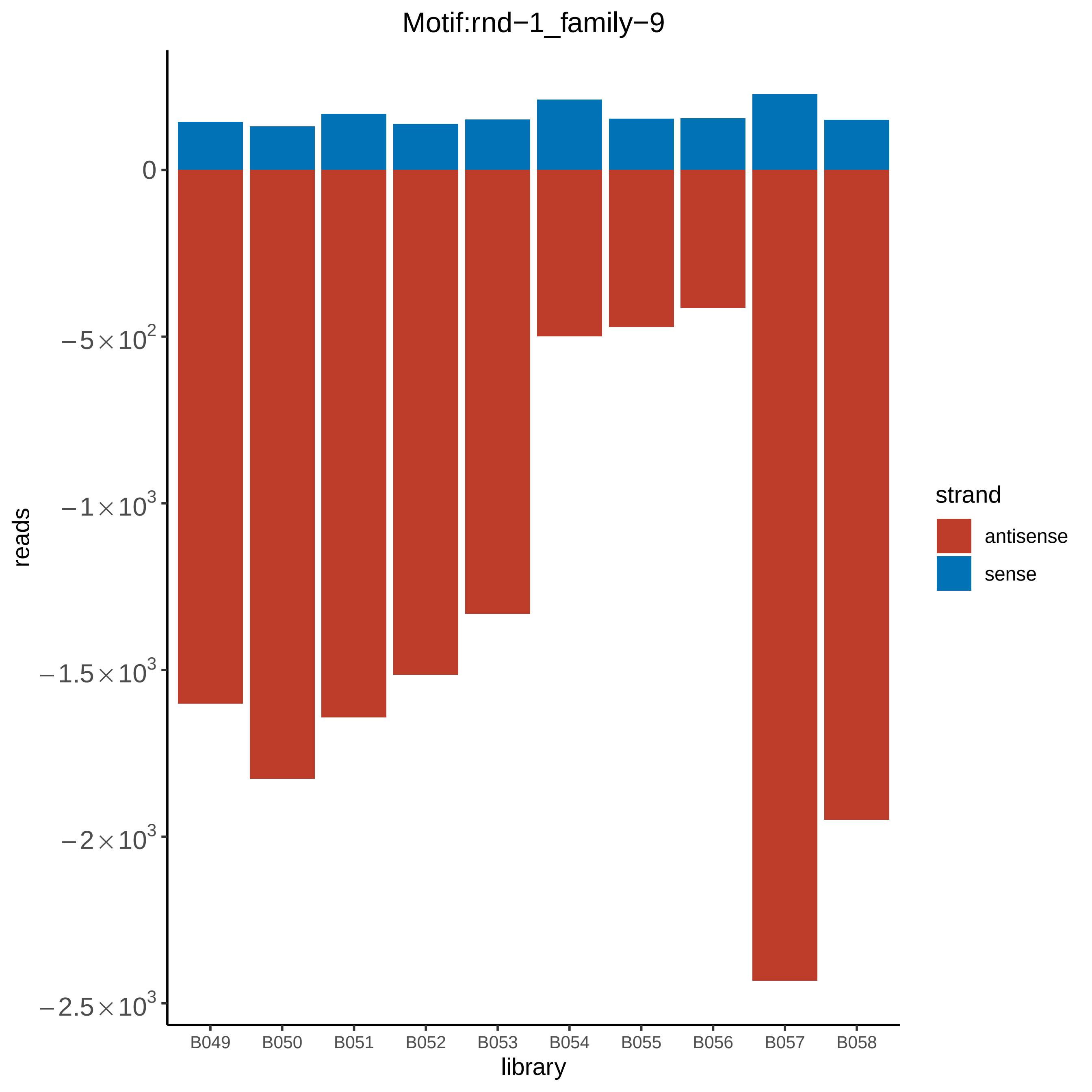

Motif:rnd−1_family−9
0
−5×10^2
−1×10^3
−1.5×10^3
−2×10^3
−2.5×10^3
reads
B049
B050
B051
B052
B053
B054
B055
B056
B057
B058
library
strand
antisense
sense

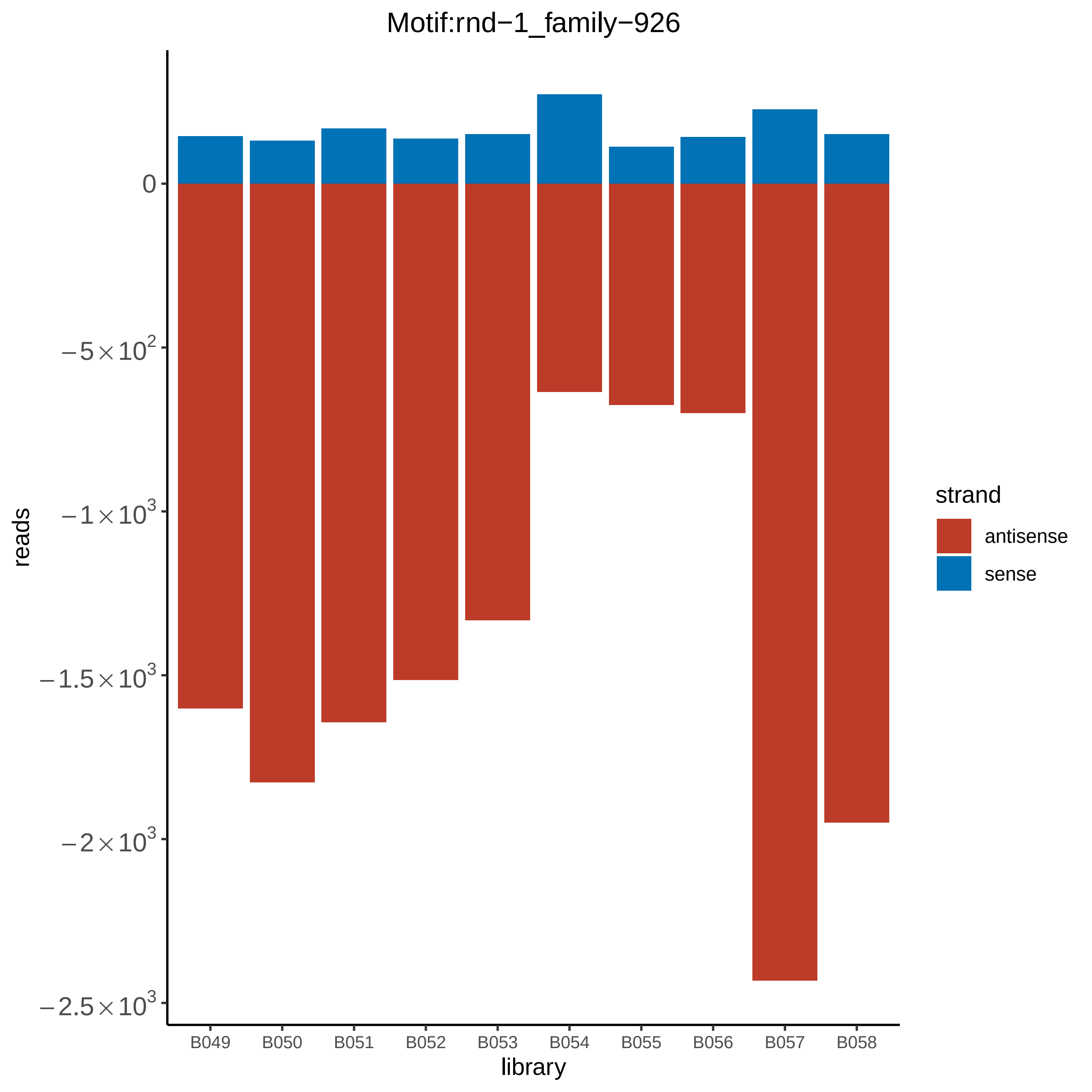

Motif:rnd−1_family−926
0
−5×10^2
−1×10^3
−1.5×10^3
−2×10^3
−2.5×10^3
reads
B049
B050
B051
B052
B053
B054
B055
B056
B057
B058
library
strand
antisense
sense

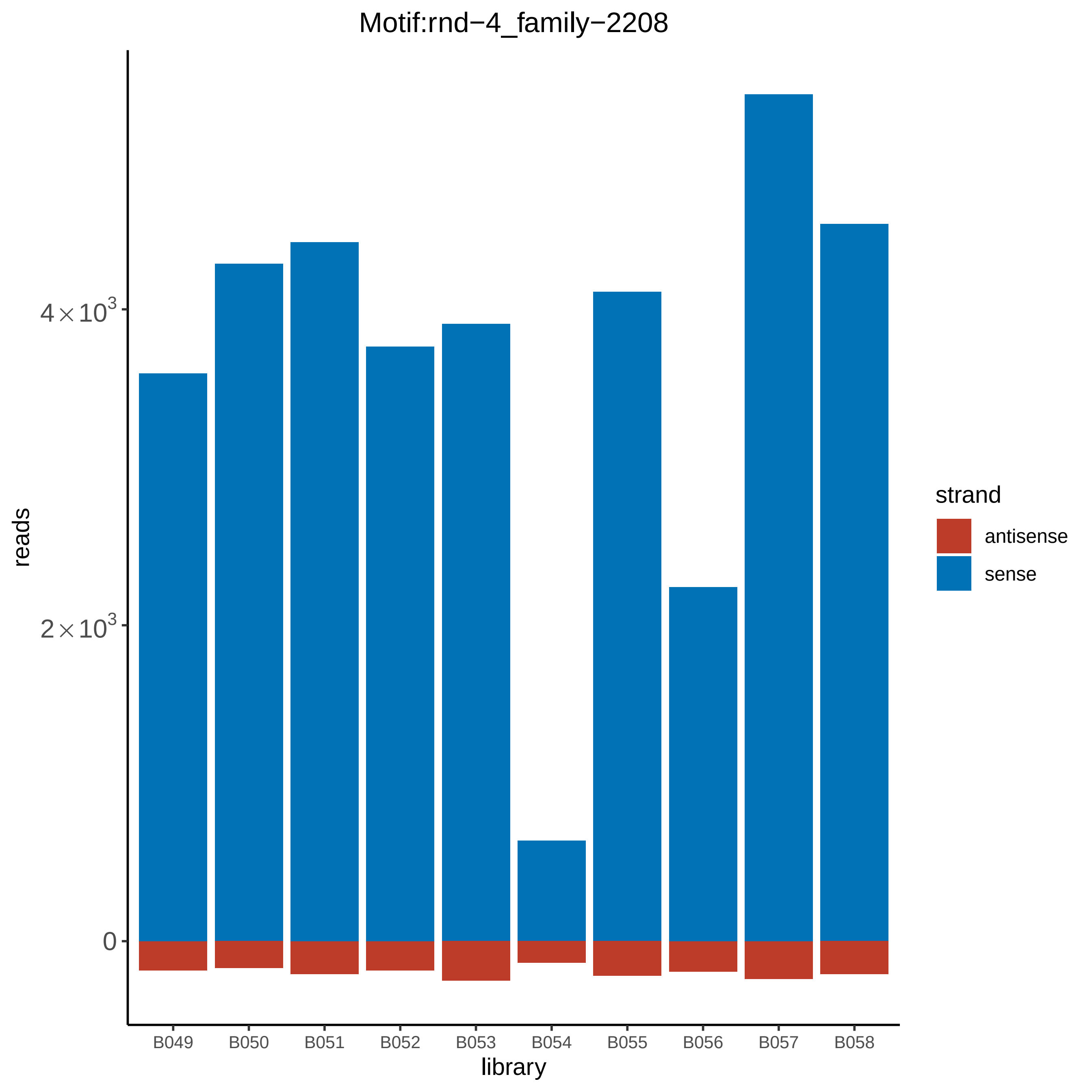

Motif:rnd−4_family−2208
reads
4×10^3
2×10^3
0
B049
B050
B051
B052
B053
B054
B055
B056
B057
B058
library
strand
antisense
sense

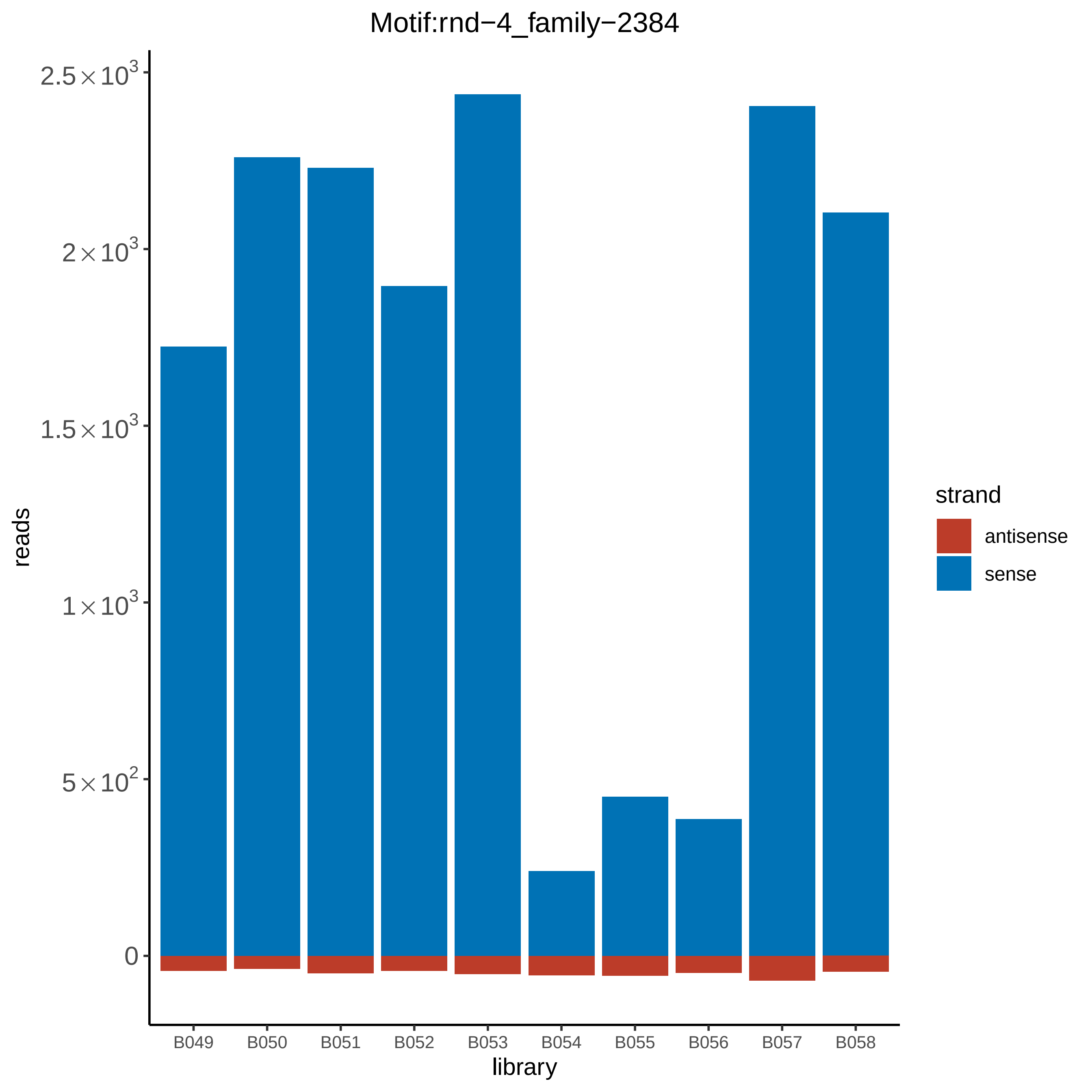

Motif:rnd−4_family−2384
reads
2.5×10^3
2×10^3
1.5×10^3
1×10^3
5×10^2
0
B049
B050
B051
B052
B053
B054
B055
B056
B057
B058
library
strand
antisense
sense

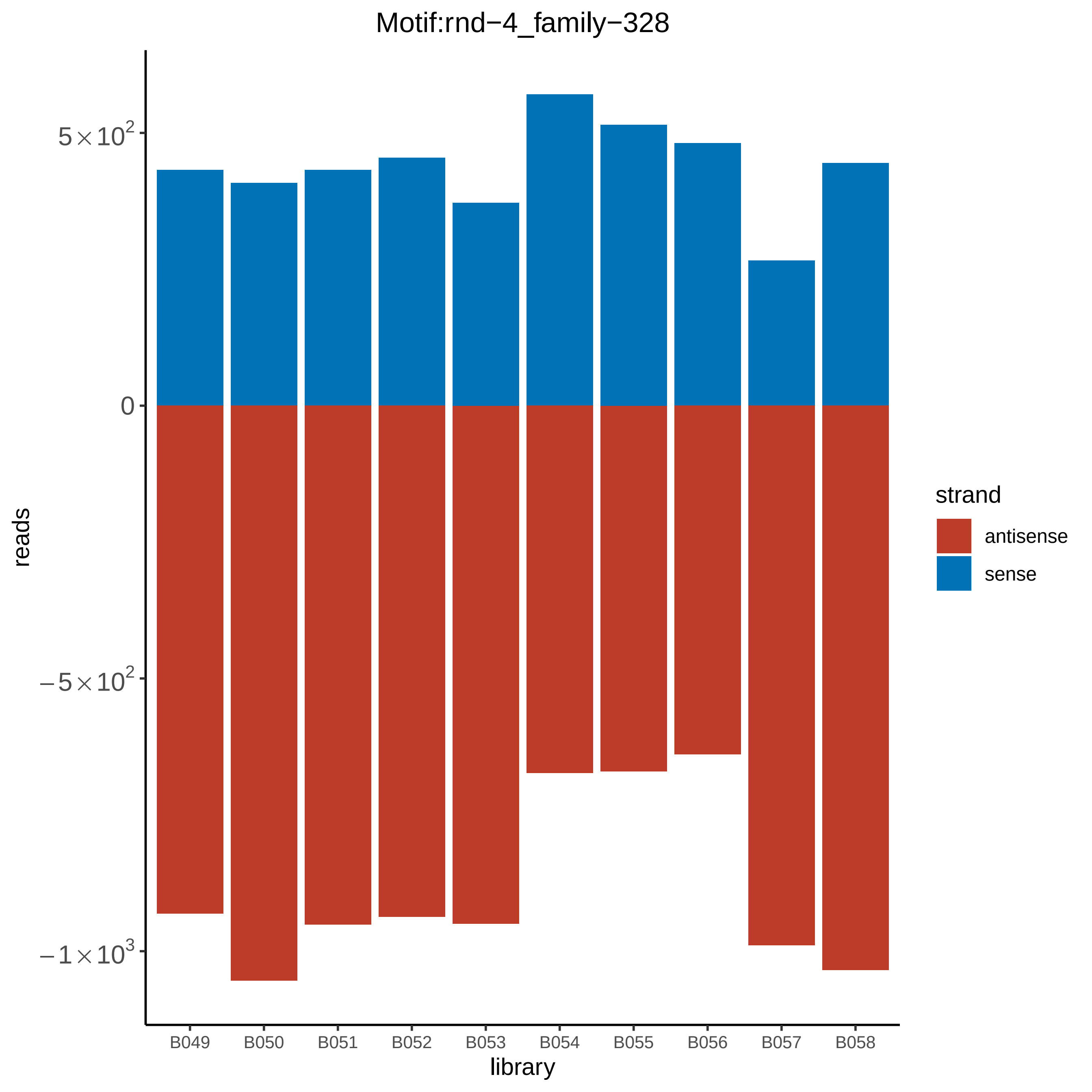

Motif:rnd−4_family−328
reads
5×10^2
0
−5×10^2
−1×10^3
library
B049
B050
B051
B052
B053
B054
B055
B056
B057
B058
strand
antisense
sense

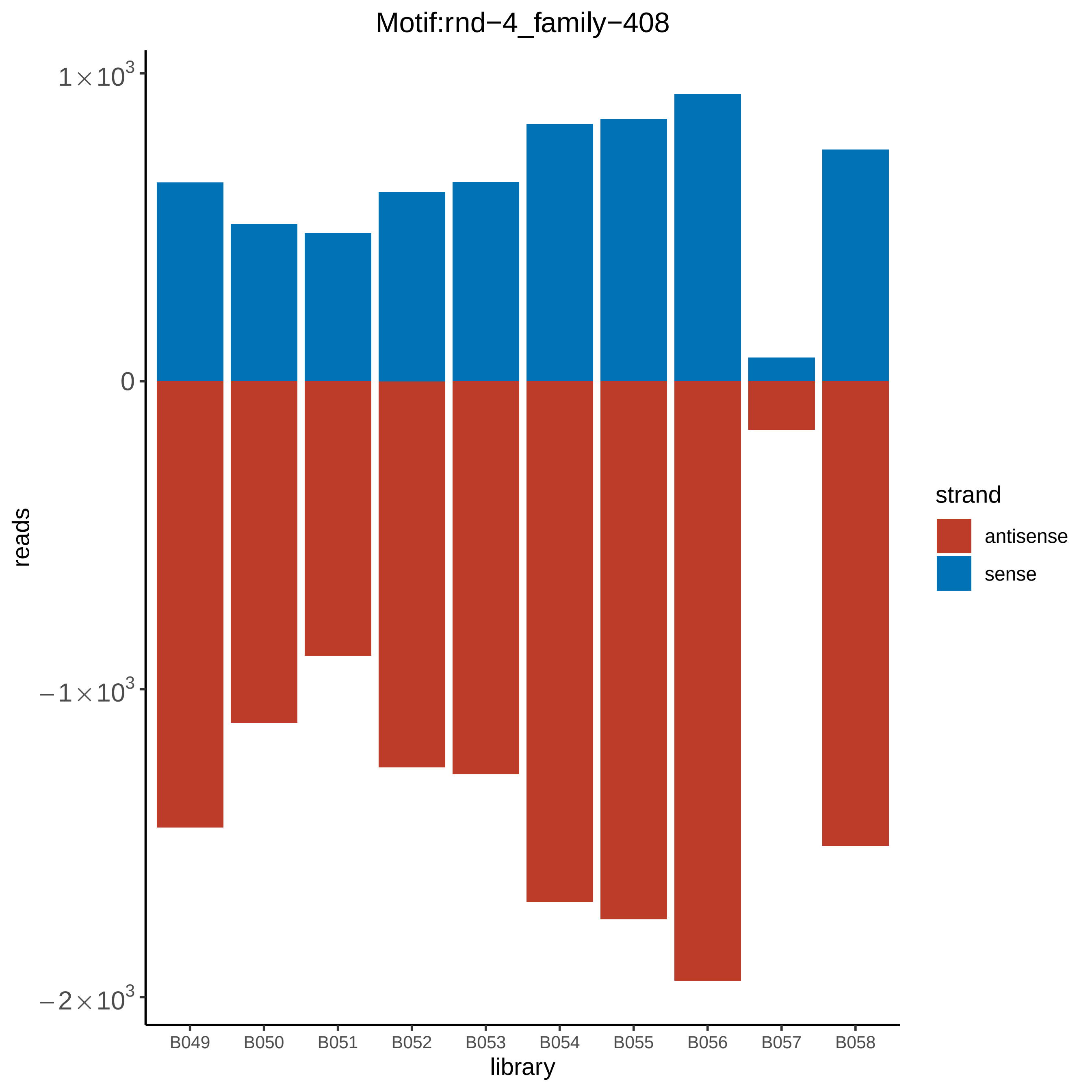

Motif:rnd−4_family−408
reads
1×10^3
0
-1×10^3
-2×10^3
B049
B050
B051
B052
B053
B054
B055
B056
B057
B058
library
strand
antisense
sense

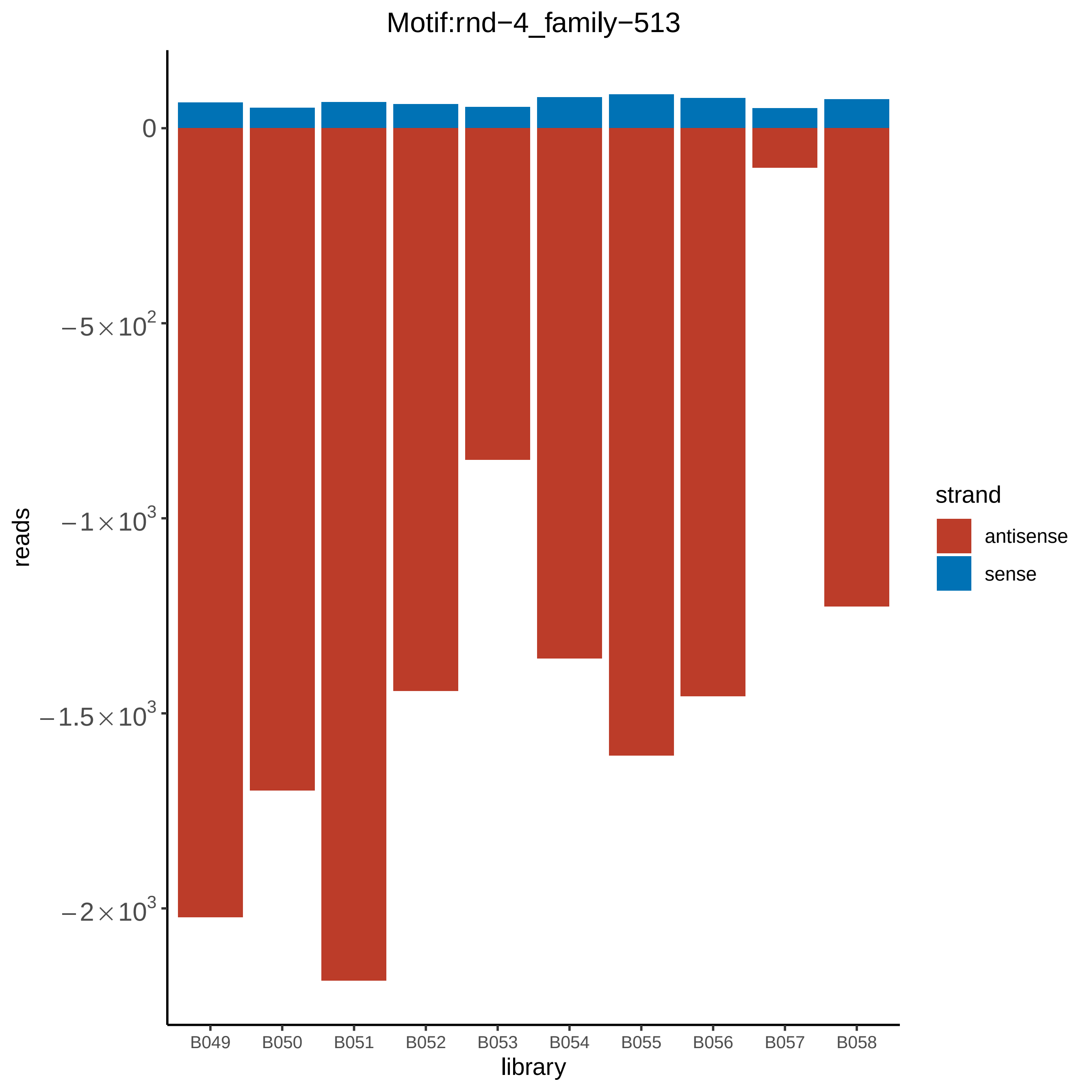

Motif:rnd−4_family−513
0
−5×10^2
−1×10^3
−1.5×10^3
−2×10^3
reads
B049
B050
B051
B052
B053
B054
B055
B056
B057
B058
library
strand
antisense
sense

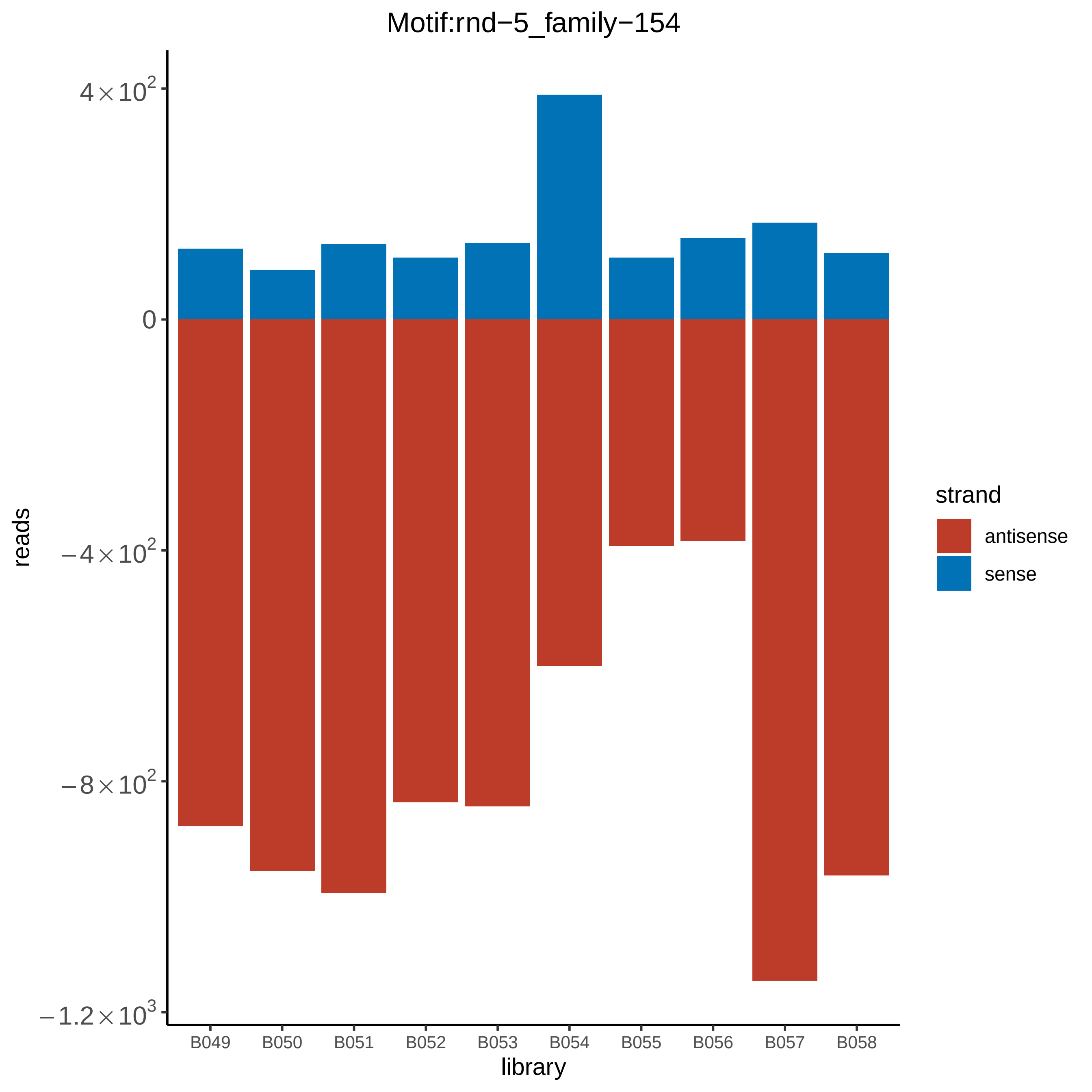

Motif:rnd−5_family−154
4×10^2
0
−4×10^2
−8×10^2
−1.2×10^3
reads
B049
B050
B051
B052
B053
B054
B055
B056
B057
B058
library
strand
antisense
sense

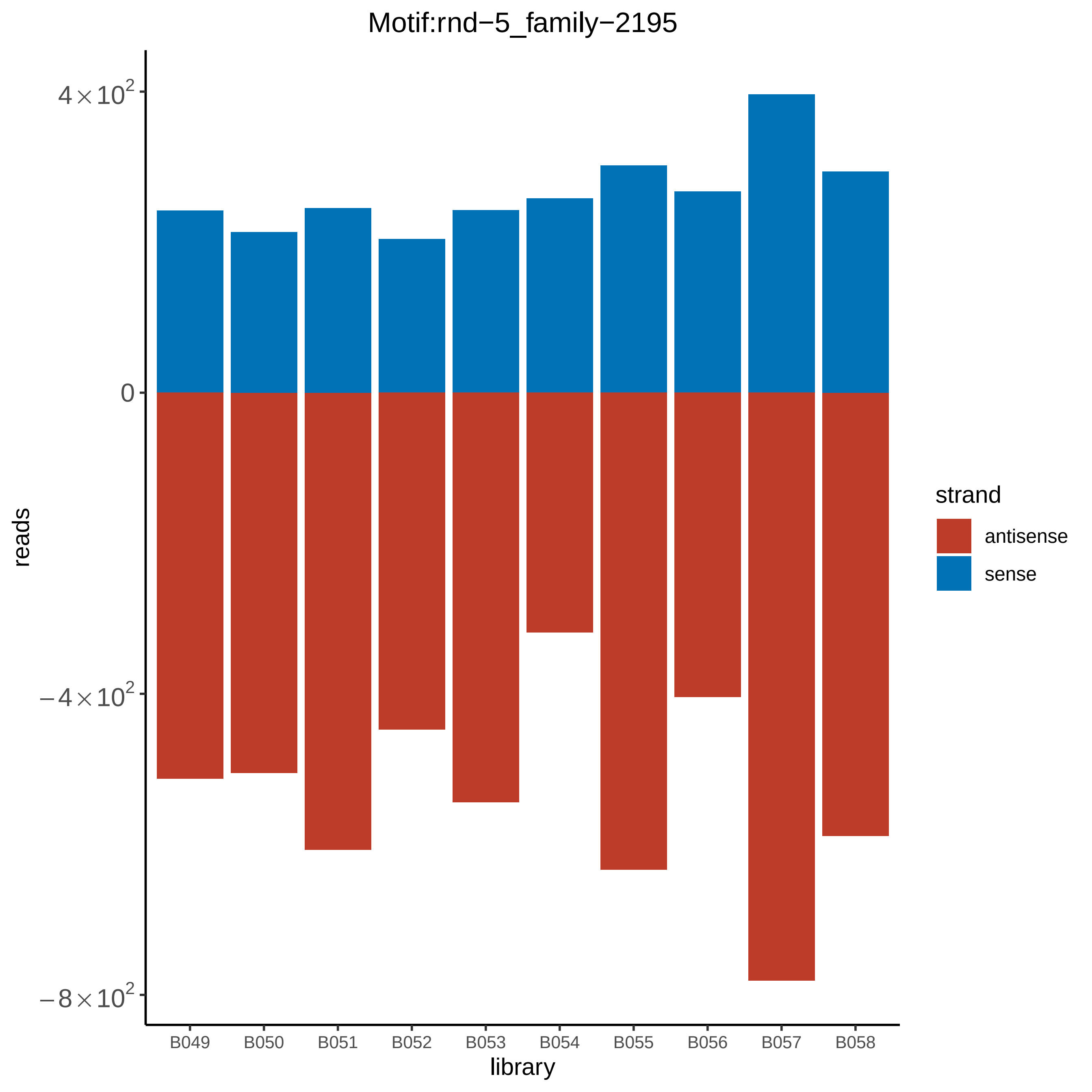

Motif:rnd−5_family−2195
4×10^2
0
−4×10^2
−8×10^2
reads
B049
B050
B051
B052
B053
B054
B055
B056
B057
B058
library
strand
antisense
sense

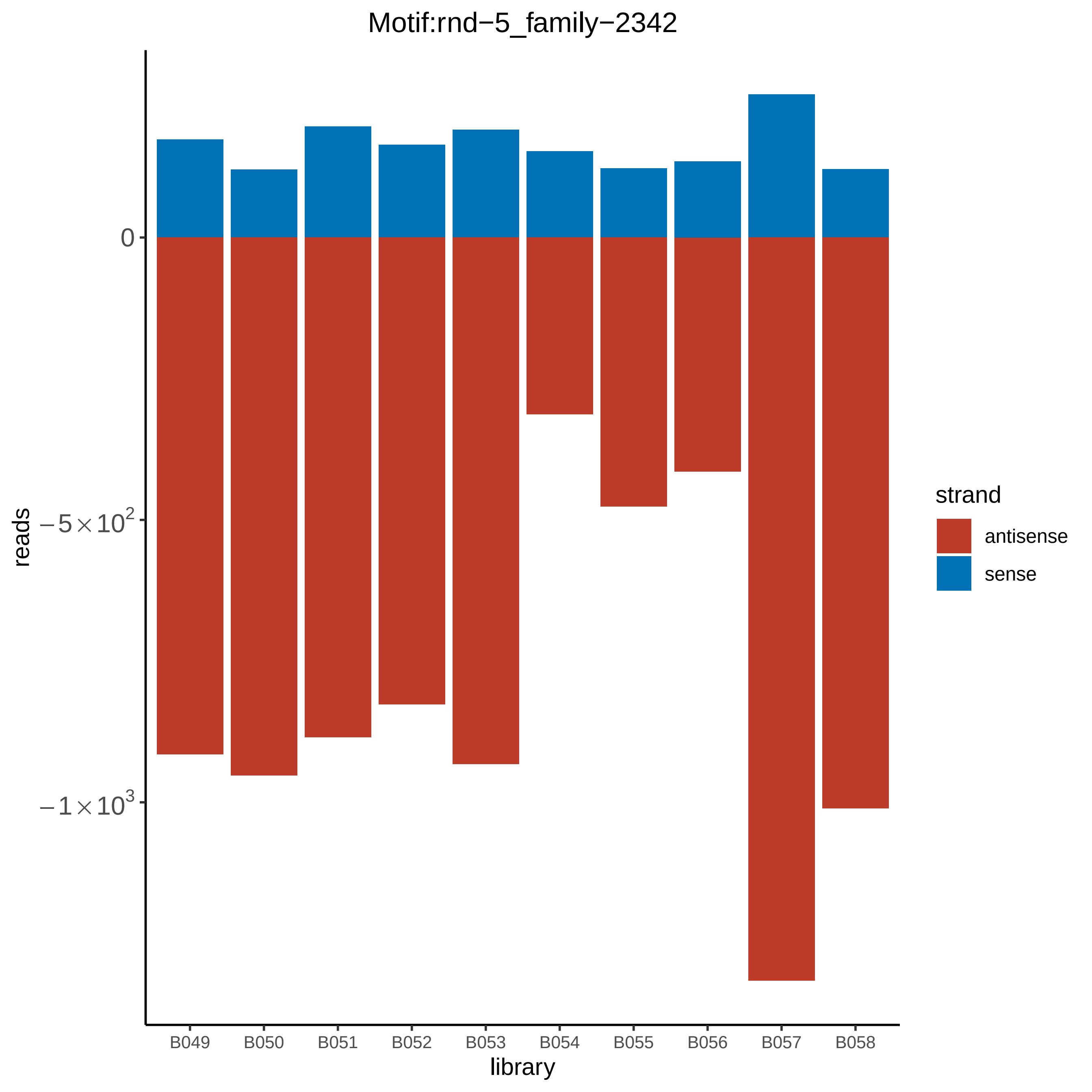

Motif:rnd−5_family−2342
0
−5×10^2
−1×10^3
reads
B049
B050
B051
B052
B053
B054
B055
B056
B057
B058
library
strand
antisense
sense

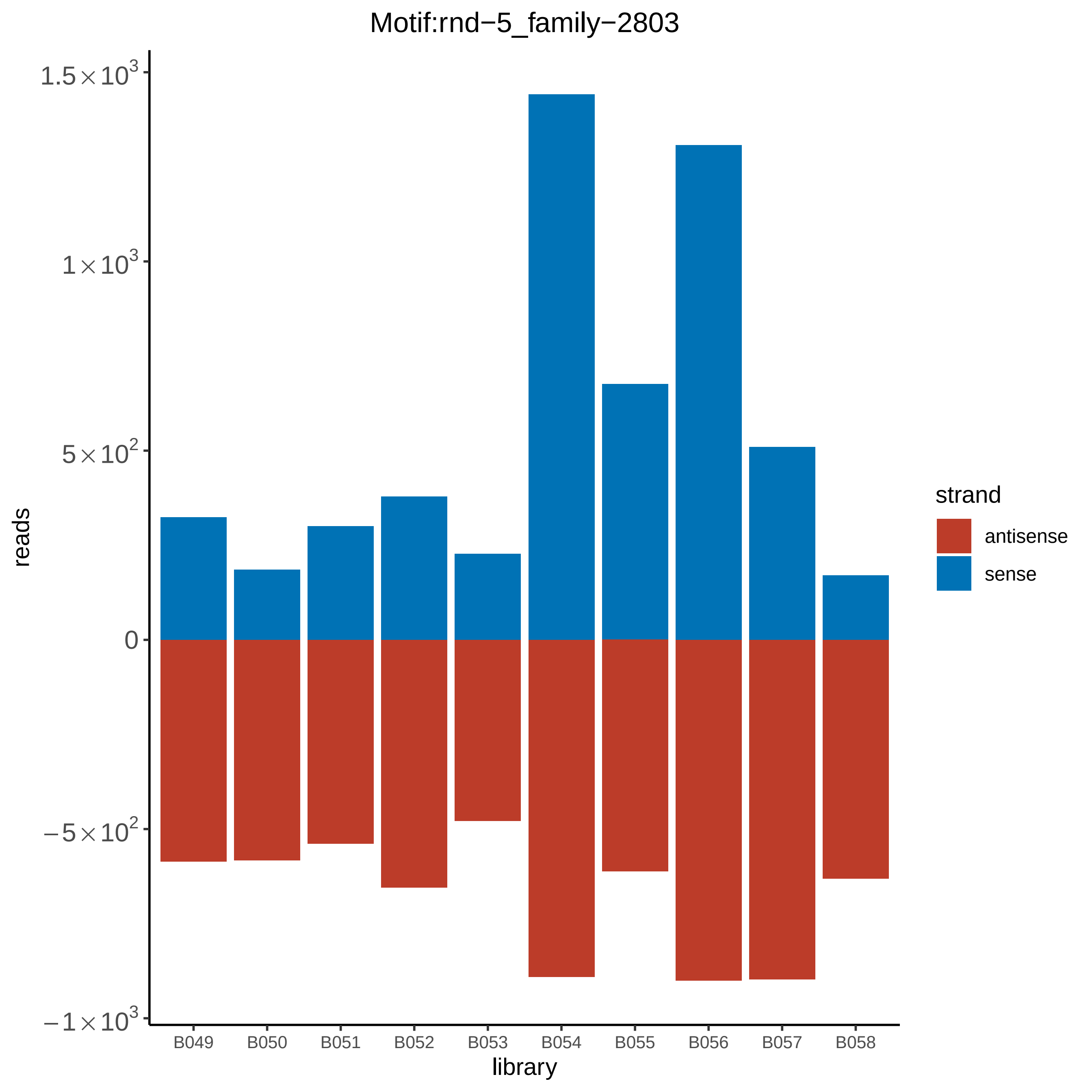

Motif:rnd−5_family−2803
reads
1.5×10^3
1×10^3
5×10^2
0
-5×10^2
-1×10^3
B049
B050
B051
B052
B053
B054
B055
B056
B057
B058
library
strand
antisense
sense

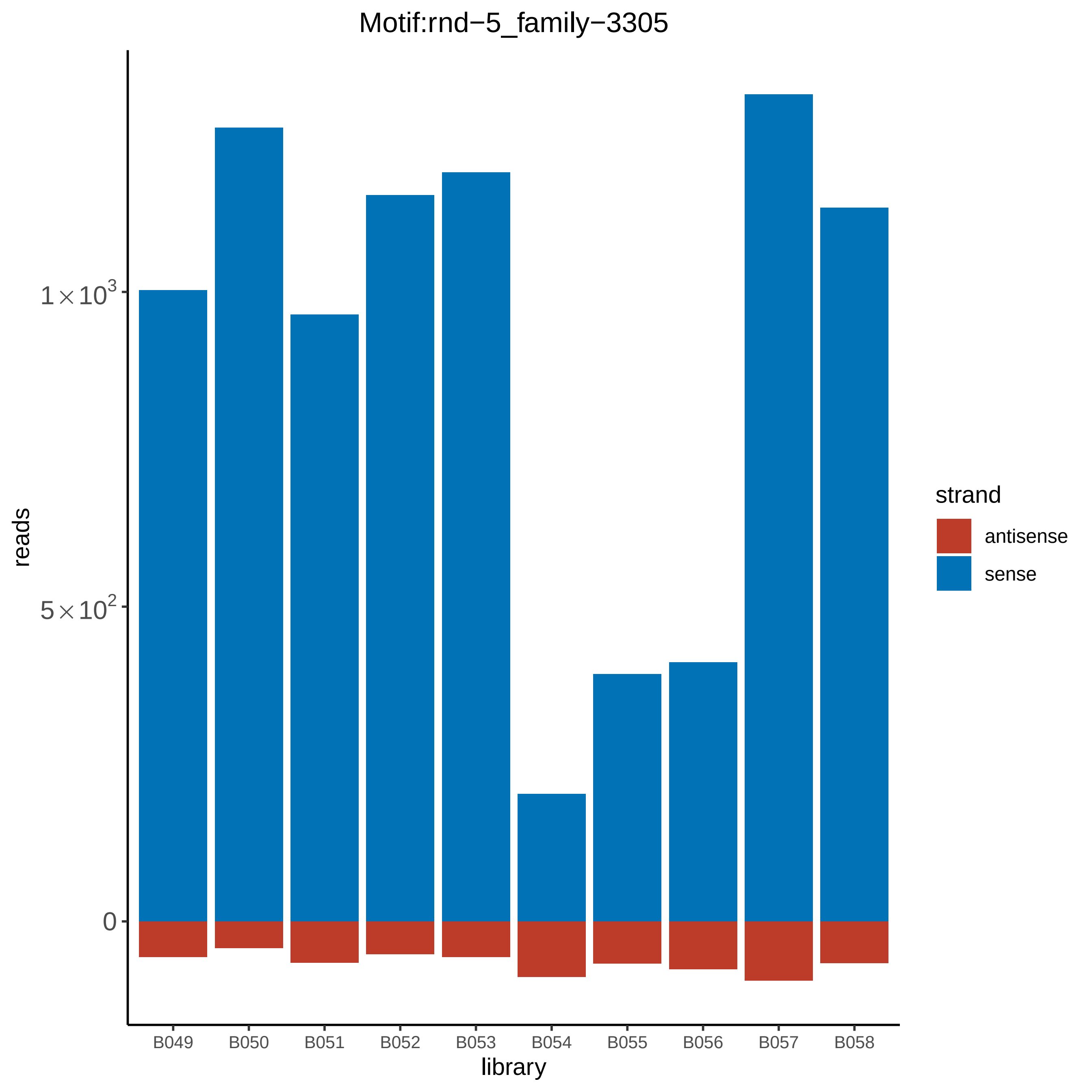
Motif:rnd−5_family−3305
reads
1×10^3
5×10^2
0
B049
B050
B051
B052
B053
B054
B055
B056
B057
B058
library
strand
antisense
sense

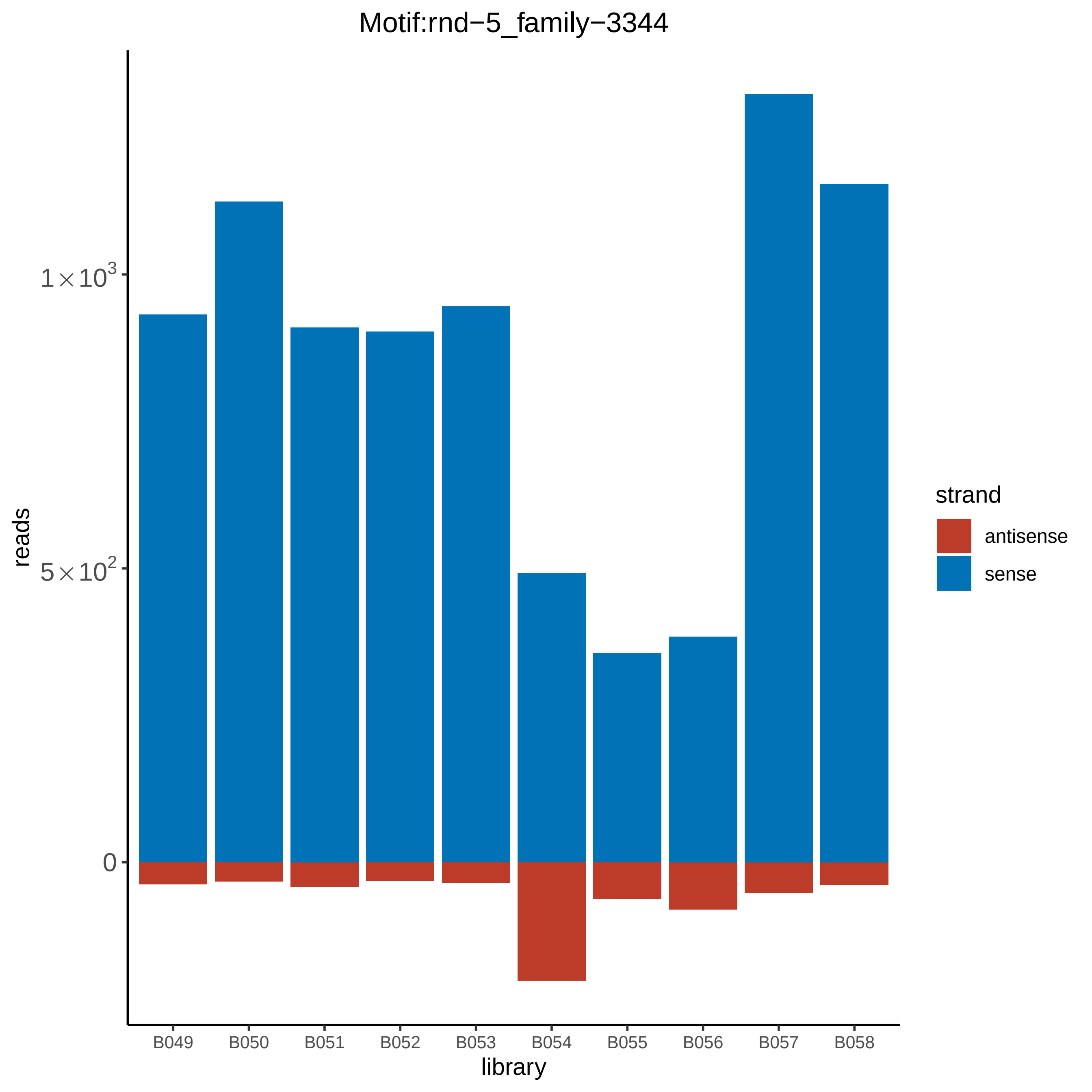

Motif:rnd−5_family−3344
reads
1×10^3
5×10^2
0
B049
B050
B051
B052
B053
B054
B055
B056
B057
B058
library
strand
antisense
sense

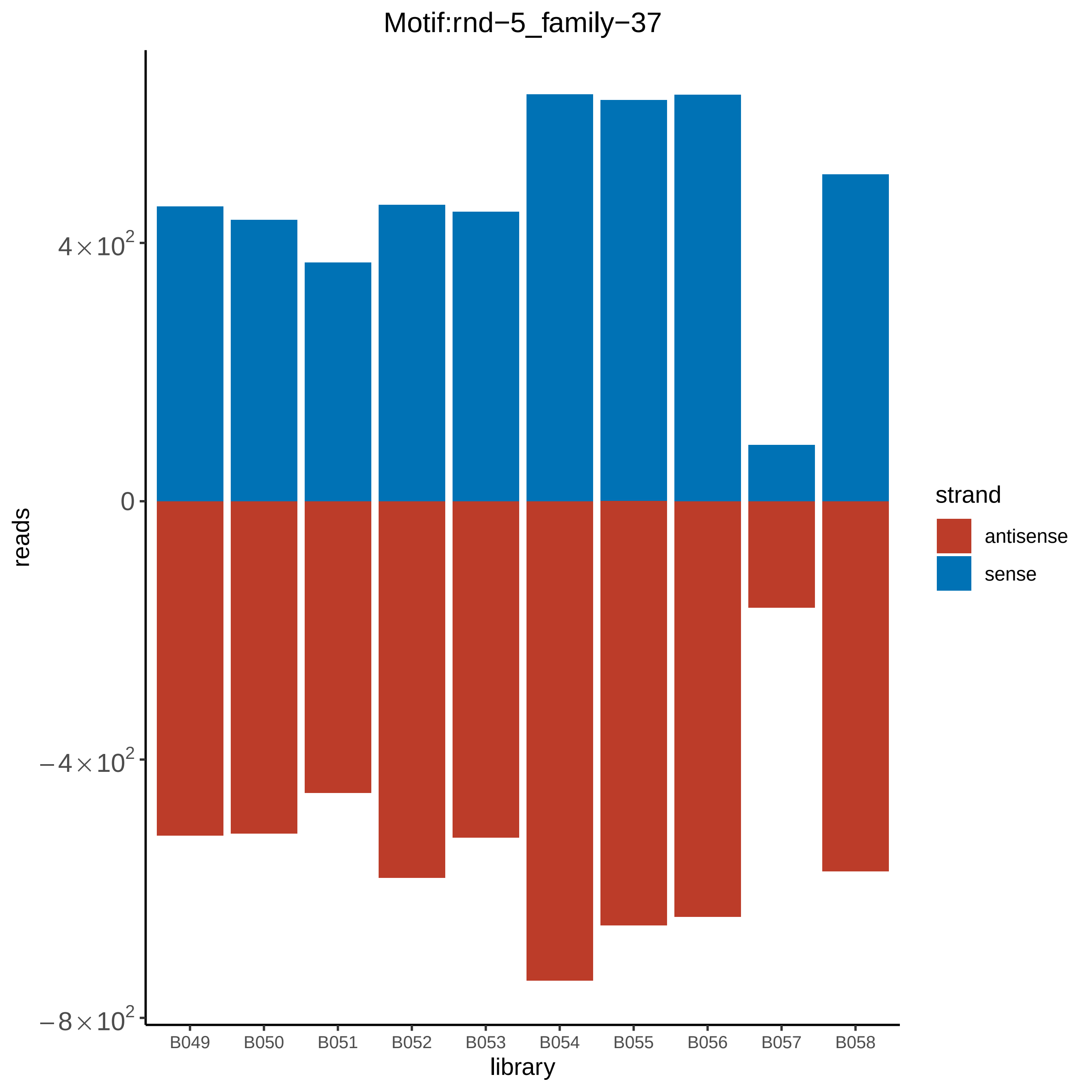

Motif:rnd−5_family−37
reads
4×10^2
0
−4×10^2
−8×10^2
library
B049
B050
B051
B052
B053
B054
B055
B056
B057
B058
strand
antisense
sense

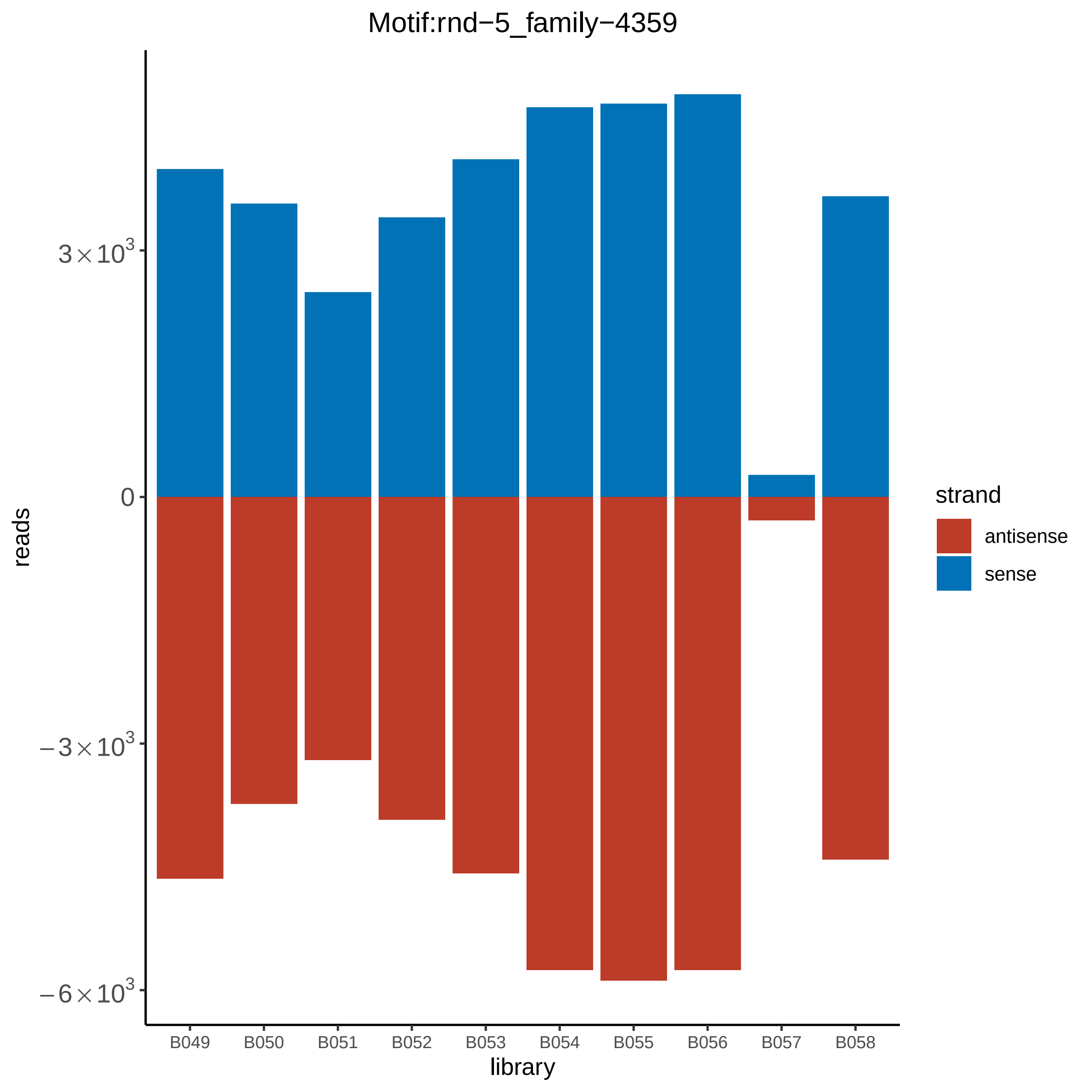

Motif:rnd−5_family−4359
3×10^3
0
-3×10^3
-6×10^3
reads
B049
B050
B051
B052
B053
B054
B055
B056
B057
B058
library
strand
antisense
sense

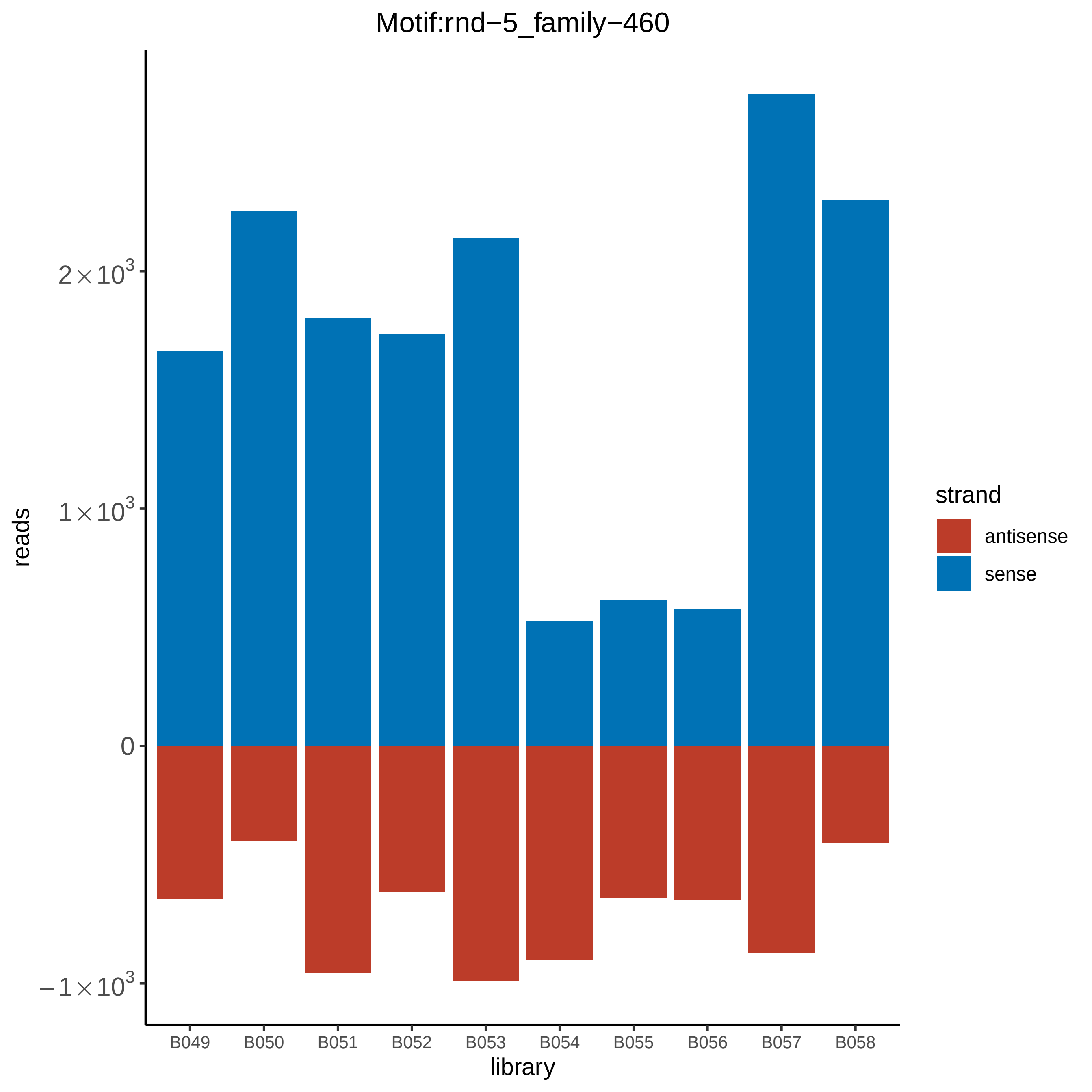
Motif:rnd−5_family−460
reads
2×10^3
1×10^3
0
-1×10^3
B049
B050
B051
B052
B053
B054
B055
B056
B057
B058
library
strand
antisense
sense

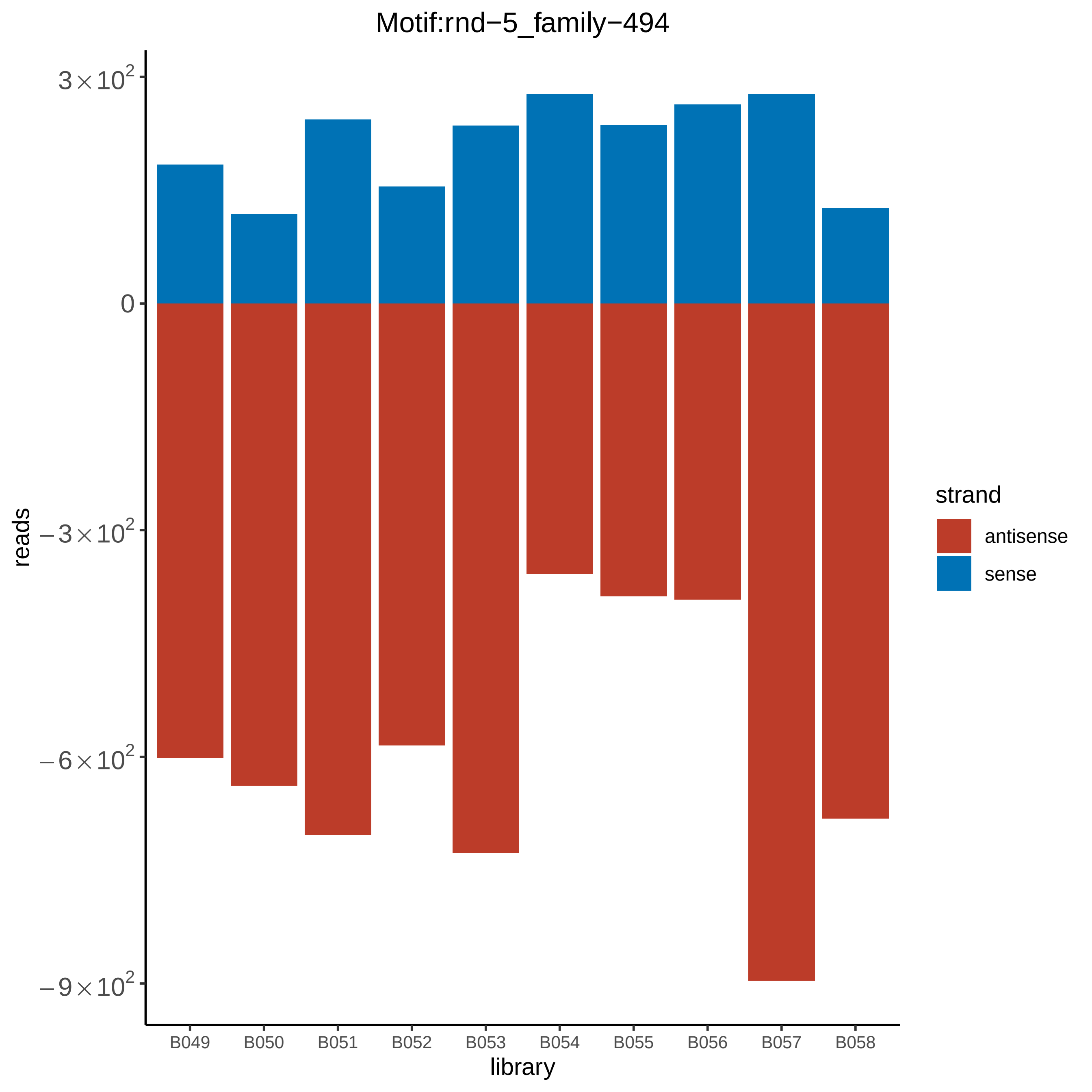

Motif:rnd−5_family−494
3×10^2
0
-3×10^2
-6×10^2
-9×10^2
reads
B049
B050
B051
B052
B053
B054
B055
B056
B057
B058
library
strand
antisense
sense

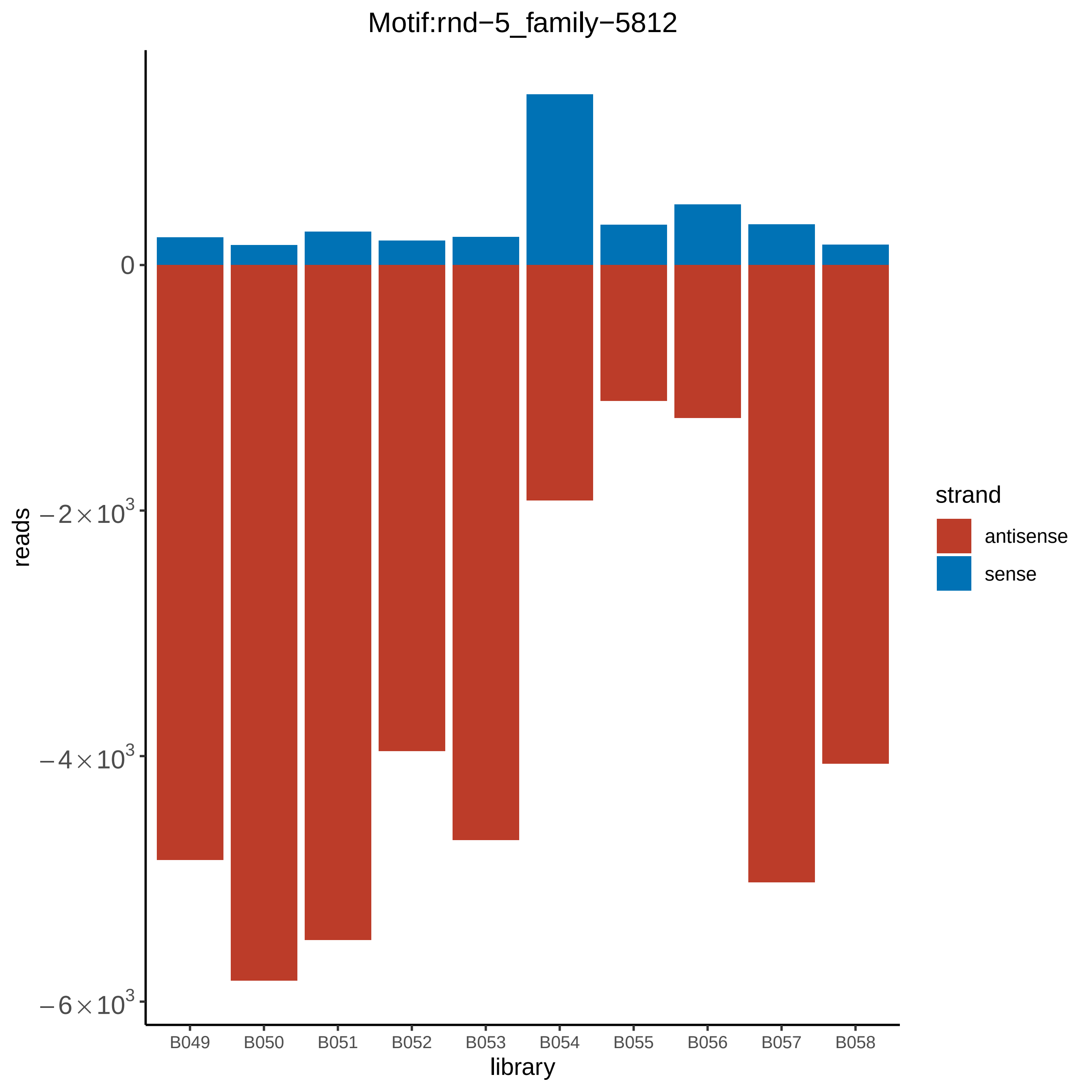

Motif:rnd−5_family−5812
0
-2×10^3
-4×10^3
-6×10^3
reads
B049
B050
B051
B052
B053
B054
B055
B056
B057
B058
library
strand
antisense
sense

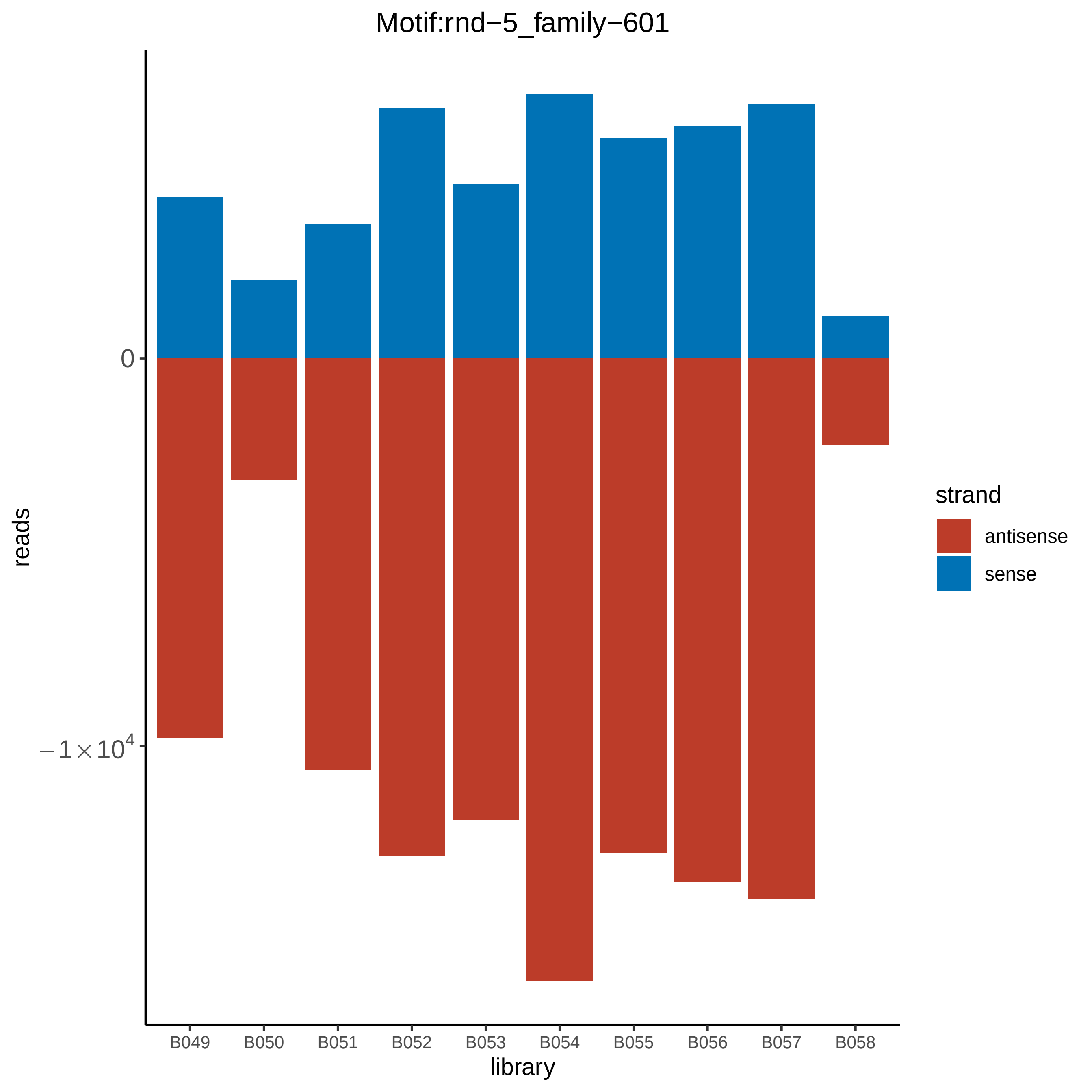

Motif:rnd−5_family−601
reads
0
−1 × 10^4
library
B049
B050
B051
B052
B053
B054
B055
B056
B057
B058
strand
antisense
sense

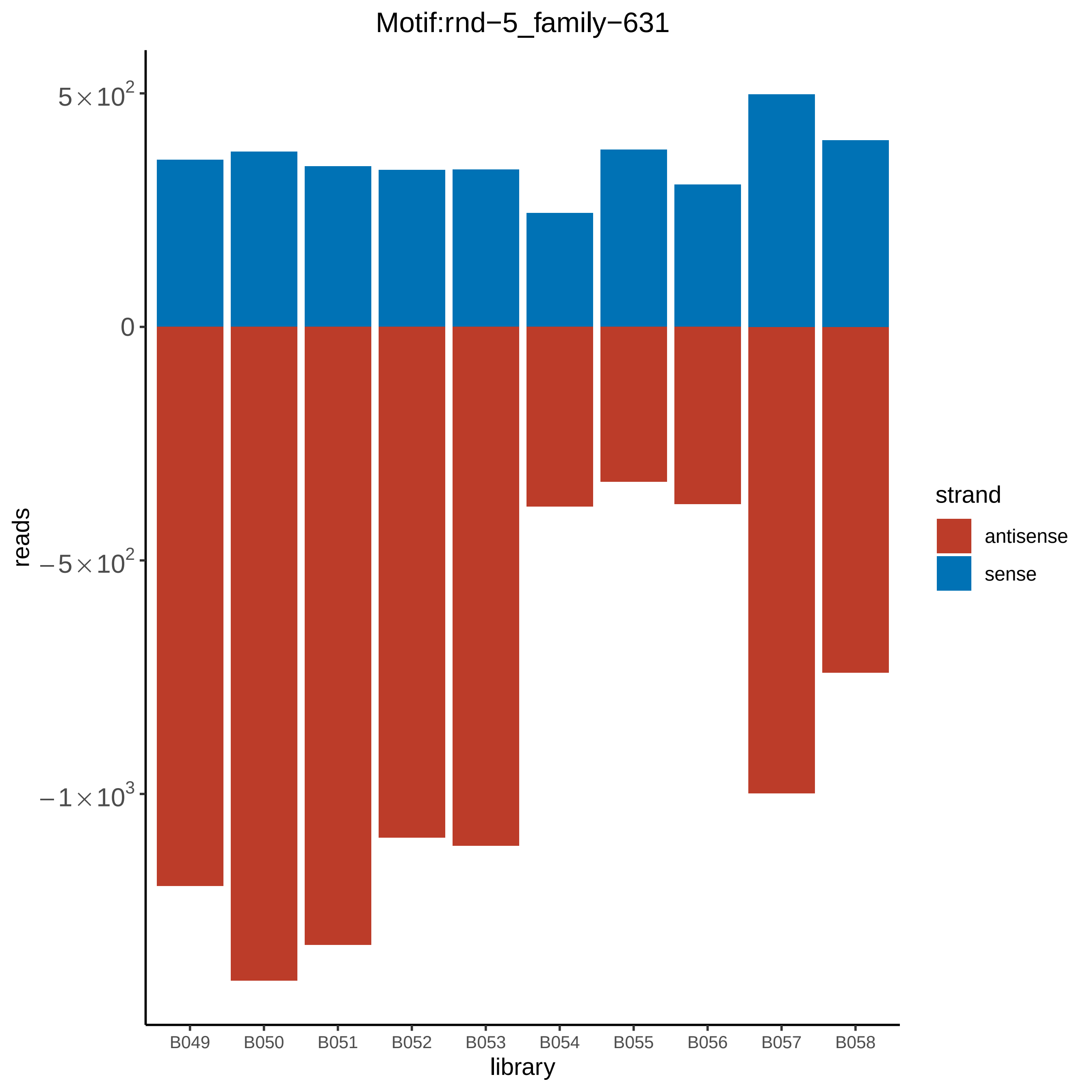

Motif:rnd−5_family−631
5×10^2
0
-5×10^2
-1×10^3
reads
B049
B050
B051
B052
B053
B054
B055
B056
B057
B058
library
strand
antisense
sense

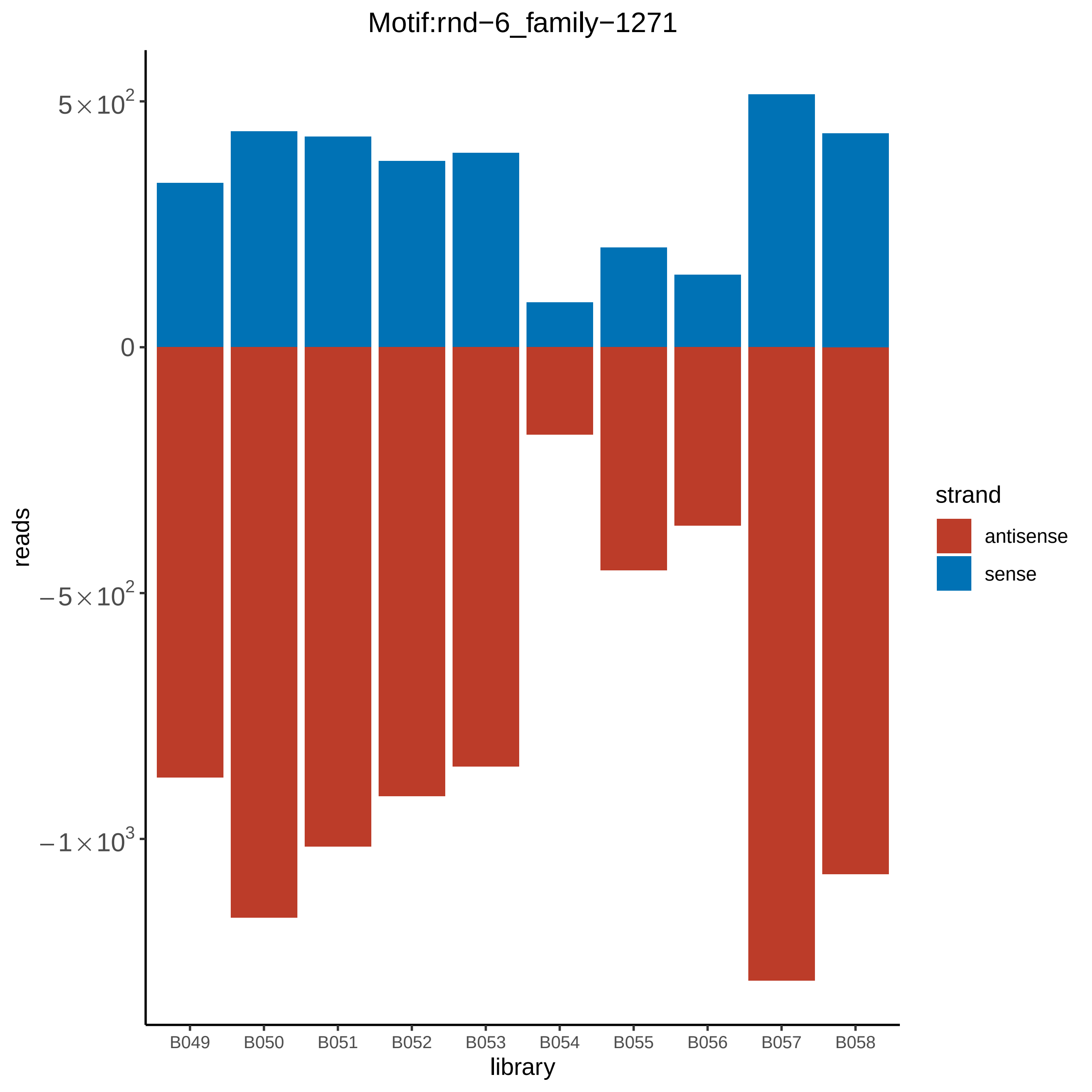

Motif:rnd−6_family−1271
5×10^2
0
-5×10^2
-1×10^3
reads
B049
B050
B051
B052
B053
B054
B055
B056
B057
B058
library
strand
antisense
sense

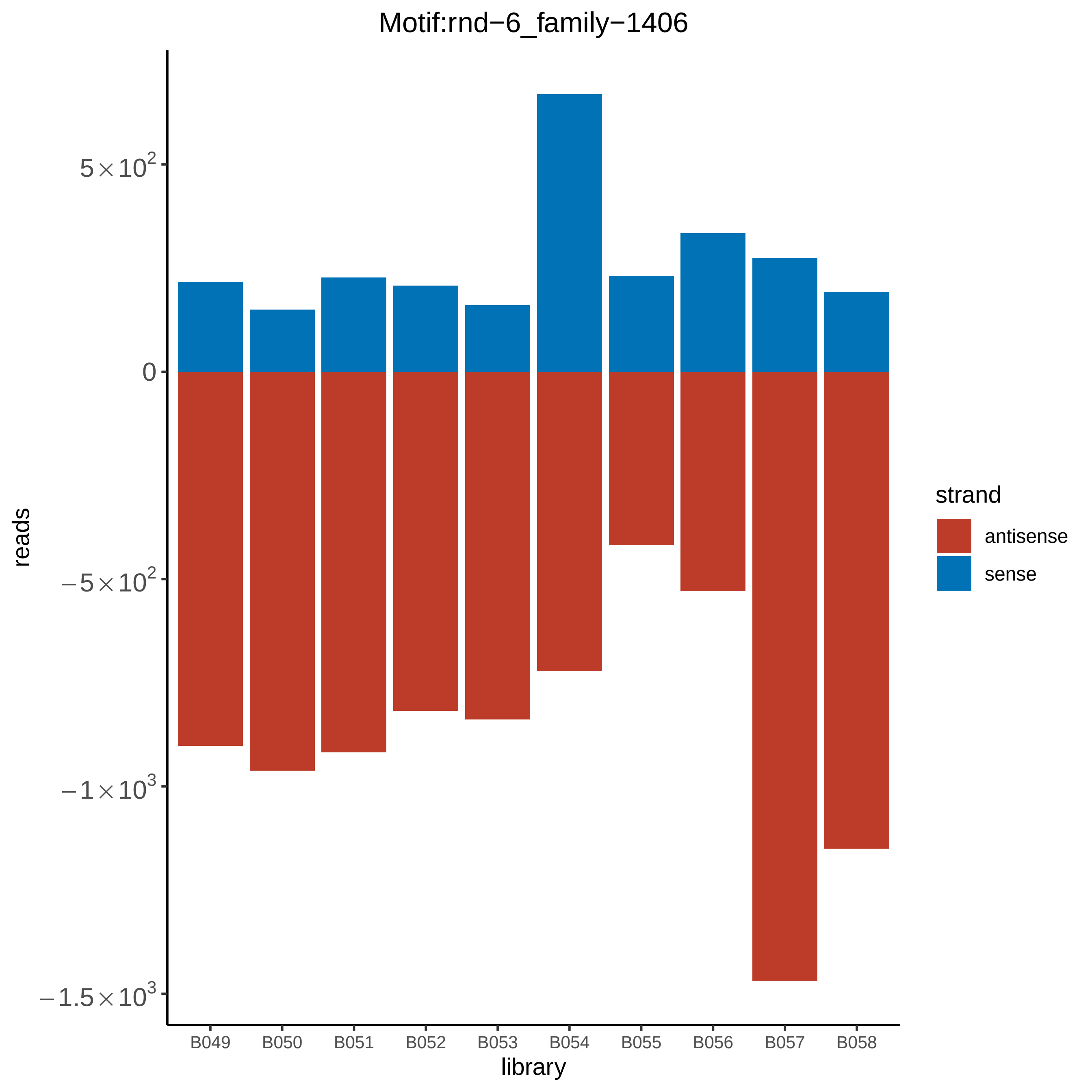

Motif:rnd−6_family−1406
reads
5×10^2
0
-5×10^2
-1×10^3
-1.5×10^3
B049
B050
B051
B052
B053
B054
B055
B056
B057
B058
library
strand
antisense
sense

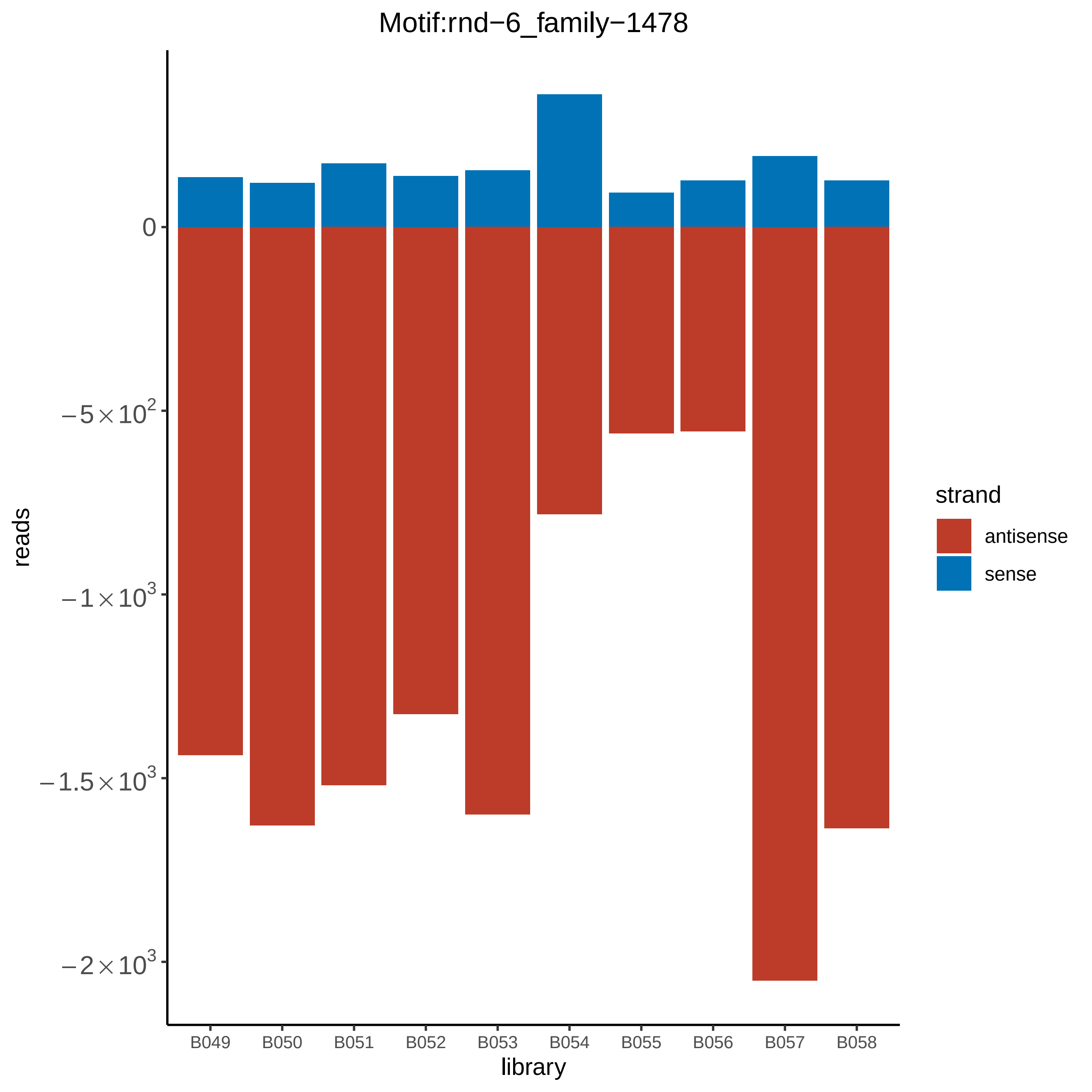

Motif:rnd−6_family−1478
0
−5×10^2
−1×10^3
−1.5×10^3
−2×10^3
reads
B049
B050
B051
B052
B053
B054
B055
B056
B057
B058
library
strand
antisense
sense

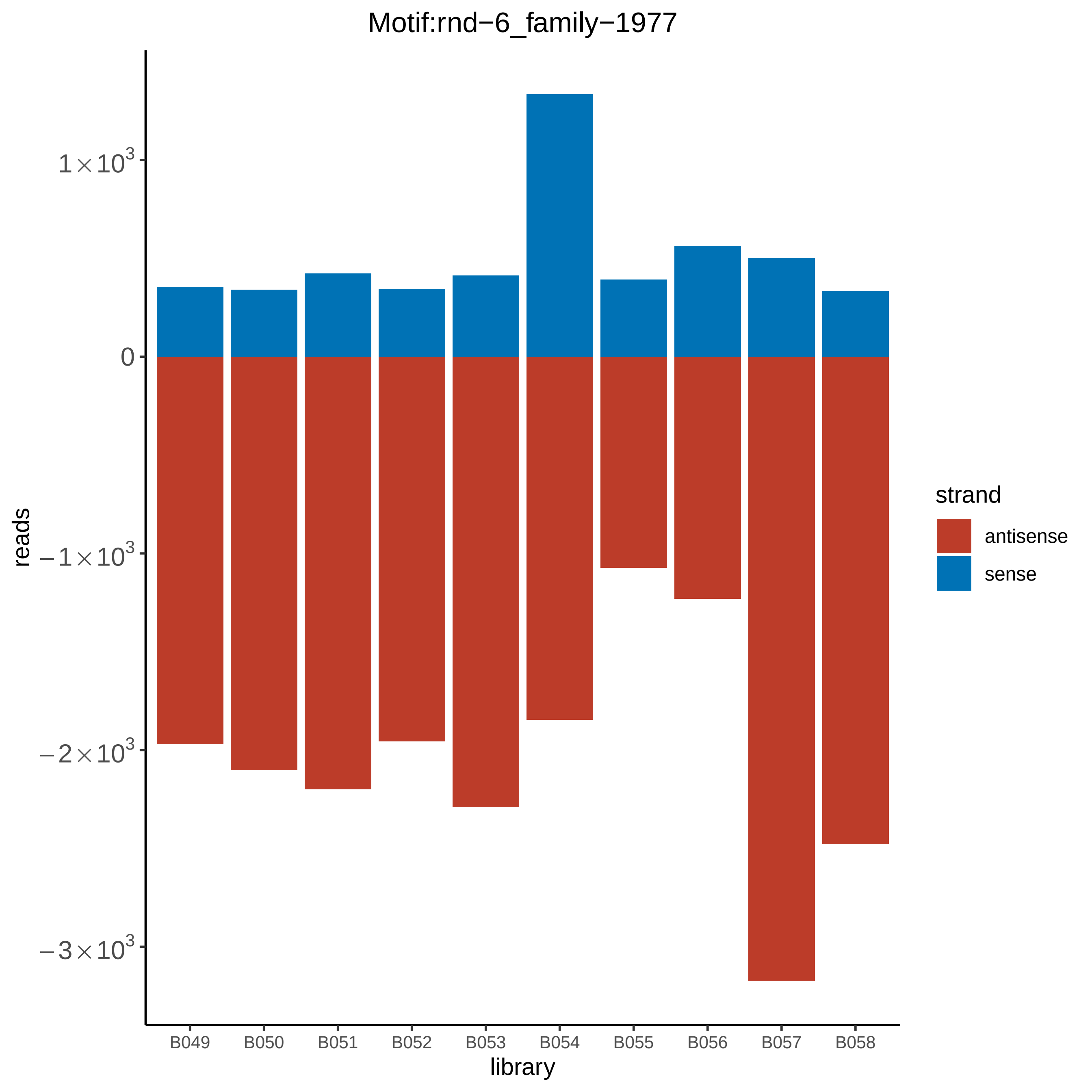

Motif:rnd−6_family−1977
1×10^3
0
−1×10^3
−2×10^3
−3×10^3
reads
B049
B050
B051
B052
B053
B054
B055
B056
B057
B058
library
strand
antisense
sense

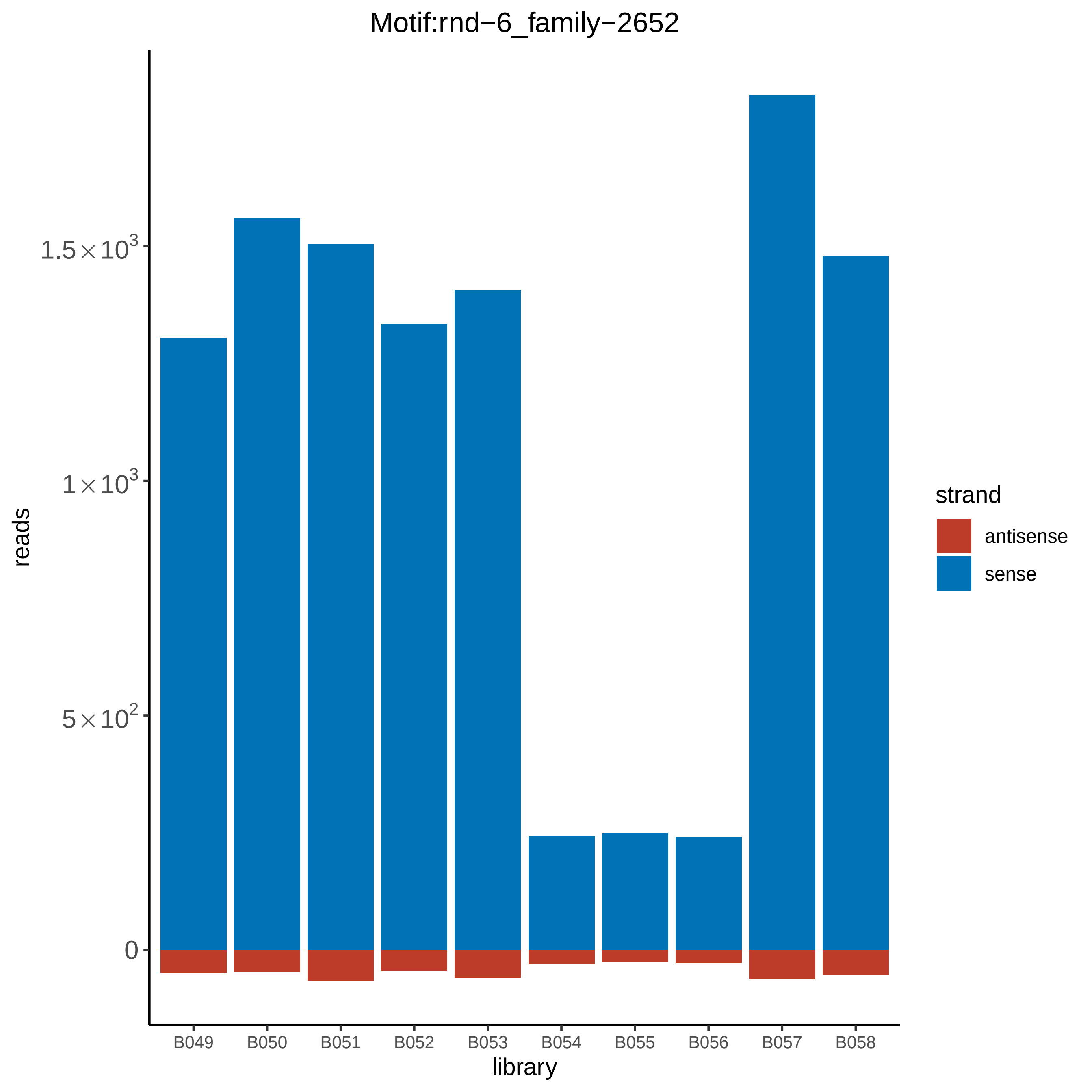

Motif:rnd−6_family−2652
reads
1.5×10³
1×10³
5×10²
0
B049
B050
B051
B052
B053
B054
B055
B056
B057
B058
library
strand
antisense
sense

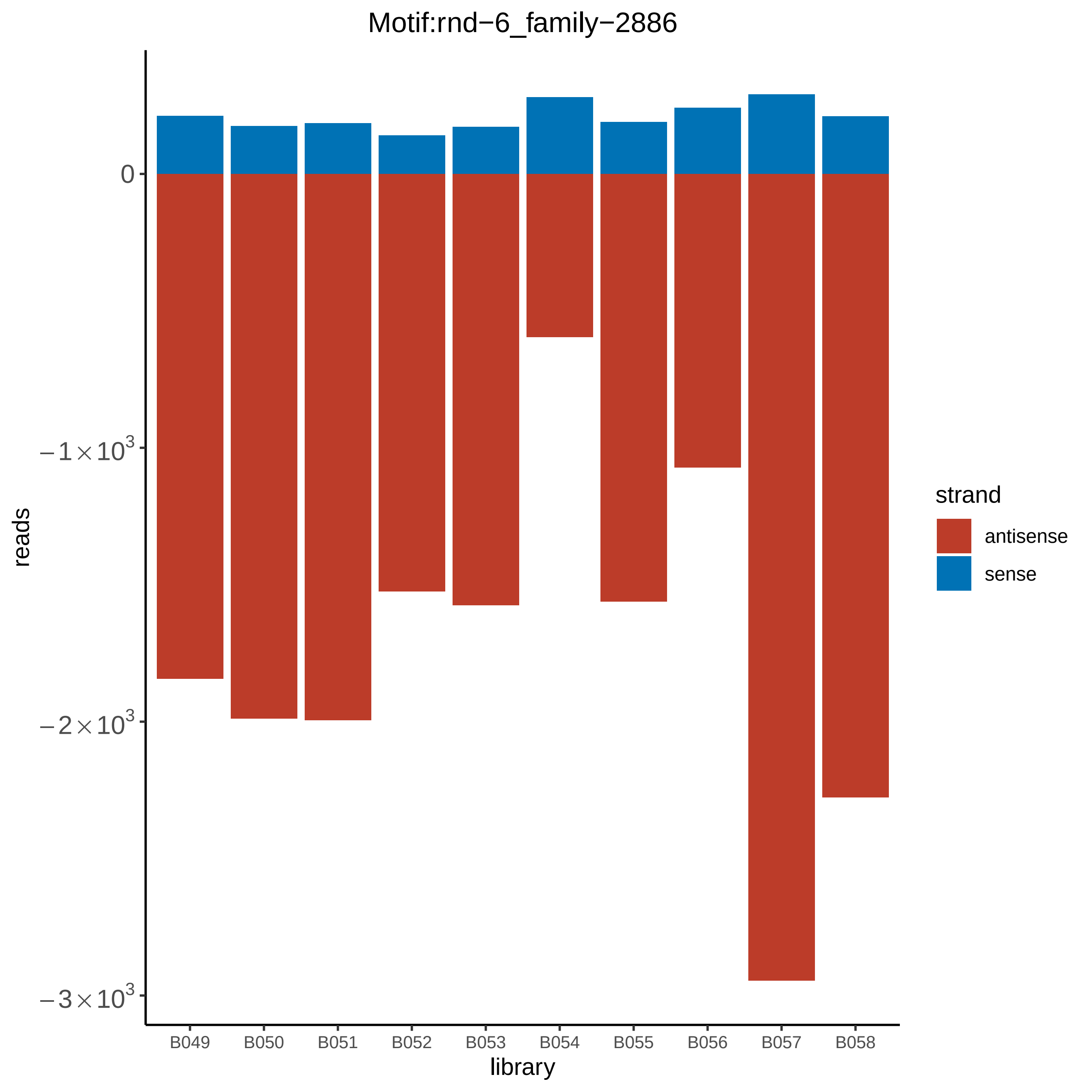

Motif:rnd−6_family−2886
0
−1×10^3
−2×10^3
−3×10^3
reads
B049
B050
B051
B052
B053
B054
B055
B056
B057
B058
library
strand
antisense
sense

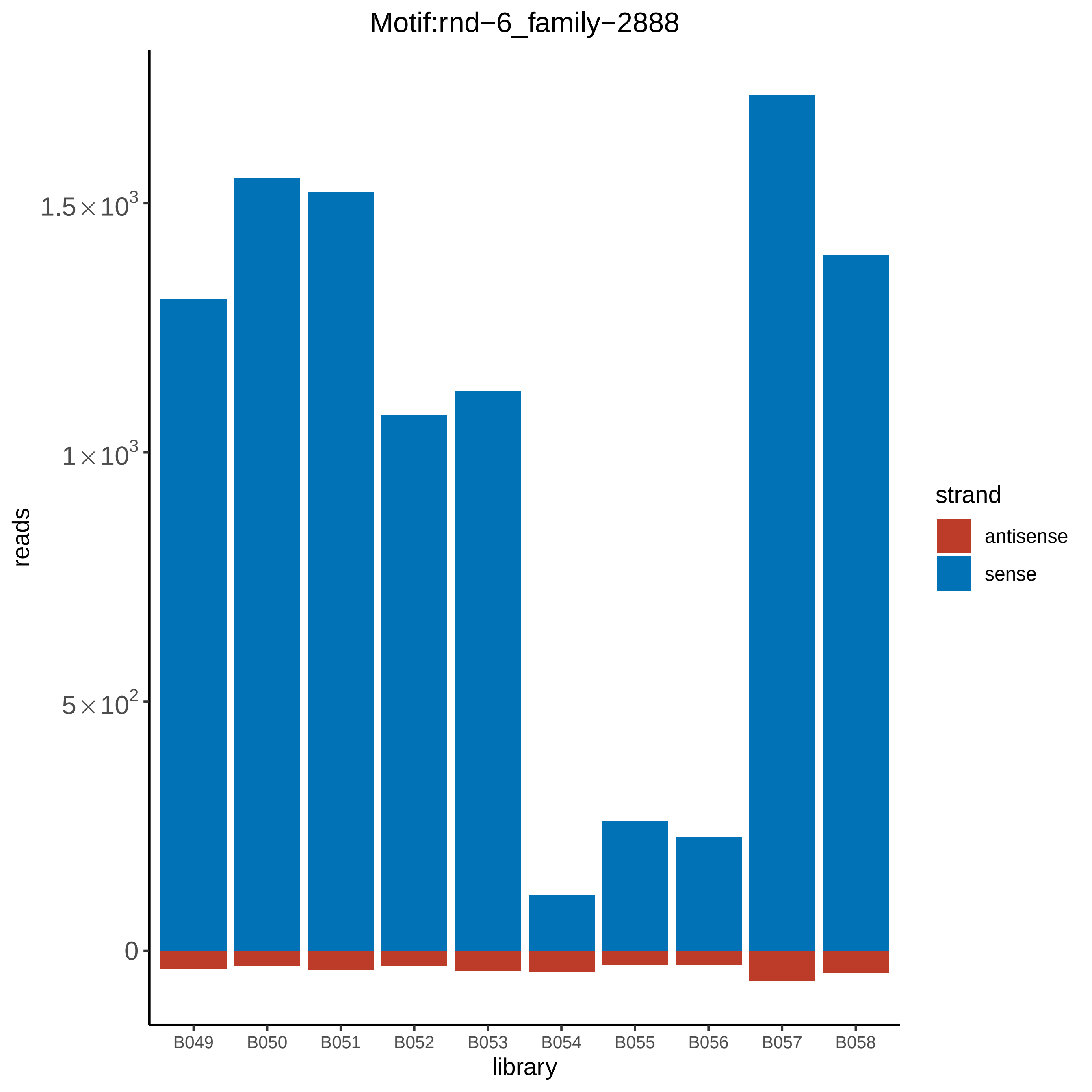

Motif:rnd−6_family−2888
reads
1.5×10^3
1×10^3
5×10^2
0
B049
B050
B051
B052
B053
B054
B055
B056
B057
B058
library
strand
antisense
sense

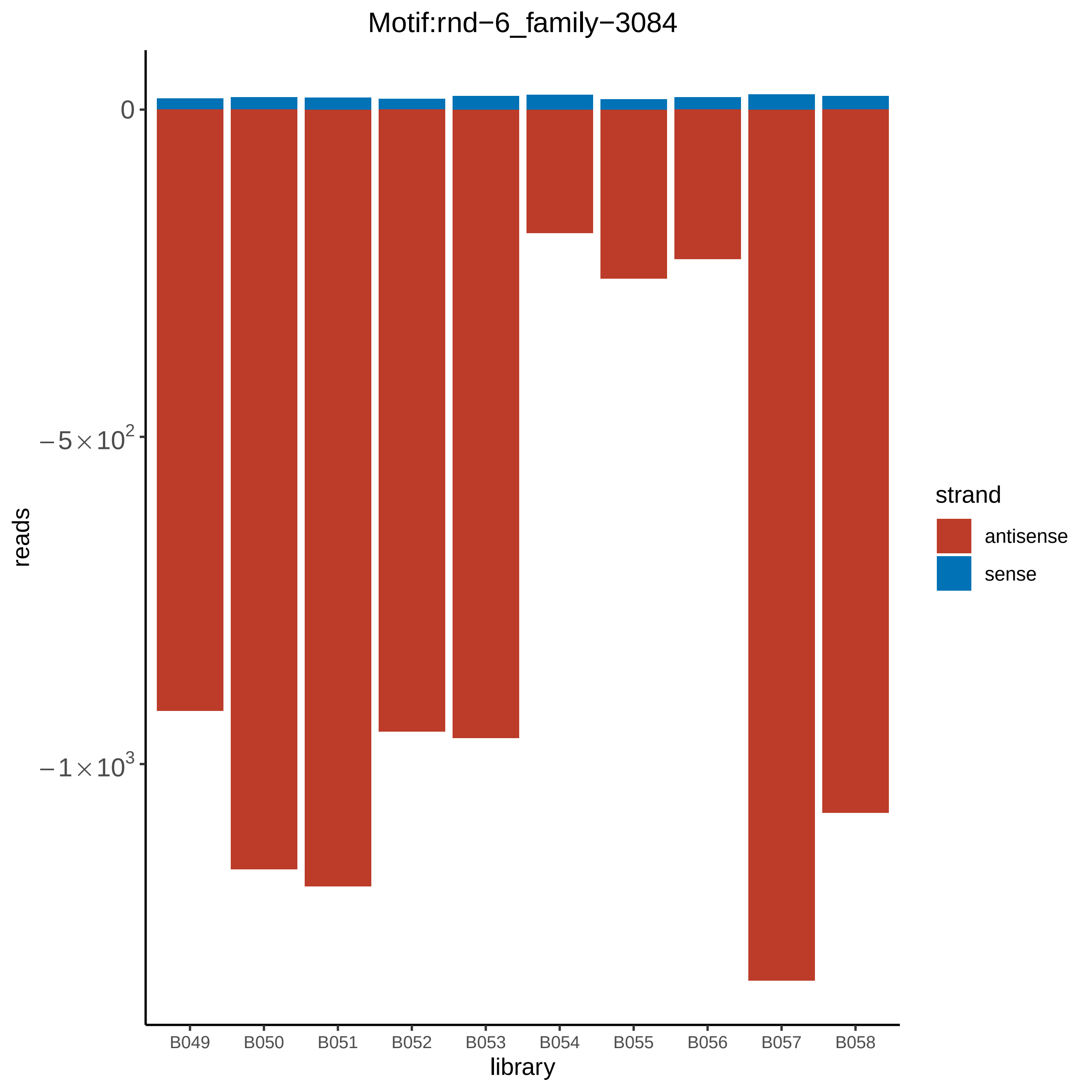

Motif:rnd−6_family−3084
0
−5×10^2
−1×10^3
reads
B049
B050
B051
B052
B053
B054
B055
B056
B057
B058
library
strand
antisense
sense

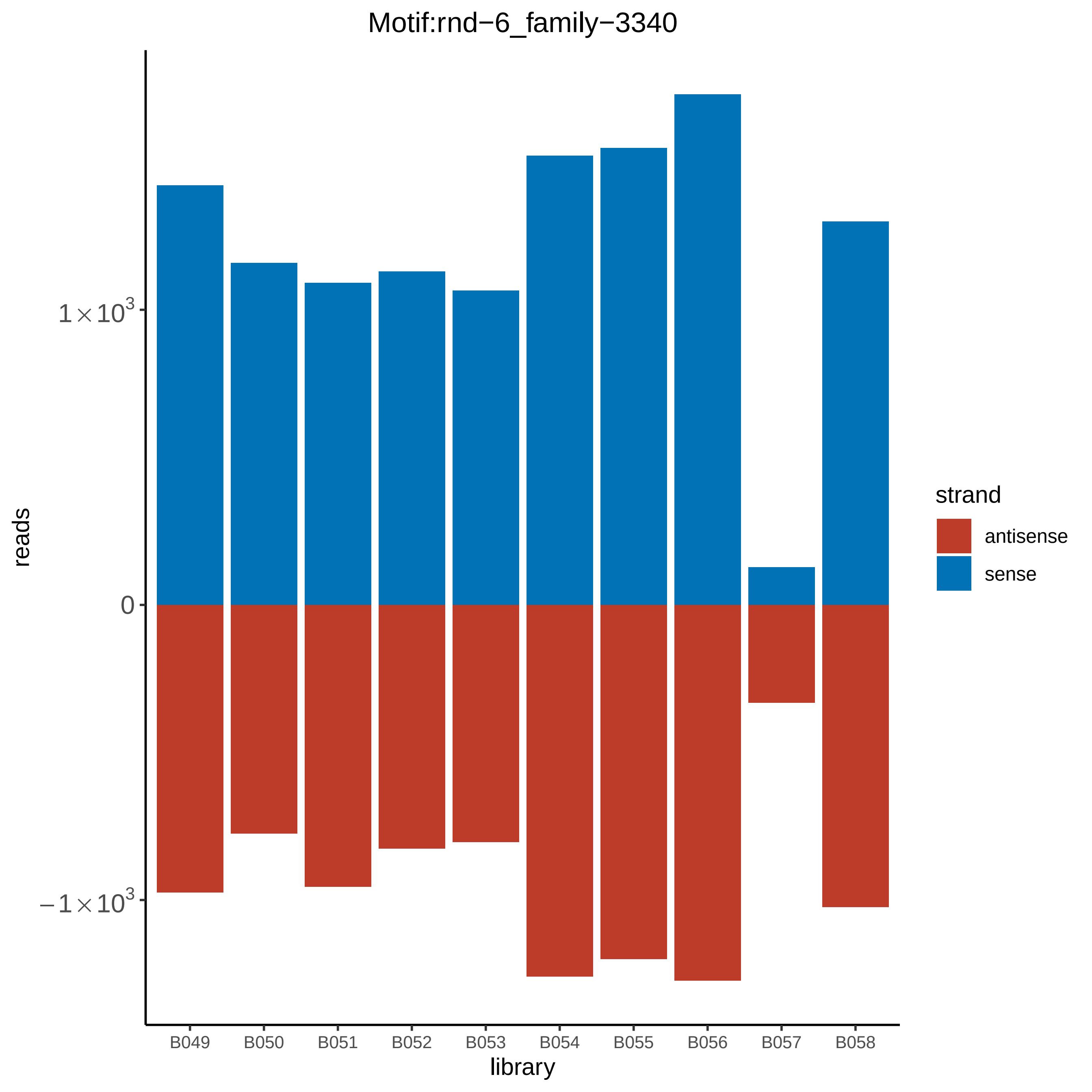

Motif:rnd−6_family−3340
reads
1×10^3
0
−1×10^3
B049
B050
B051
B052
B053
B054
B055
B056
B057
B058
library
strand
antisense
sense

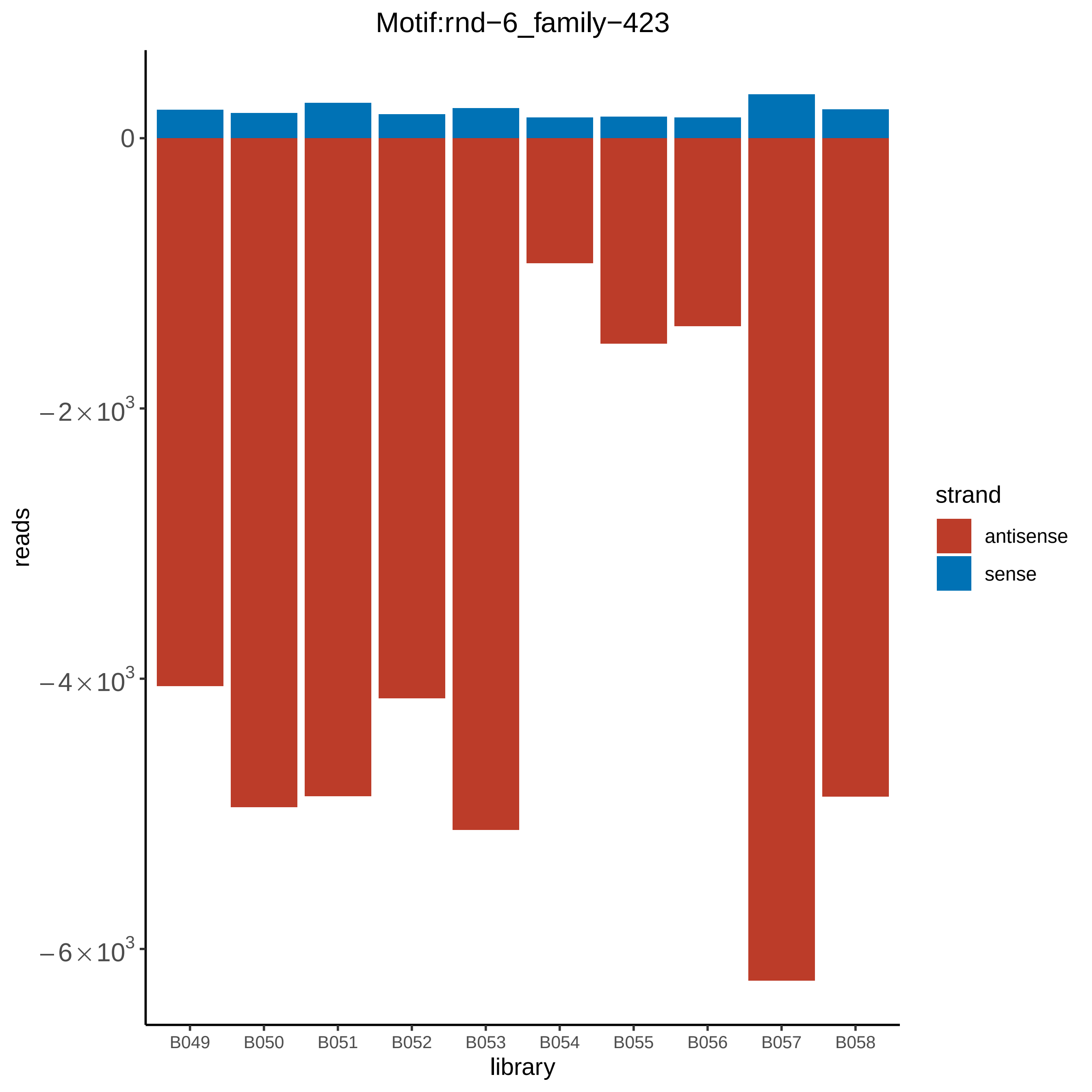

Motif:rnd−6_family−423
0
-2×10^3
-4×10^3
-6×10^3
reads
B049
B050
B051
B052
B053
B054
B055
B056
B057
B058
library
strand
antisense
sense

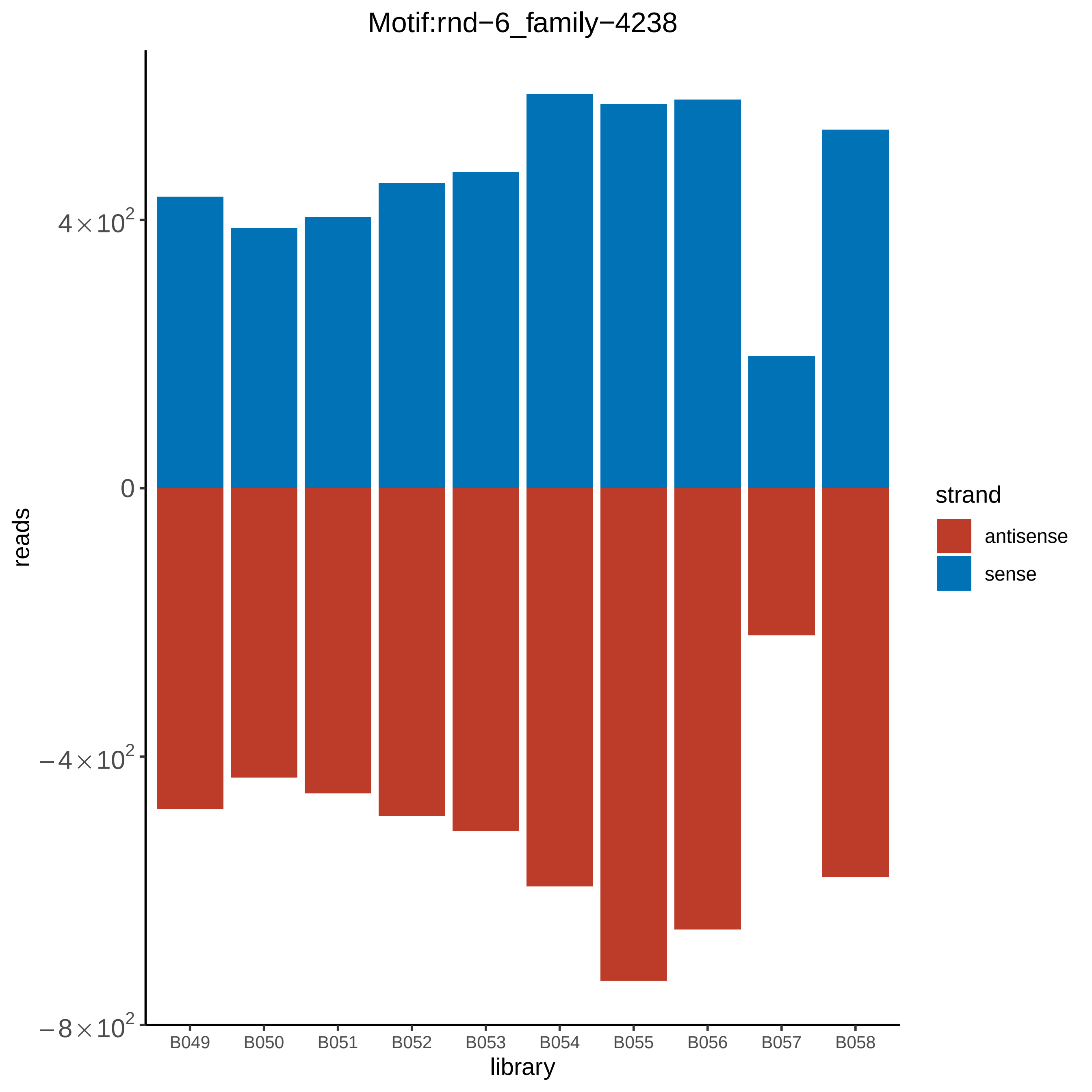

Motif:rnd−6_family−4238
reads
4×10^2
0
-4×10^2
-8×10^2
library
B049
B050
B051
B052
B053
B054
B055
B056
B057
B058
strand
antisense
sense

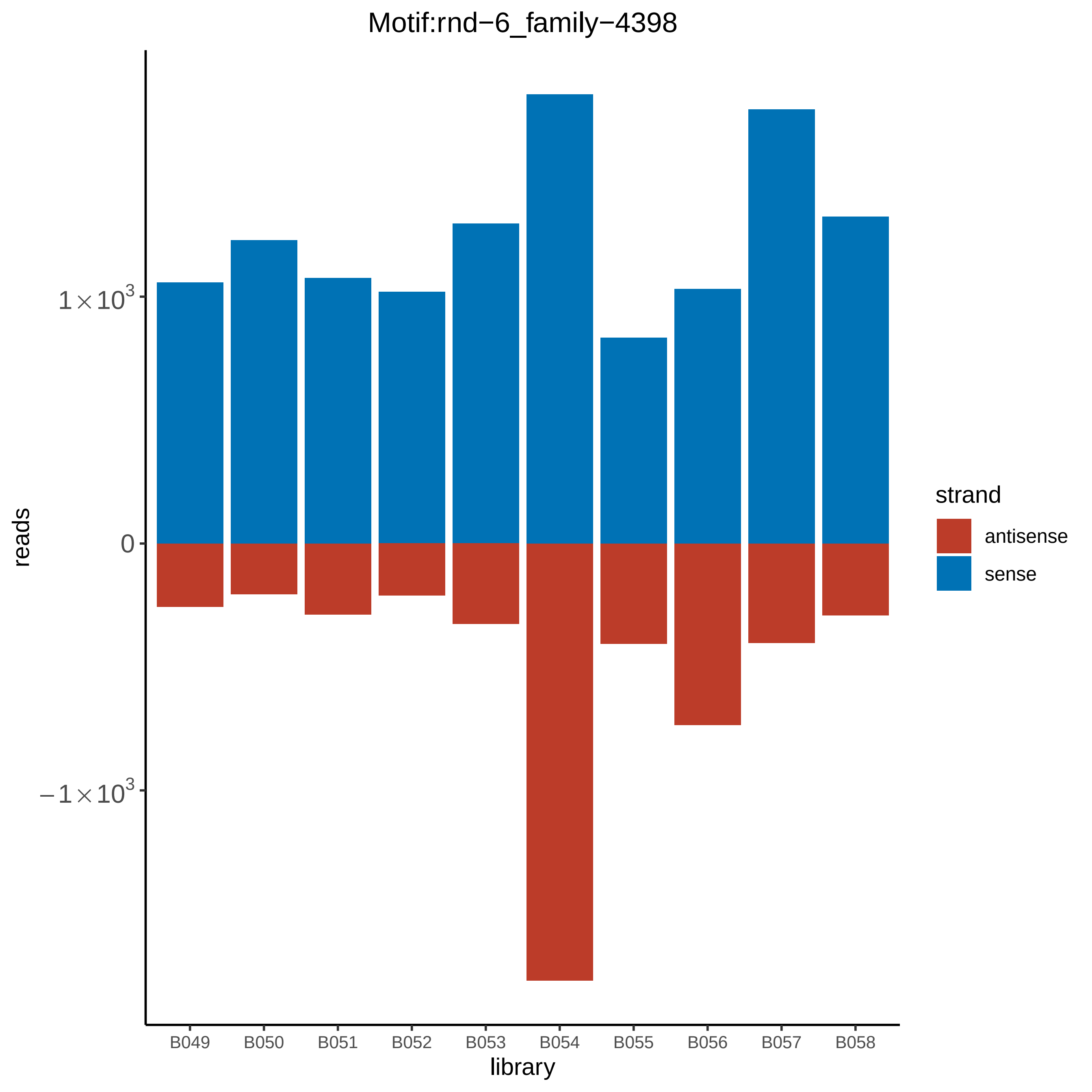
Motif:rnd−6_family−4398
reads
1×10^3
0
−1×10^3
library
B049
B050
B051
B052
B053
B054
B055
B056
B057
B058
strand
antisense
sense

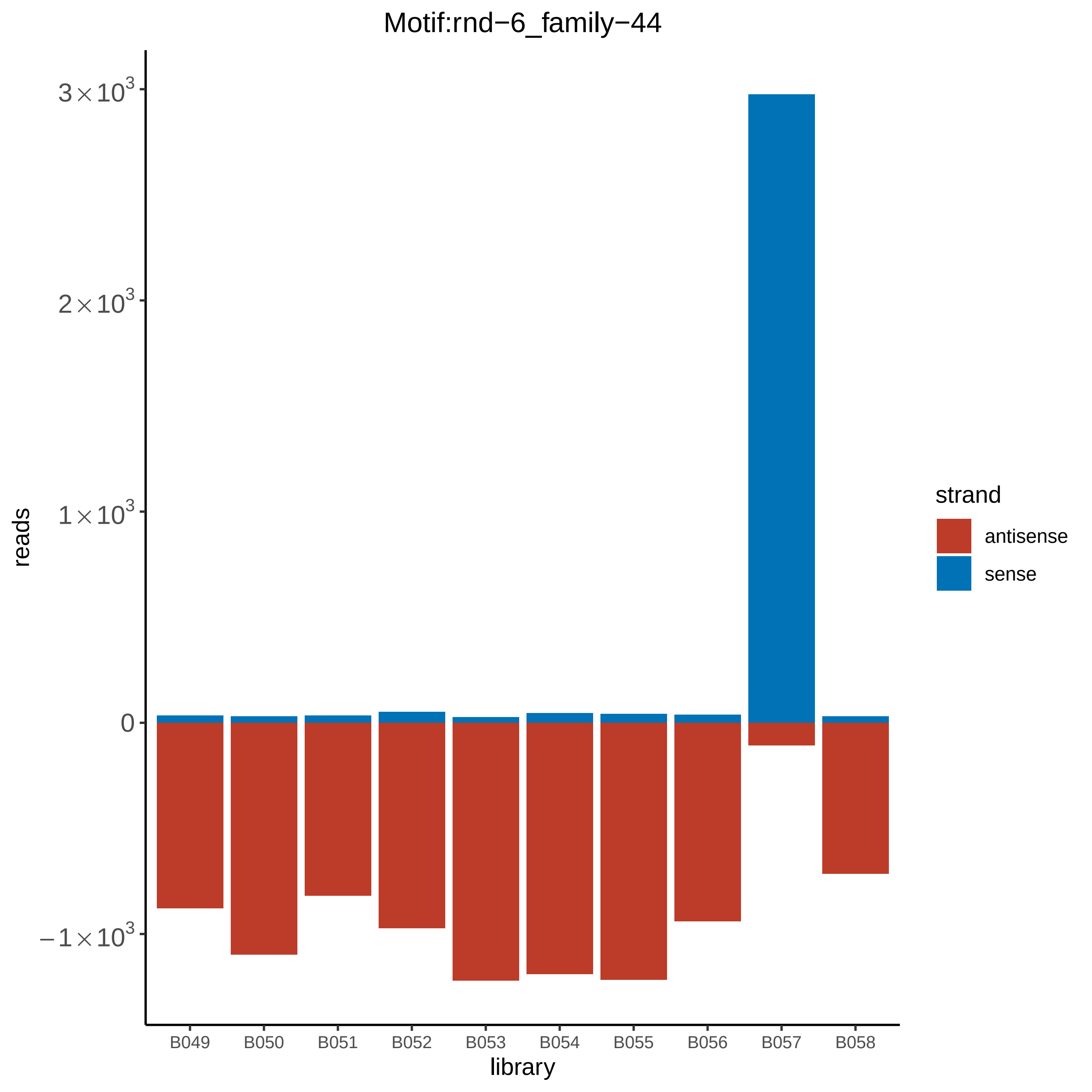
Motif:rnd−6_family−44
reads
3×10^3
2×10^3
1×10^3
0
-1×10^3
library
B049
B050
B051
B052
B053
B054
B055
B056
B057
B058
strand
antisense
sense

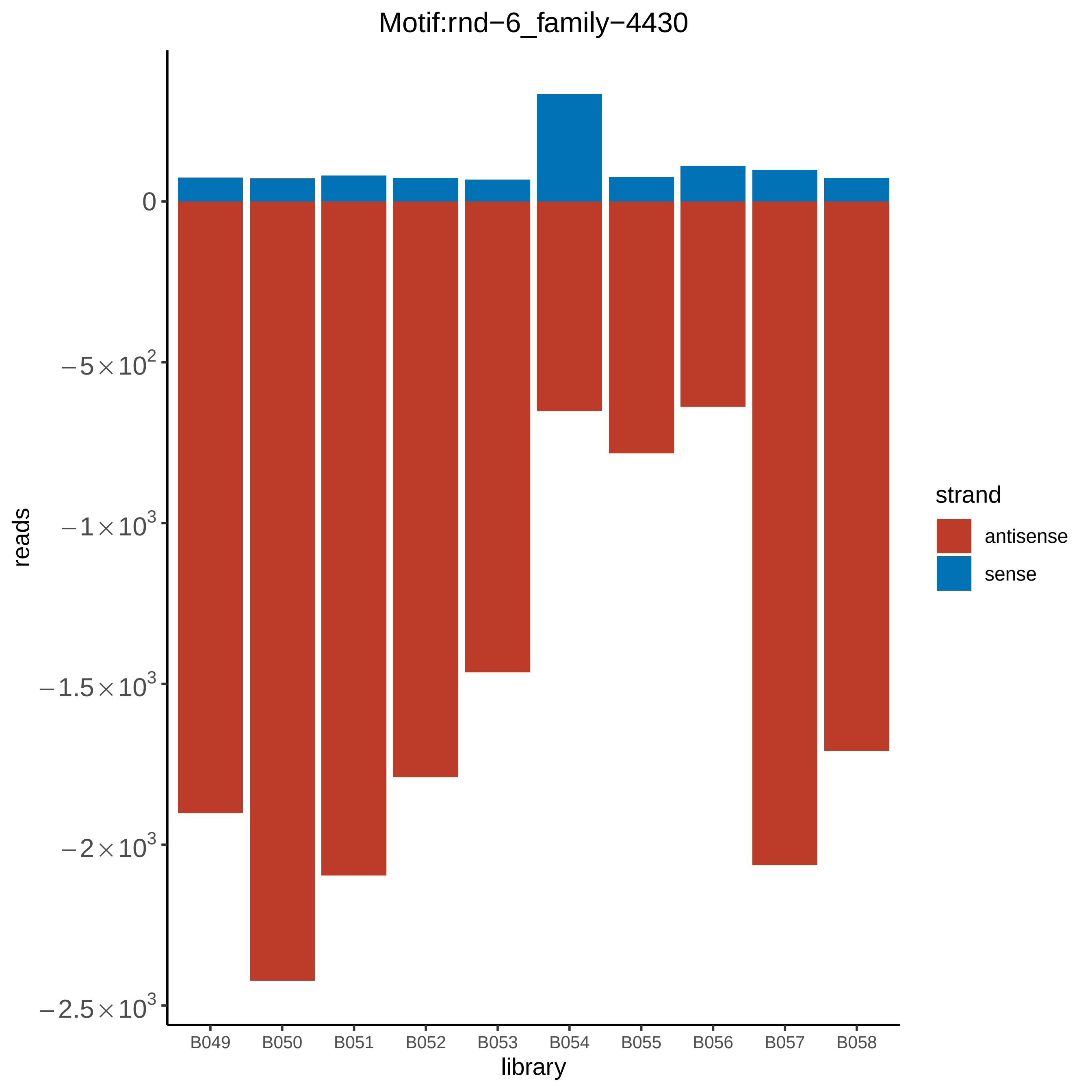

Motif:rnd−6_family−4430
0
−5×10^2
−1×10^3
−1.5×10^3
−2×10^3
−2.5×10^3
reads
B049
B050
B051
B052
B053
B054
B055
B056
B057
B058
library
strand
antisense
sense

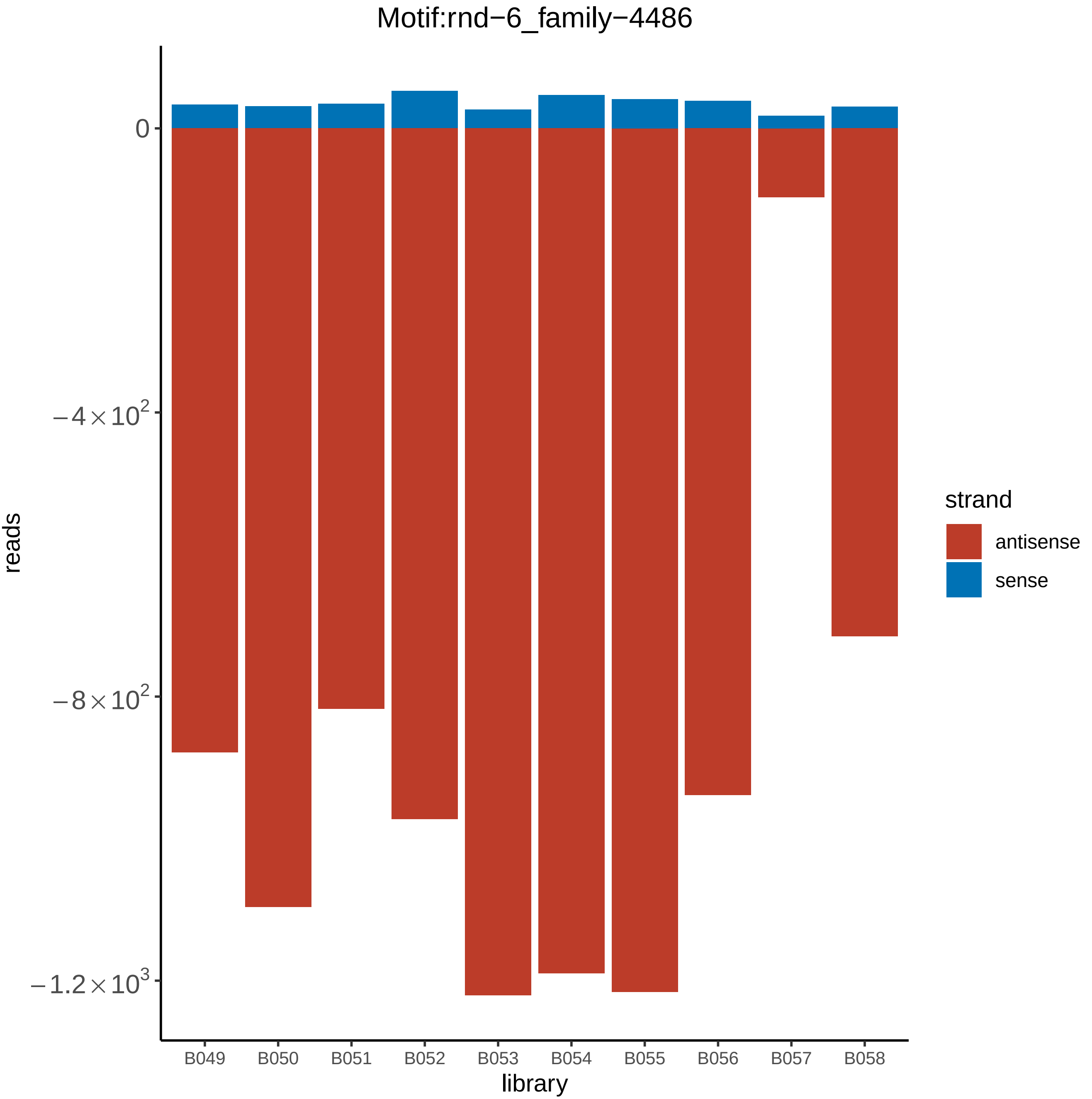

Motif:rnd−6_family−4486
0
−4×10^2
−8×10^2
−1.2×10^3
reads
B049
B050
B051
B052
B053
B054
B055
B056
B057
B058
library
strand
antisense
sense

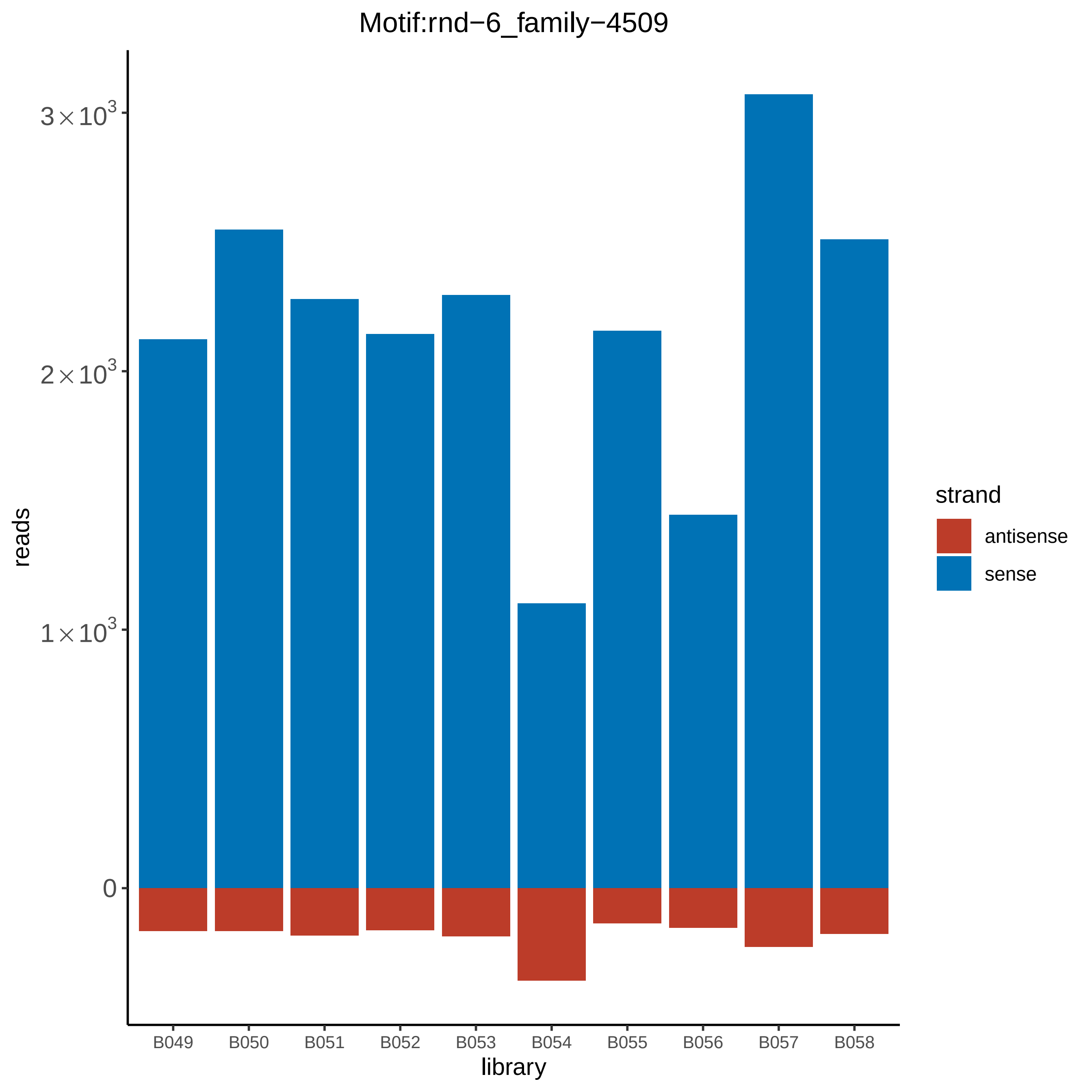

Motif:rnd−6_family−4509
reads
3×10^3
2×10^3
1×10^3
0
library
B049
B050
B051
B052
B053
B054
B055
B056
B057
B058
strand
antisense
sense

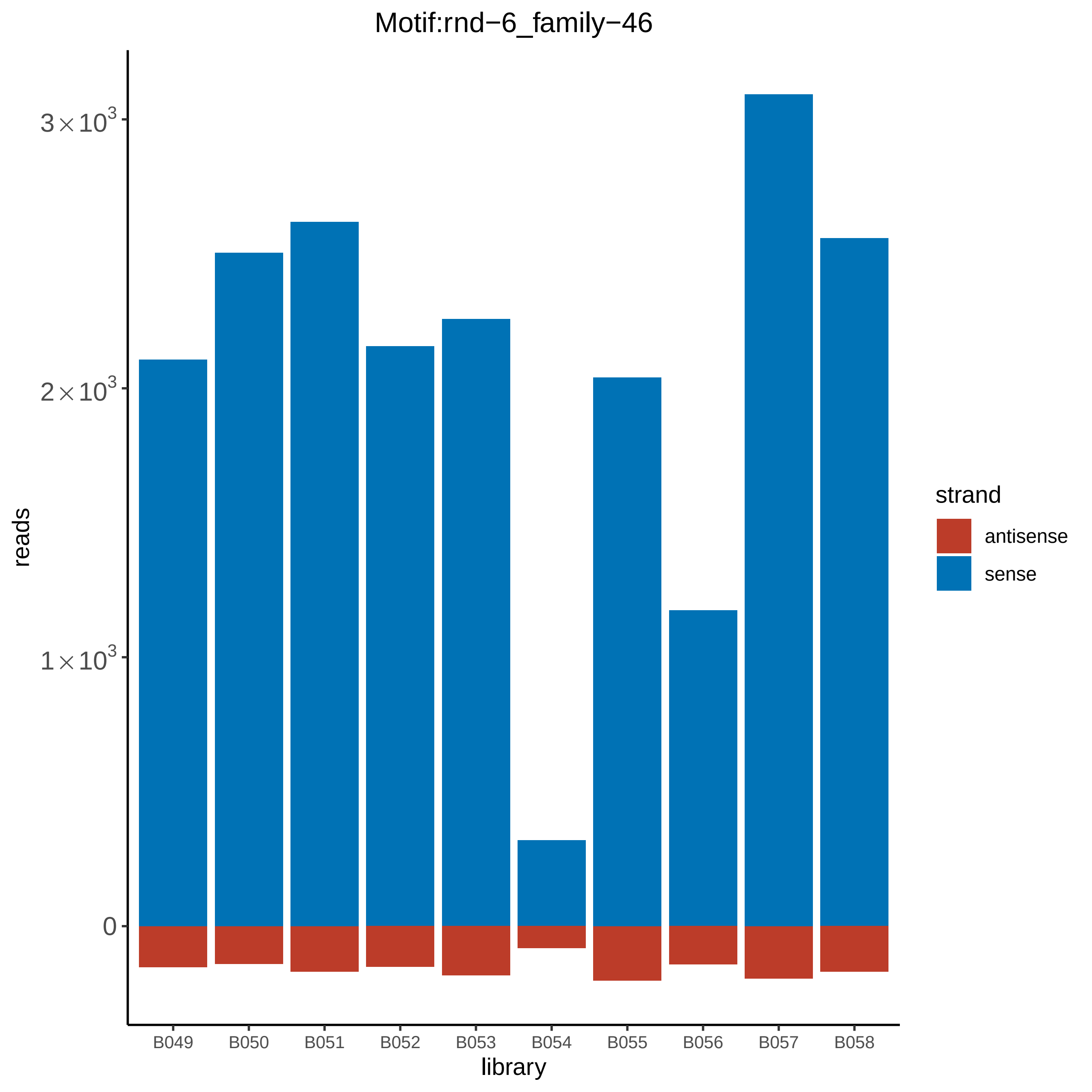

Motif:rnd−6_family−46
reads
3×10^3
2×10^3
1×10^3
0
library
B049
B050
B051
B052
B053
B054
B055
B056
B057
B058
strand
antisense
sense

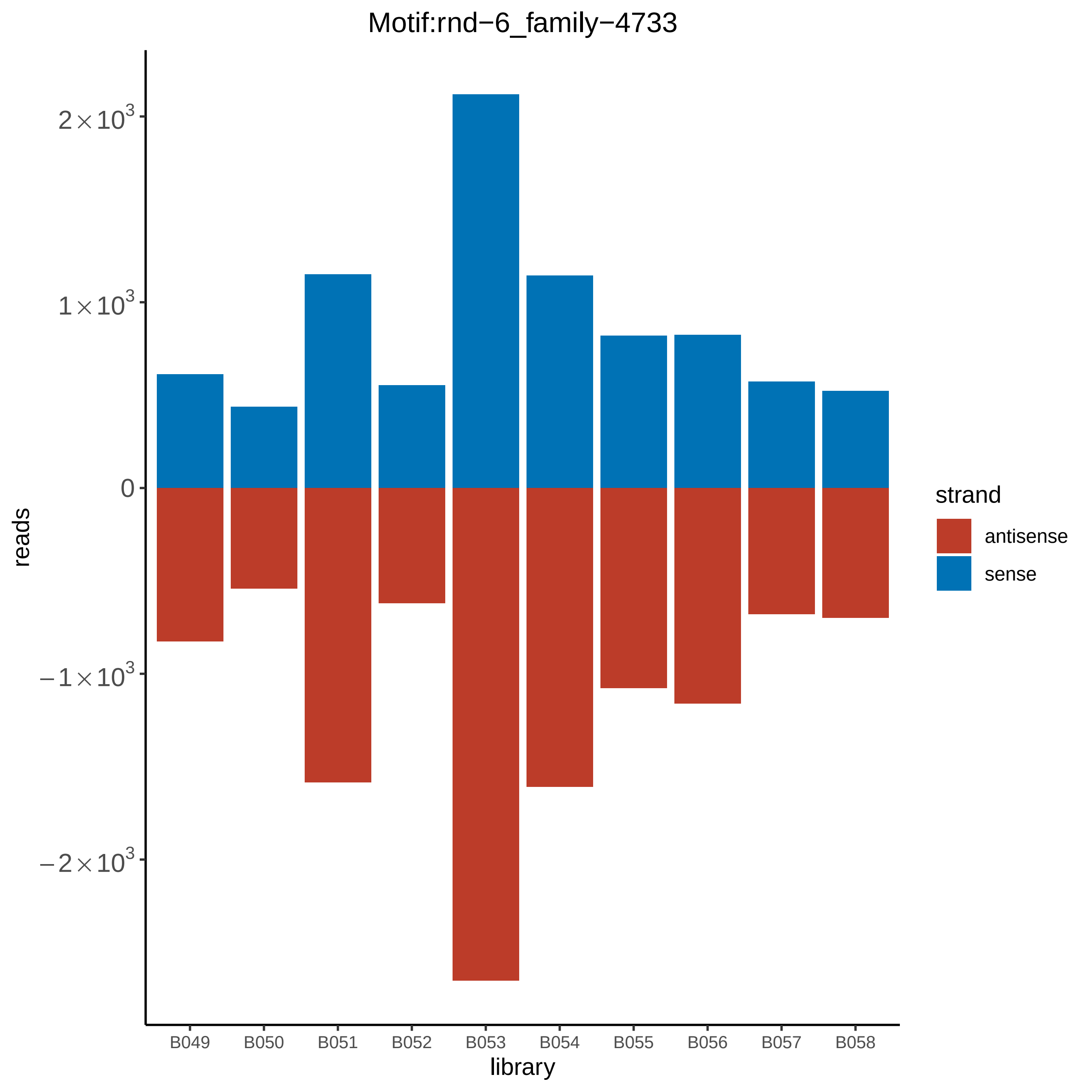

Motif:rnd−6_family−4733
reads
2×10^3
1×10^3
0
-1×10^3
-2×10^3
B049
B050
B051
B052
B053
B054
B055
B056
B057
B058
library
strand
antisense
sense

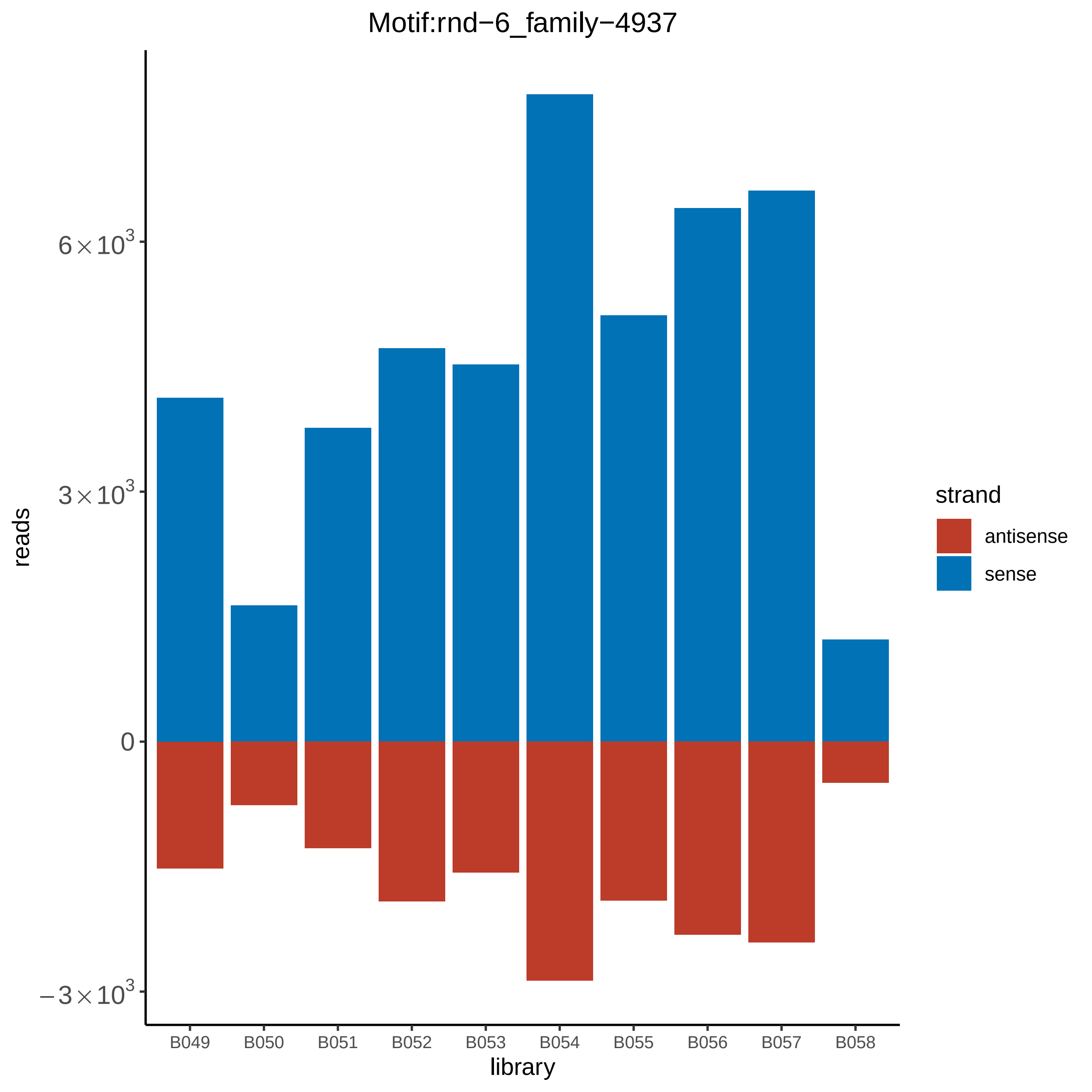

Motif:rnd−6_family−4937
reads
6×10^3
3×10^3
0
-3×10^3
B049
B050
B051
B052
B053
B054
B055
B056
B057
B058
library
strand
antisense
sense

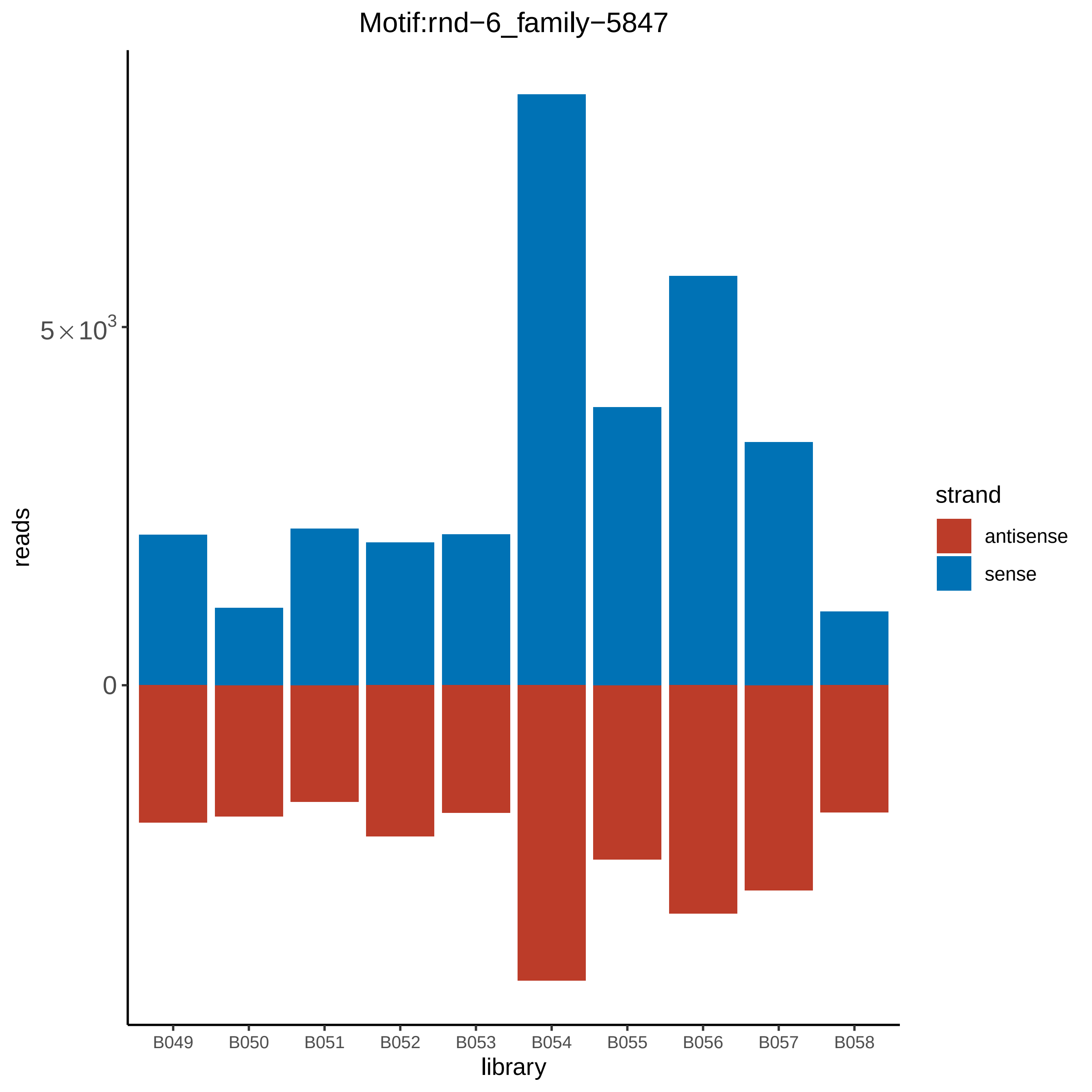

Motif:rnd−6_family−5847
reads
5×10^3
0
library
B049
B050
B051
B052
B053
B054
B055
B056
B057
B058
strand
antisense
sense

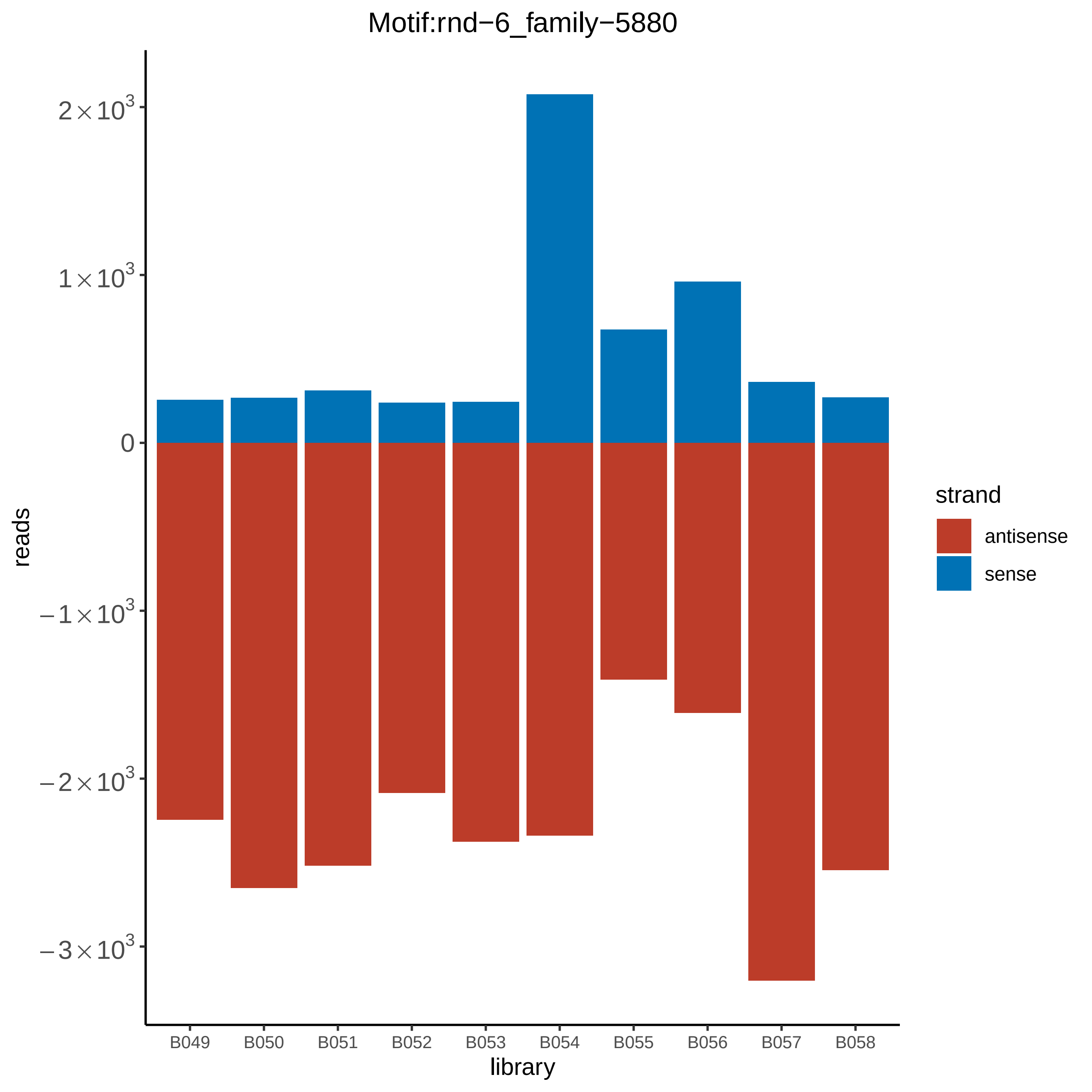

Motif:rnd−6_family−5880
reads
2×10^3
1×10^3
0
-1×10^3
-2×10^3
-3×10^3
B049
B050
B051
B052
B053
B054
B055
B056
B057
B058
library
strand
antisense
sense

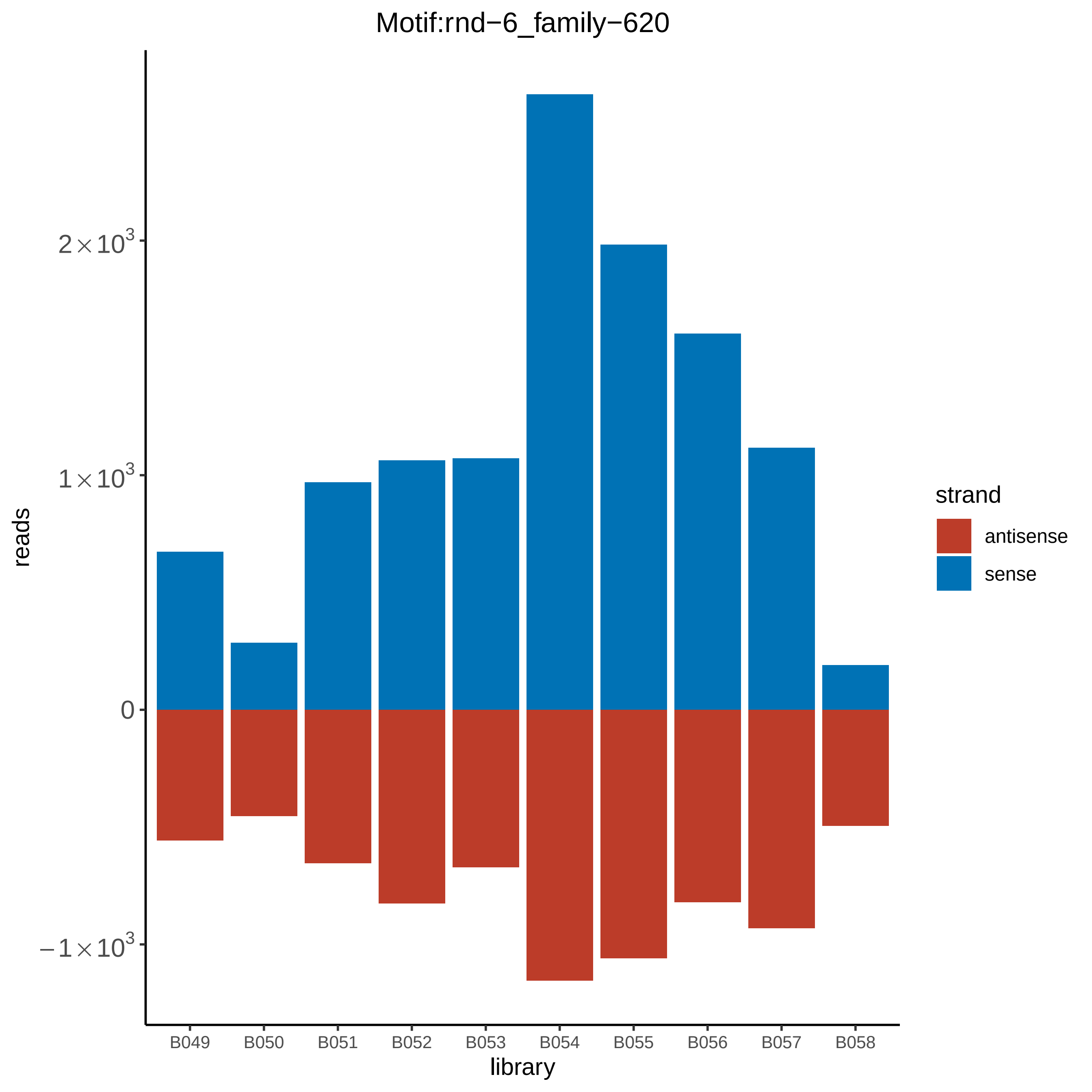
Motif:rnd−6_family−620
reads
2×10^3
1×10^3
0
−1×10^3
library
B049
B050
B051
B052
B053
B054
B055
B056
B057
B058
strand
antisense
sense

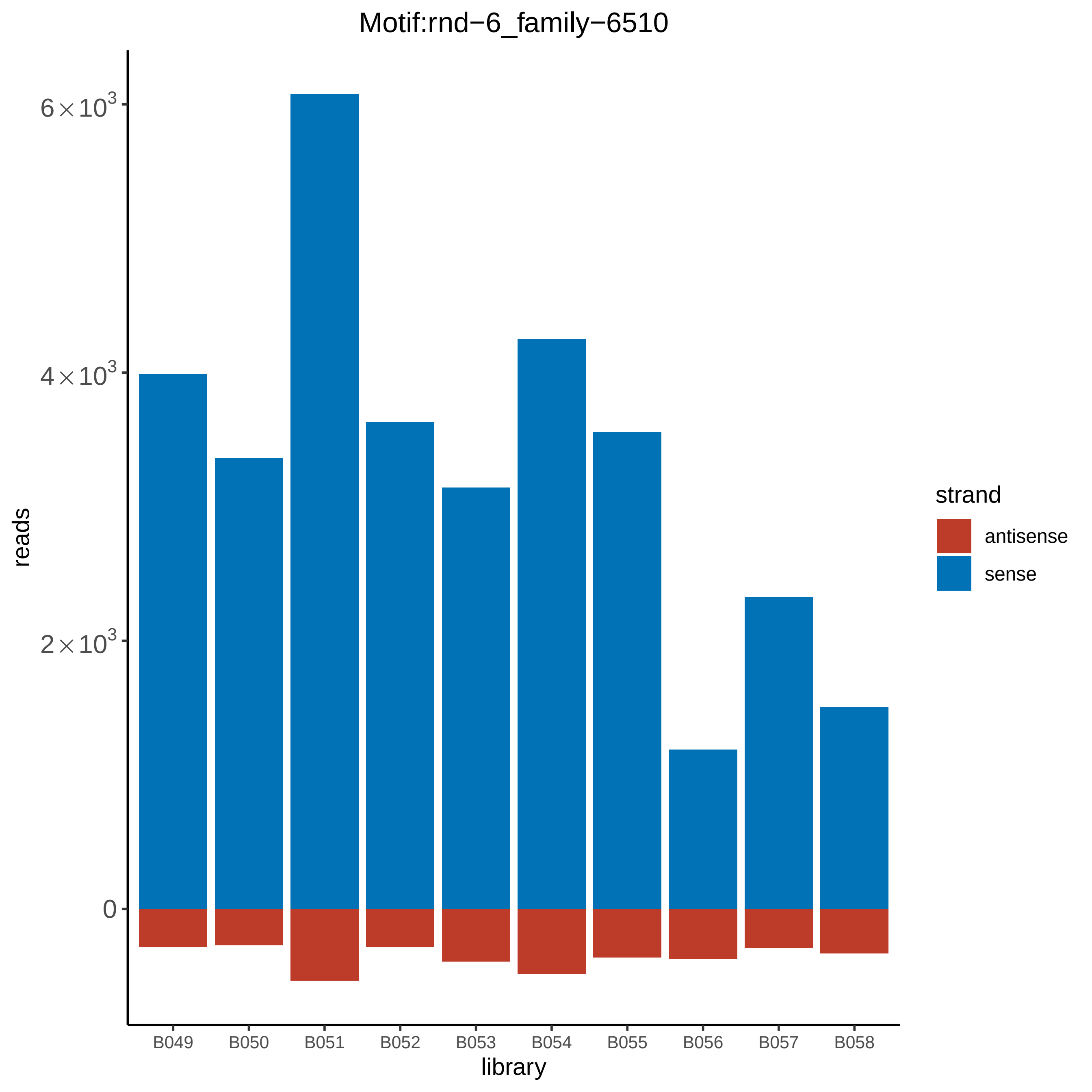
Motif:rnd−6_family−6510
reads
6×10^3
4×10^3
2×10^3
0
library
B049
B050
B051
B052
B053
B054
B055
B056
B057
B058
strand
antisense
sense

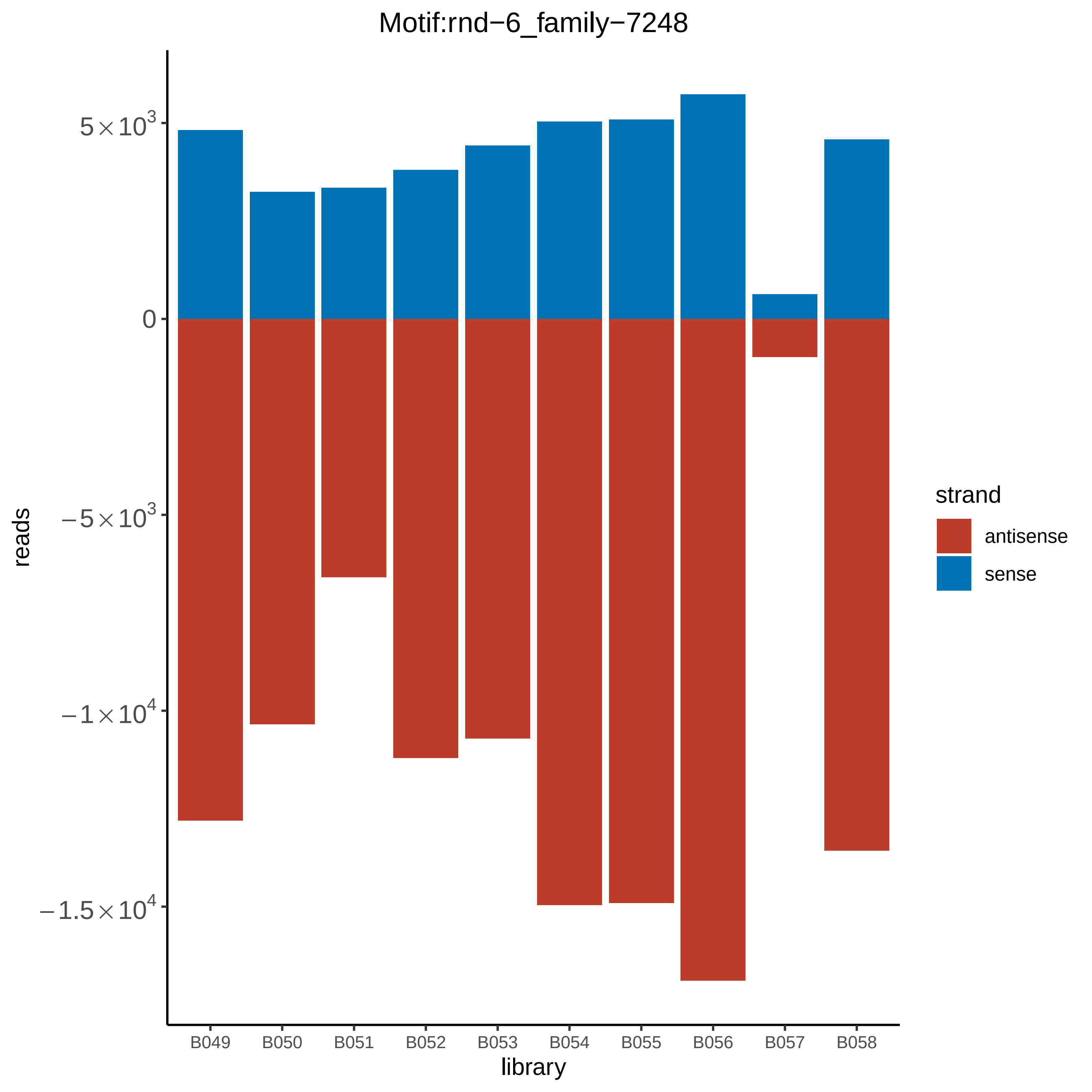
Motif:rnd−6_family−7248
reads
5×10^3
0
−5×10^3
−1×10^4
−1.5×10^4
B049
B050
B051
B052
B053
B054
B055
B056
B057
B058
library
strand
antisense
sense

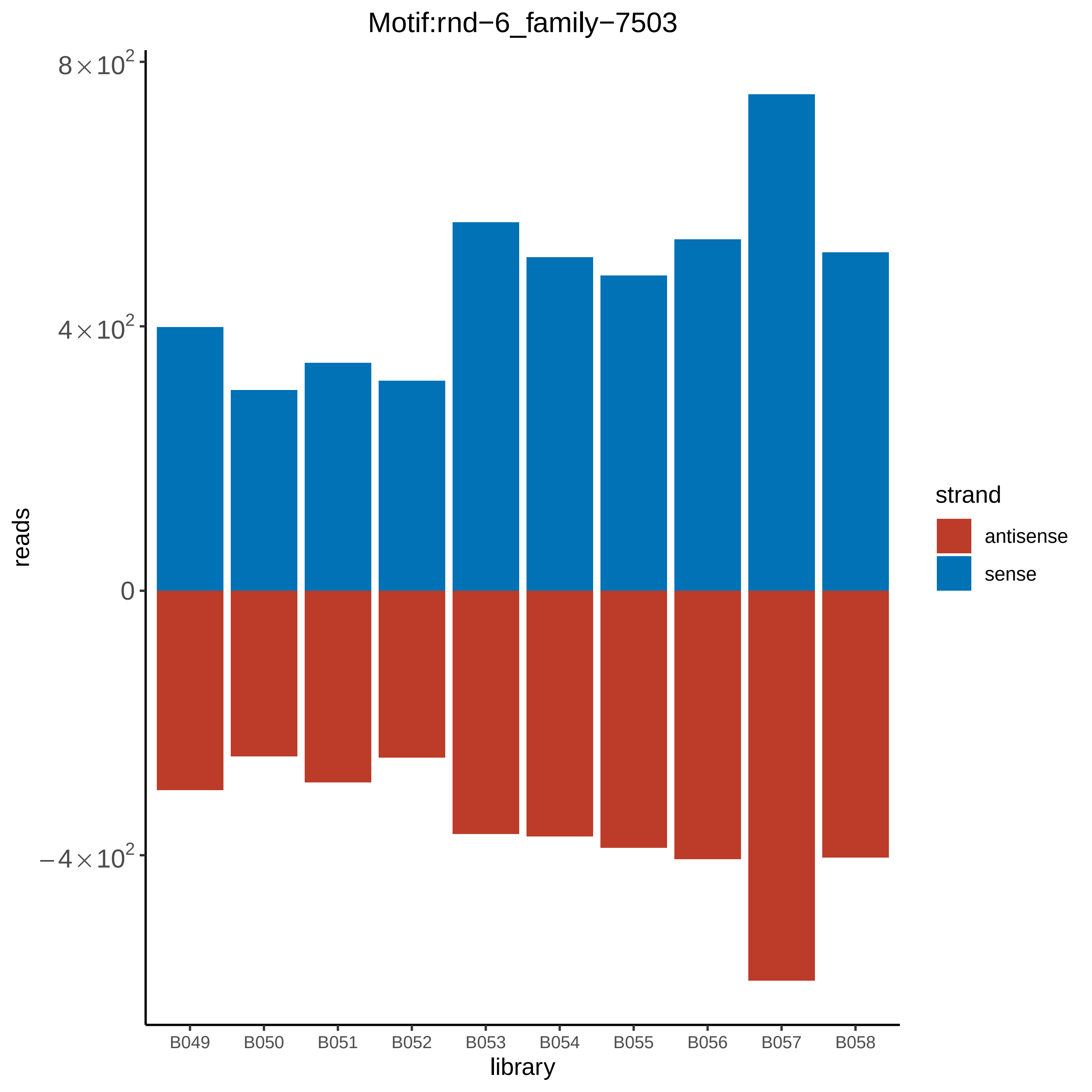
Motif:rnd−6_family−7503
reads
library
8×10^2
4×10^2
0
-4×10^2
B049
B050
B051
B052
B053
B054
B055
B056
B057
B058
strand
antisense
sense

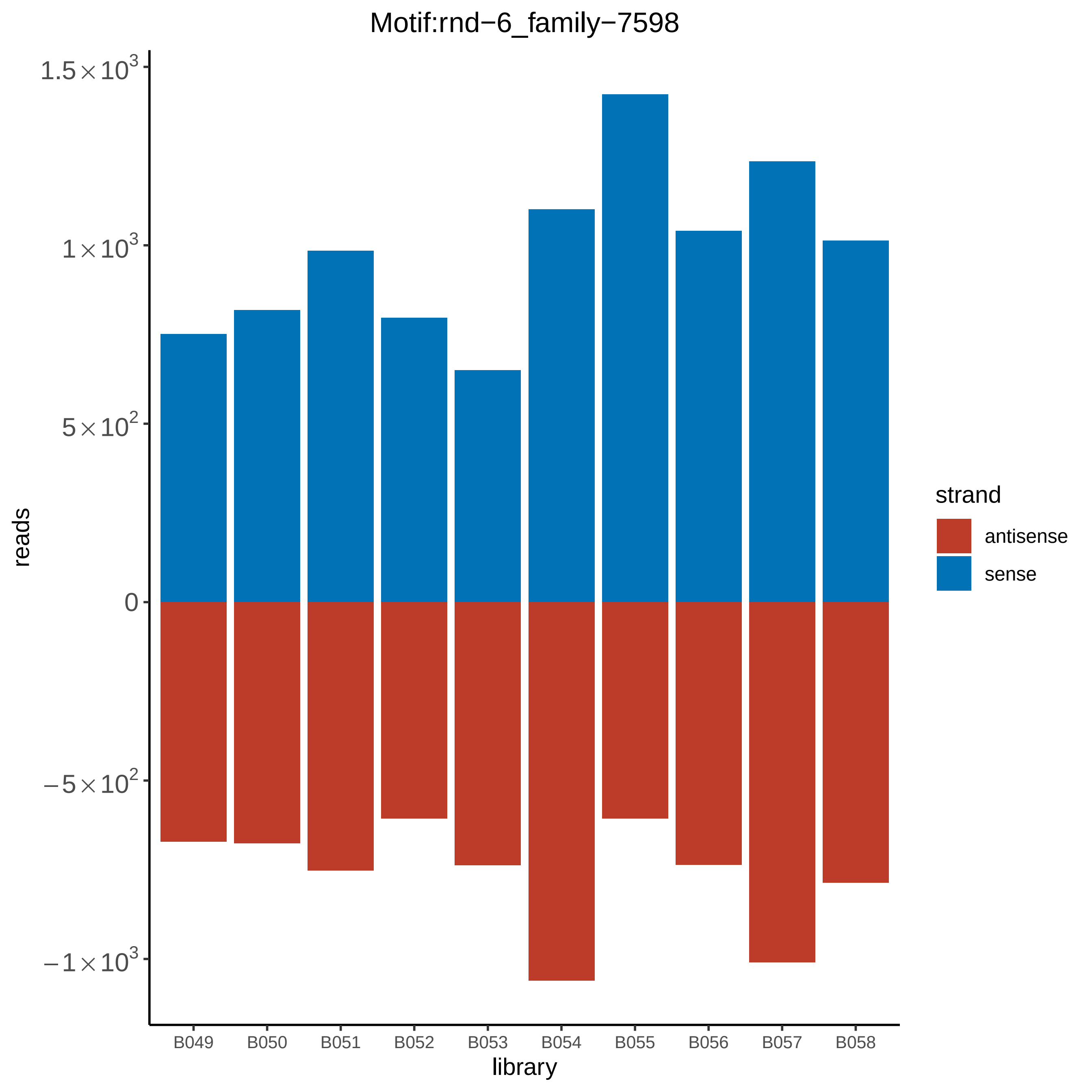
Motif:rnd−6_family−7598
1.5×10^3
1×10^3
5×10^2
0
−5×10^2
−1×10^3
reads
B049
B050
B051
B052
B053
B054
B055
B056
B057
B058
library
strand
antisense
sense

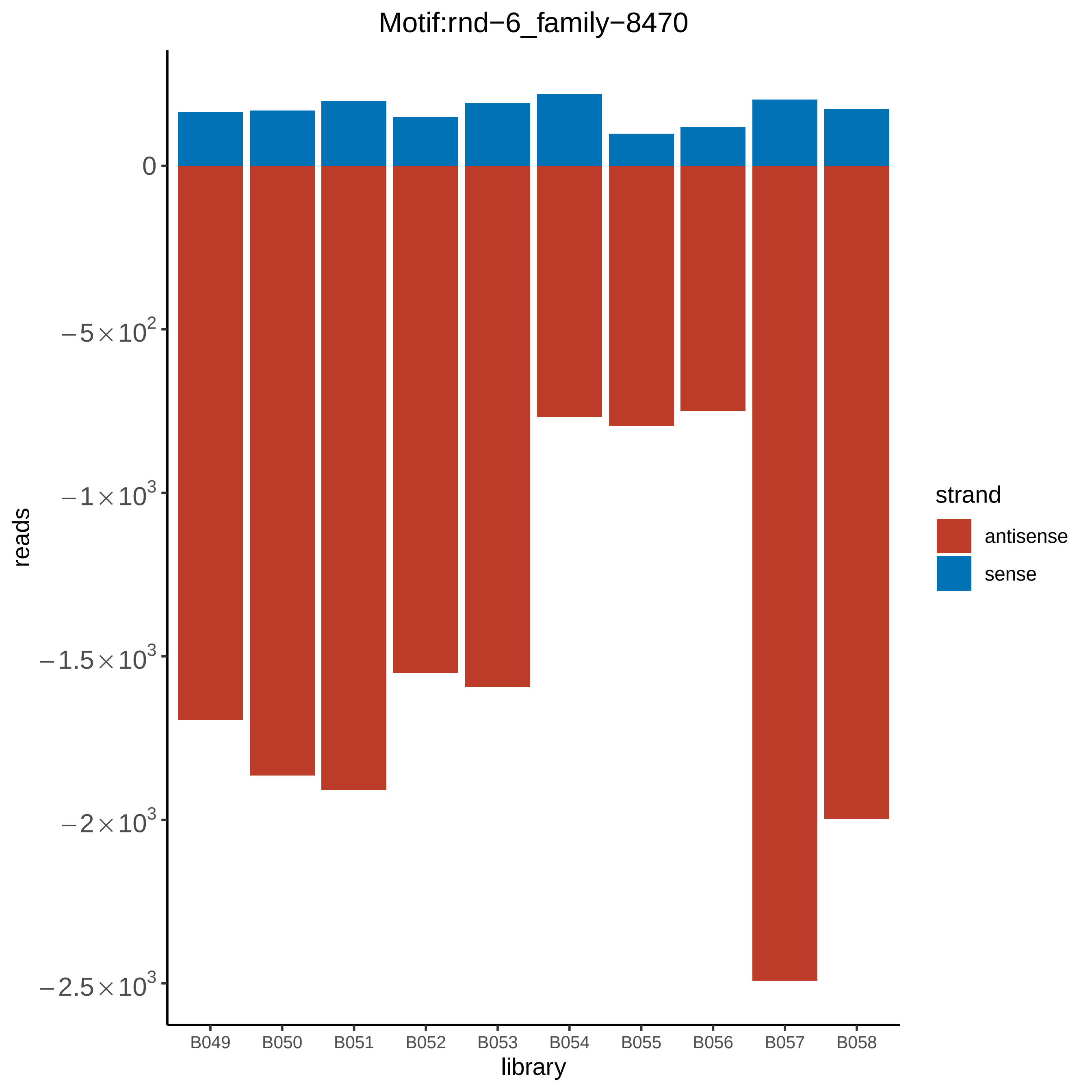

Motif:rnd−6_family−8470
reads
0
−5×10^2
−1×10^3
−1.5×10^3
−2×10^3
−2.5×10^3
library
B049
B050
B051
B052
B053
B054
B055
B056
B057
B058
strand
antisense
sense

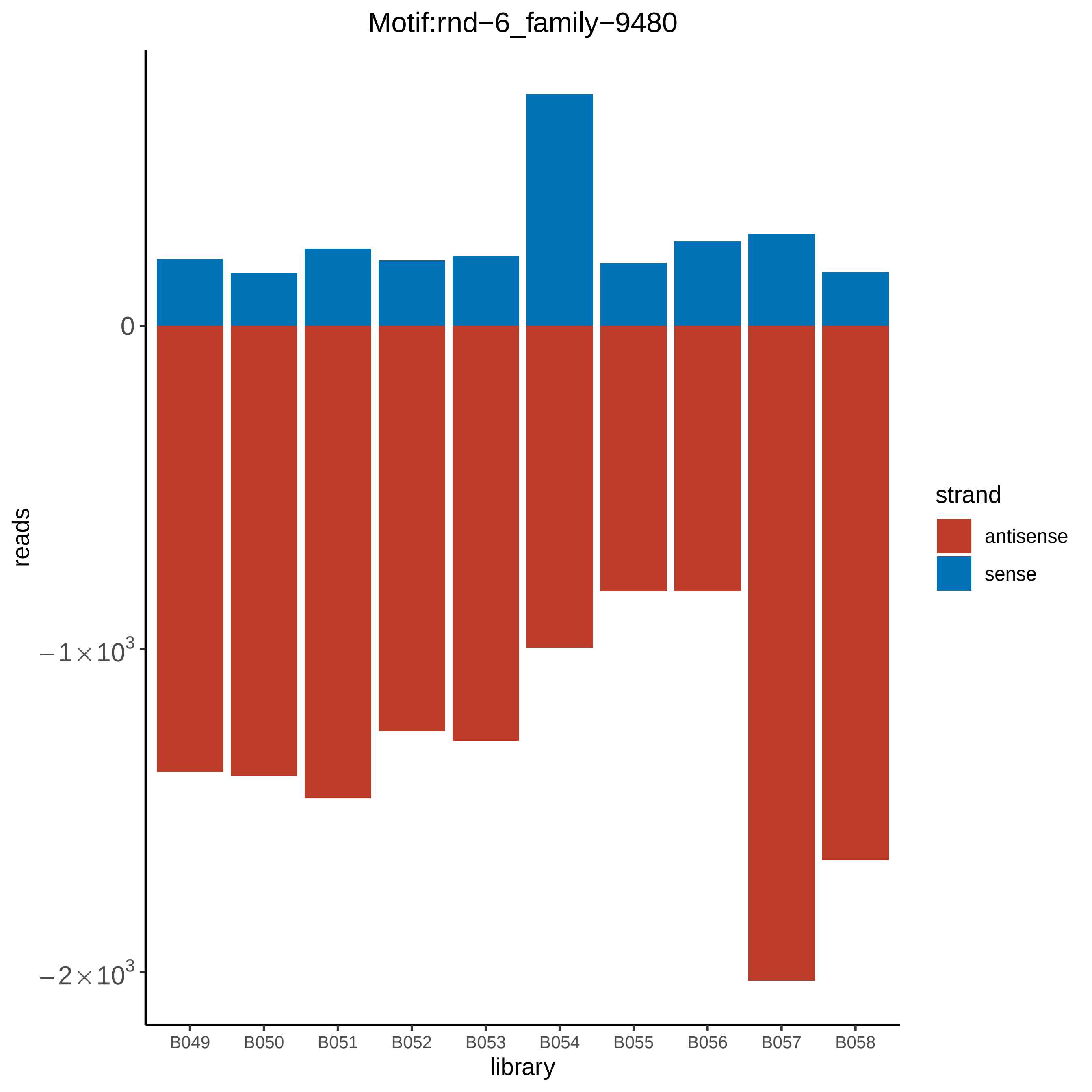

Motif:rnd−6_family−9480
0
−1×10^3
−2×10^3
reads
B049
B050
B051
B052
B053
B054
B055
B056
B057
B058
library
strand
antisense
sense

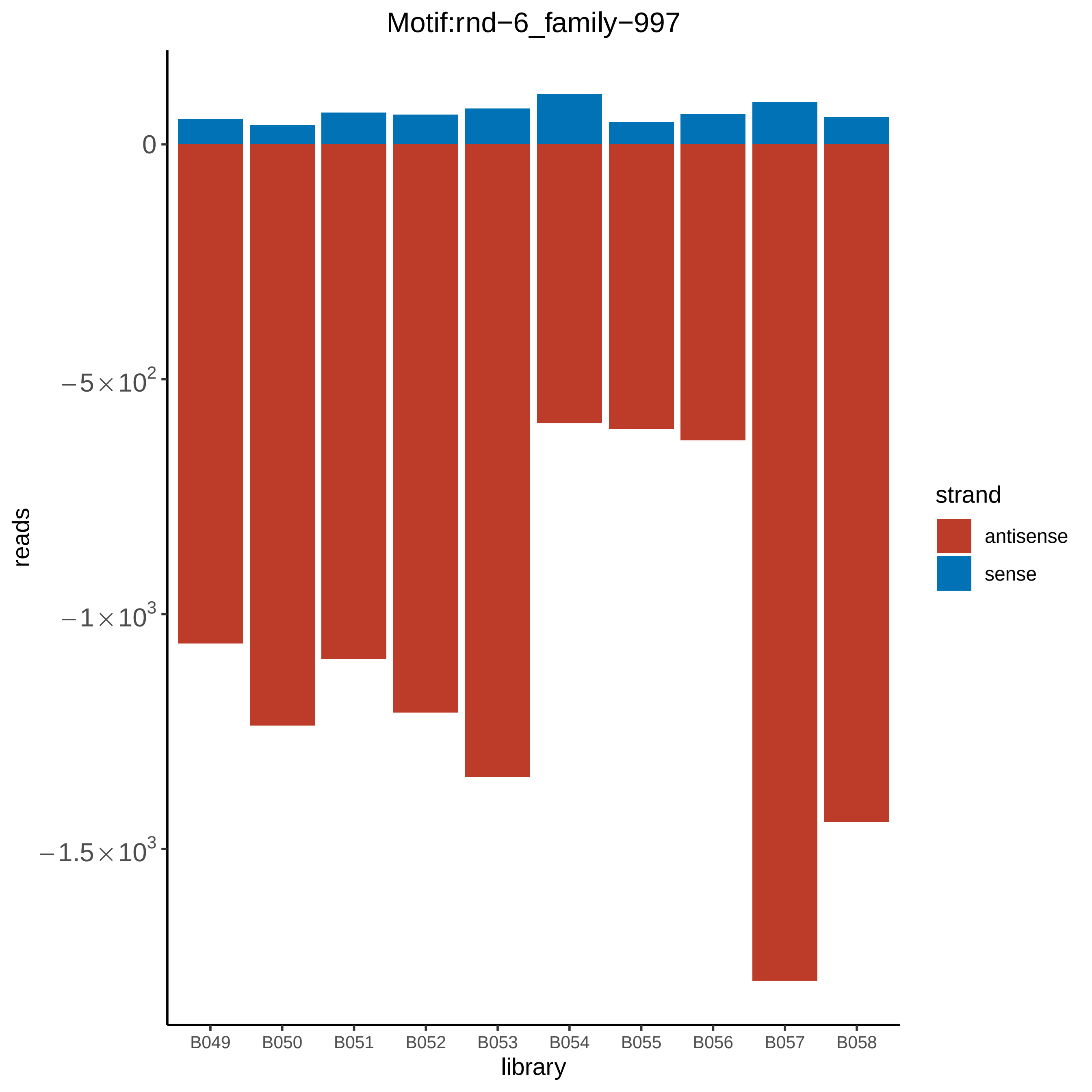

Motif:rnd−6_family−997
0
−5×10^2
−1×10^3
−1.5×10^3
reads
B049
B050
B051
B052
B053
B054
B055
B056
B057
B058
library
strand
antisense
sense
